# Cycloalkane Incorporation Into the 2′,4′-Bridge of Locked Nucleic Acid: Enhancing Nuclease Stability, Reducing Phosphorothioate Modifications, and Lowering Hepatotoxicity in Antisense Oligonucleotides

**DOI:** 10.1021/jacsau.5c01005

**Published:** 2025-10-15

**Authors:** Takao Yamaguchi, Hibiki Komine, Takaya Sugiura, Riku Kumagai, Tokuyuki Yoshida, Kiyomi Sasaki, Taisuke Nakayama, Haruhiko Kamada, Takao Inoue, Satoshi Obika

**Affiliations:** † Graduate School of Pharmaceutical Sciences, 13013 The University of Osaka, 1-6 Yamadaoka, Suita, Osaka 565-0871, Japan; ‡ 26360 National Institute of Health Sciences, 3-25-26 Tonomachi, Kawasaki-ku, Kawasaki, Kanagawa 210-9501, Japan; § 53313 National Institutes of Biomedical Innovation, Health and Nutrition, 7-6-8 Saito-Asagi, Ibaraki, Osaka 567-0085, Japan; ∥ Institute for Open and Transdisciplinary Research Initiatives, The University of Osaka, 1-1 Yamadaoka, Suita, Osaka 565-0871, Japan

**Keywords:** antisense oligonucleotides, gapmer, bridged nucleic acids, locked nucleic acid (LNA), phosphorothioate reduction, cycloalkanes, nuclease stability, hepatotoxicity

## Abstract

2′-*O*,4′-*C*-Methylene-bridged nucleic acid (2′,4′-BNA), also known as locked nucleic acid (LNA), is widely used to modify antisense oligonucleotides (ASOs) because it significantly enhances their ability to form duplexes with target RNAs, thereby boosting ASO activity. However, 2′,4′-BNA/LNA exhibits only moderate nuclease stability, necessitating additional modifications, such as phosphorothioate (PS) linkages. Our previous studies demonstrated that oligonucleotides modified with 2′-*O*,4′-*C*-spirocyclopropylene-bridged nucleic acid (scpBNA) retain duplex-forming abilities similar to those of 2′,4′-BNA/LNA, while showing significantly improved nuclease stability. In the present study, we introduce 2′-*O*,4′-*C*-spirocyclopentylene-bridged nucleic acid (scpBNA2), which offers even greater nuclease stability than scpBNA. We synthesized scpBNA2 phosphoramidites containing either thymine or 5-methylcytosine nucleobases and incorporated them into gapmer-type ASOs. Both scpBNA- and scpBNA2-modified ASOs exhibited antisense activity comparable to that of their 2′,4′-BNA/LNA-modified counterparts, maintaining activity even with reduced PS modifications in the scpBNA- or scpBNA2-modified regions. Furthermore, hepatotoxicity observed with 2′,4′-BNA/LNA-modified ASOs was significantly reduced when 2′,4′-BNA/LNA was replaced with either scpBNA or scpBNA2. Overall, these findings underscore the potential of scpBNA and scpBNA2 for use in therapeutic ASOs.

## Graphical abstract

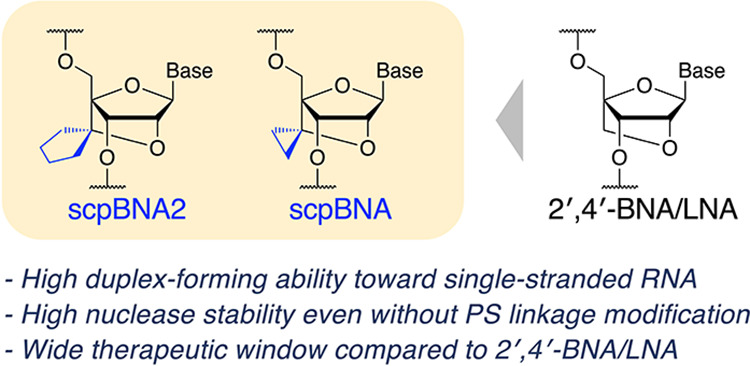

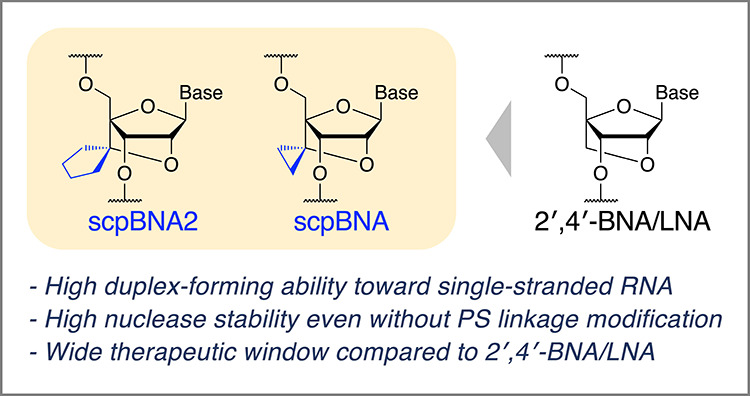

## Introduction

Antisense oligonucleotides (ASOs) have emerged as a promising therapeutic approach for various diseases.^1,2^ Typically, ASOs are 15–25 nucleotides long and are designed to be complementary to target RNA sequences. Chemical modifications are essential to enhance their stability and efficacy both *in vitro* and *in vivo*.^3,4^ Phosphorothioate (PS) linkages are commonly employed to improve nuclease resistance and facilitate cellular uptake.^5,6^ Additionally, PS modifications enhance plasma protein binding, thereby reducing rapid renal excretion and improving pharmacokinetics. However, due to their hydrophobic nature, PS-modified ASOs can interact with various cellular proteins, potentially leading to hepatotoxicity.^5,6^ 2′-*O*,4′-*C*-Methylene-bridged nucleic acid (2′,4′-BNA),^7,8^ also known as locked nucleic acid (LNA),^9,10^ is widely used to modify ASOs because it significantly enhances duplex formation with target RNAs, thereby increasing ASO activity (Figure 1).^11^ However, the nuclease stability of 2′,4′-BNA/LNA is limited, necessitating the combination of these modifications with PS linkages to develop effective ASOs, despite the associated hepatotoxicity risks. To address this issue, modifications at the 2′,4′-bridge have been explored to improve nuclease stability.^12–17^ For instance, 2′,4′-constrained ethyl (cEt) nucleic acids, analogs of 2′,4′-BNA/LNA with a methyl group on the bridge, exhibit significantly enhanced nuclease stability compared to the original 2′,4′-BNA/LNA (Figure 1).^13^ The synthesis of these analogs requires stereocontrolled methods to create the diastereomeric bridge structure. In contrast, we previously developed 2′-*O*,4′-*C*-spirocyclopropylene-bridged nucleic acid (scpBNA; Figure 1), which incorporates a cyclopropane ring without producing a diastereomeric center on the 2′,4′-bridge.^15,16^ Oligonucleotides modified with scpBNA exhibit significantly higher nuclease stability than those modified with 2′,4′-BNA/LNA. The increased nuclease stability of cEt and scpBNA is believed to arise from the steric bulkiness of the pendant methyl and cyclopropane moiety, respectively, which inhibits nuclease recognition.

**Figure 1:**
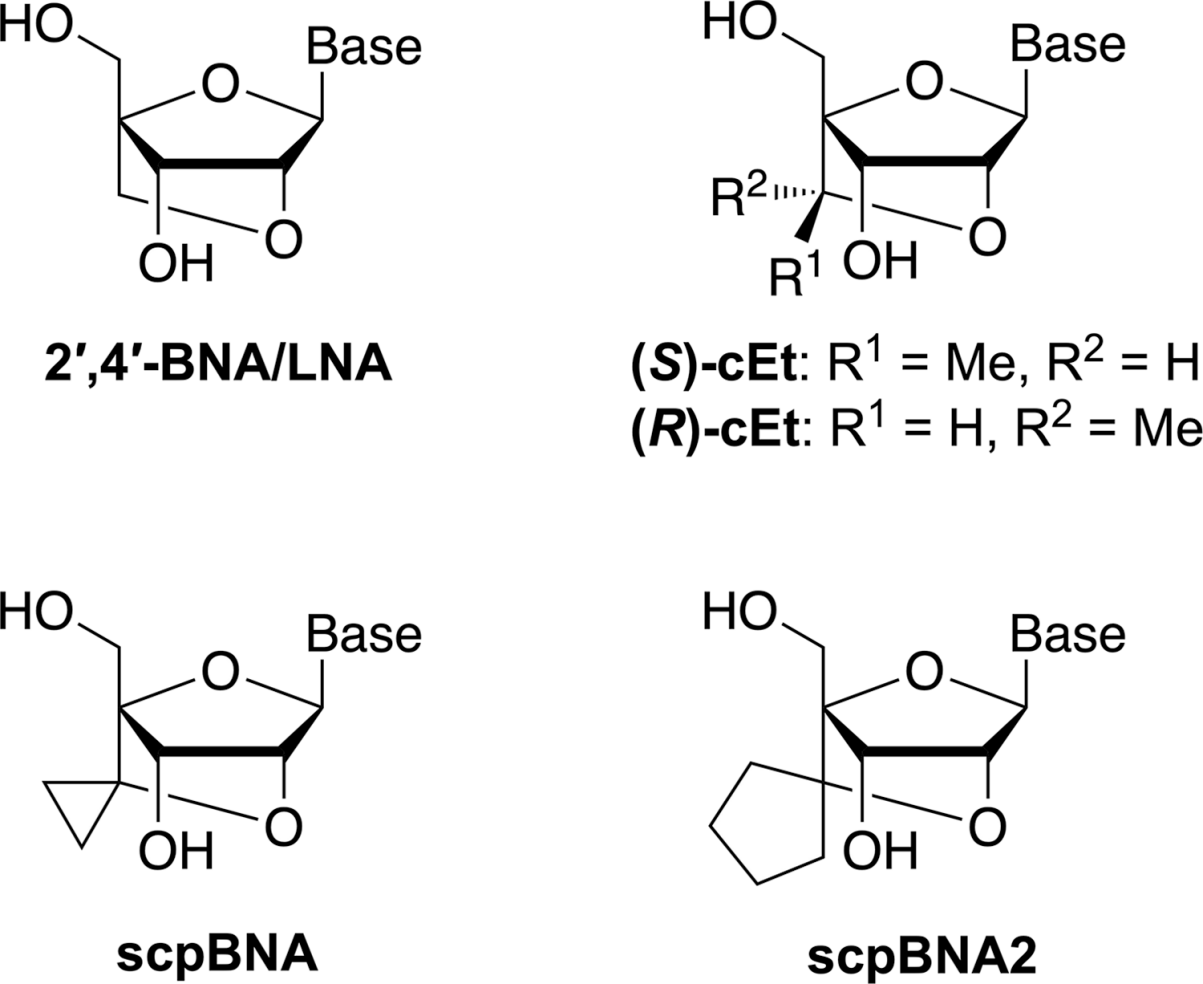
Nucleoside structures of 2′,4′-BNA/LNA, cEt, scpBNA, and scpBNA2. The base represents the nucleobase.

In this study, we present the synthesis and evaluation of 2′-*O*,4′-*C*-spirocyclopentylene-bridged nucleic acid (scpBNA2; Figure 1) as a novel modification for the wing region of gapmer-type ASOs. The synthesis of scpBNA2 phosphoramidites containing either thymine (T) or 5-methylcytosine (^m^C) nucleobases was achieved through geminal diallylation at the 2′,4′-bridge, followed by ring-closing metathesis. Oligonucleotides modified with scpBNA2 demonstrated a strong ability to form duplexes with complementary single-stranded RNA (ssRNA) and exhibited improved nuclease stability compared to those modified with scpBNA. Gapmer-type ASOs containing scpBNA or scpBNA2 induced target RNA knockdown both *in vitro* and *in vivo*, even with a partial reduction in PS modifications nearby. Conversely, reducing the number of PS modifications in 2′,4′-BNA/LNA-modified ASOs significantly diminished their activity, indicating that scpBNA and scpBNA2 facilitate a reduction in PS modifications. Furthermore, substitution of 2′,4′-BNA/LNA with scpBNA or scpBNA2 substantially reduced the hepatotoxicity observed with the parent ASOs. These findings suggest that scpBNA and scpBNA2 represent promising modifications for the development of safe and effective ASO drugs with an improved therapeutic window.

## Results and Discussion

### Synthesis of scpBNA2-T and scpBNA2-^m^C Phosphoramidites

scpBNA2-thymine (scpBNA2-T) phosphoramidite was prepared from compound **1**,^15^ a synthetic intermediate of scpBNA phosphoramidites (Scheme 1). By introducing two allyl groups into the methyl ester moiety of **1**, compound **2** was obtained in 98% yield. Compound **2** was converted to cyclopentenol derivative **3** via a ring-closing metathesis reaction using a Grubbs second generation catalyst. After silylation of the tertiary alcohol with TBSOTf, acetolysis of the 1,2-isopropylidene group afforded compound **5** (a mixture of 1:1 stereoisomers). The installation of a thymine nucleobase in a stereoselective fashion was accomplished by the Vorbrüggen reaction, and thymidine analog **6** was obtained in 89% yield from **5**. The 2′-*O*-acetyl group was then removed, and the resulting hydroxy group was mesylated to afford **8**. Similar to the preparation of the scpBNA-T nucleoside,^15^ compound **8** was converted into the bridged nucleoside **9** by treatment with tetrabutylammonium fluoride (TBAF) in THF, followed by treatment with K_2_CO_3_ in DMF. After hydrogenation of the olefin and hydrogenolysis of the two benzyl groups of **9**, the 5′-hydroxy group was dimethoxytritylated and the remaining 3′-hydroxy group was phosphitylated to afford scpBNA2-T phosphoramidite **12**. scpBNA2-T phosphoramidite **12** was obtained with a total yield of 27% (12 steps from **1**), which was higher than the total yield of 21% (11 steps from **1**) for scpBNA-T phosphoramidite.^15^

**Scheme 1:**
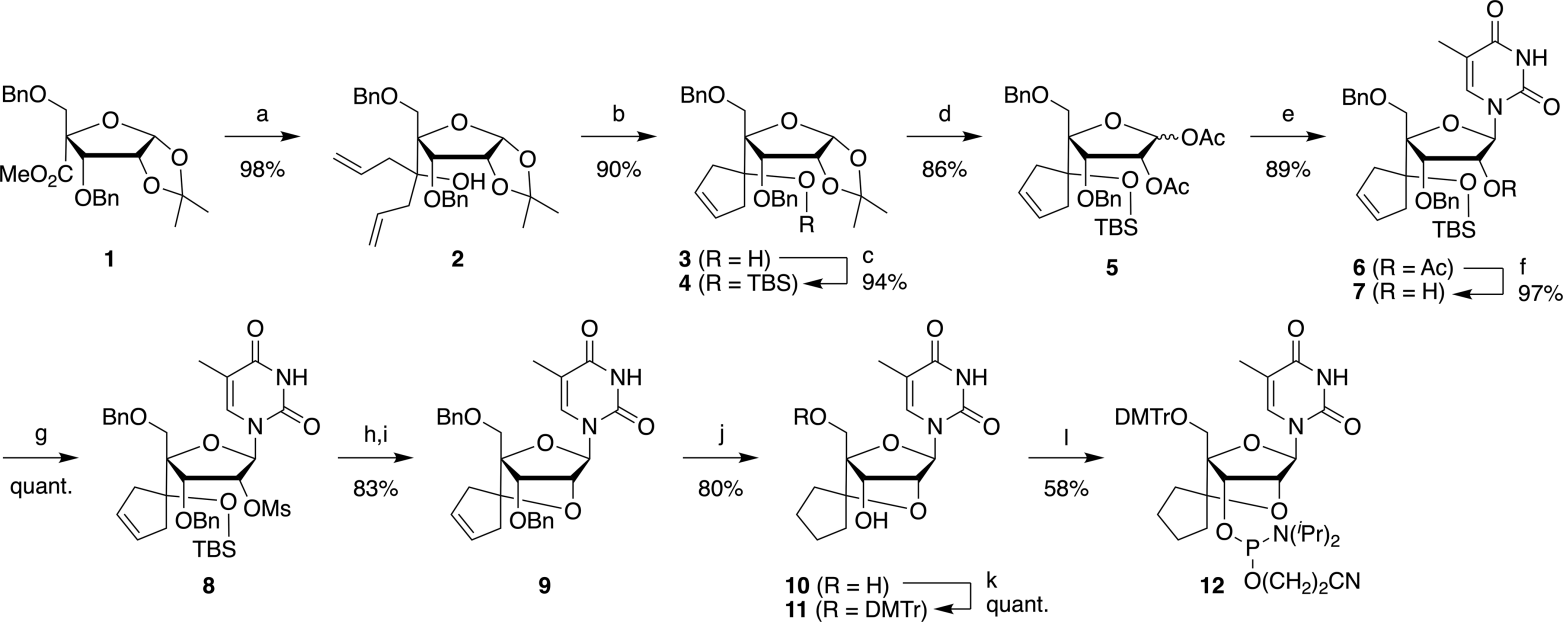
Synthesis of scpBNA2-T Phosphoramidite **12^a^**

a Reagents and conditions: (a) AllylMgBr, CeCl_3_, THF, rt, 14 h, 98%; (b) Grubbs 2nd generation catalyst, CH_2_Cl_2_, rt, 4 h, 90%; (c) TBSOTf, 2,6-lutidine, CH_2_Cl_2_, rt, 20 h, 94%; (d) TFA, Ac_2_O, AcOH, rt, 2 h, 86%; (e) thymine, TMSOTf, *N*,*O*-bis­(trimethylsilyl)­acetamide, MeCN, reflux, 5 h, 89%; (f) K_2_CO_3_, MeOH, rt, 5 h, 97%; (g) MsCl, pyridine, rt, 4 h, quant.; (h) TBAF, THF, rt, 30 h; (i) K_2_CO_3_, DMF, 90 °C, 20 h, 83% over two steps; (j) H_2_, Pd­(OH)_2_/C, AcOEt, rt, 0.5 h, 80%; (k) DMTrCl, pyridine, rt, 8 h, quant.; (l) 2-cyanoethyl-*N,N*-diisopropylchlorophosphoramidite, DIPEA, CH_2_Cl_2_, rt, 8 h, 58%.

scpBNA2 phosphoramidite bearing a 5-methylcytosine (^m^C) nucleobase was synthesized from the common intermediate **11** (Scheme 2). After triethylsilyl (TES) protection of the 3′–OH, the thymine nucleobase of **13** was converted into 5-methylcytosine. The exocyclic amino group of **14** was protected using BzCl, and the TES group was removed using TBAF to yield compound **16**. Finally, compound **16** was phosphitylated to produce scpBNA2-^m^C phosphoramidite **17**. The yield of scpBNA2-^m^C phosphoramidite **17** obtained from **1** over 16 steps was 18%, which was higher than the 11% yield obtained for scpBNA-^m^C phosphoramidite (15 steps from **1**).

**Scheme 2:**
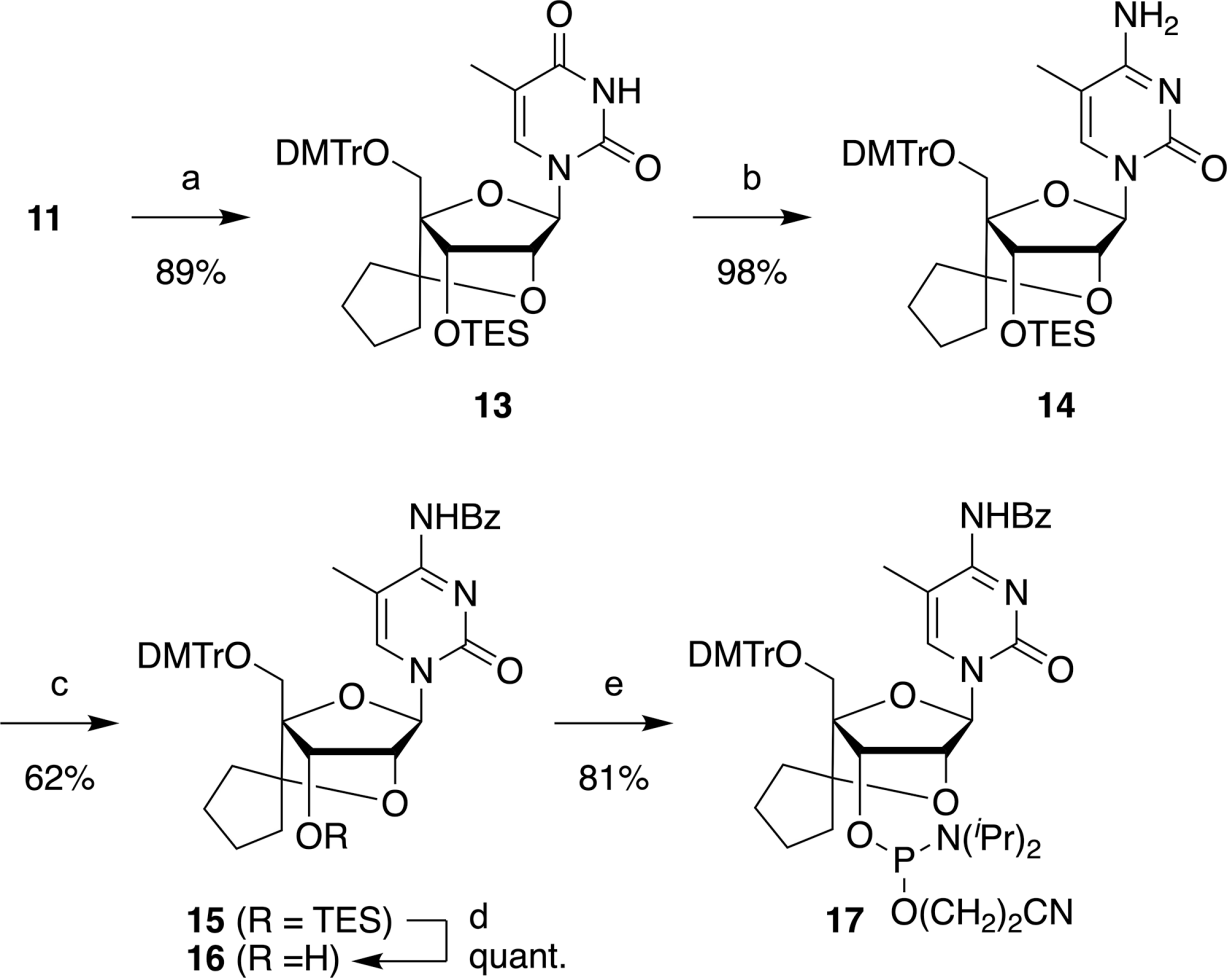
Synthesis of scpBNA2-^m^C Phosphoramidite **17^a^**

a Reagents and conditions: (a) TESCl, pyridine, rt, 7 h, 89%; (b) 1,2,4-triazole, POCl_3_, TEA, MeCN, rt, 0 °C to 1 h; sat. aq. NH3, 1,4-dioxane, rt, 1.5 h, 98%; (c) BzCl, pyridine, 0 °C to rt, 2.5 h, 62%; (d) TBAF, THF, 0 °C to rt, 20 min, quant.; (e) 2-cyanoethyl-*N*,*N*,*N*′,*N*′-tetraisopropylphosphorodiamidite, DCI, MeCN, 0 °C to rt, 2 h, 81%.

### Synthesis of scpBNA2-T-Modified Oligonucleotides

Using scpBNA2-T phosphoramidite **12**, we synthesized scpBNA2-T-modified oligonucleotides (**ON1**–**ON5**) using the standard phosphoramidite method (Table 1). A solution of 5-[3,5-bis­(trifluoromethyl)-phenyl]-1H-tetrazole (0.25 M in MeCN) was used as an activator, and the coupling time for incorporating scpBNA2-T phosphoramidite into the oligonucleotide was extended from 40 s to 12.5 min, following the coupling conditions for scpBNA phosphoramidites.^15,16^ As a result, the trityl monitor indicated good coupling efficacy for scpBNA2-T phosphoramidite (over 92% in all cases), and the scpBNA2-T-modified **ON1**–**ON5** were obtained in isolated yields of 17%–50% after cleavage from the solid support and purification by reversed-phase high-performance liquid chromatography (RP-HPLC). Matrix-assisted laser desorption/ionization-time-of-flight (MALDI-TOF) mass spectrometry data supported these structures.

**Table 1:** Isolated Yields and MALDI-TOF Mass Data of the scpBNA2-T-Modified Oligonucleotides

			MALDI-TOF mass	
ID	sequence (5′-3′)^a^	yield (%)	calcd [M–H]^−^	found [M–H]^−^
**ON1**	GCGTT **X** TTTGCT	36	3714.5	3714.6
**ON2**	GCG **X** T **X** T **X** TGCT	50	3878.7	3879.4
**ON3**	GCGT **XXX** TTGCT	29	3878.7	3879.1
**ON4**	GCG **XXXXXX** GCT	17	4125.0	4125.2
**ON5**	TTTTTTTTT **X**	19	3061.1	3060.5

a **X** = scpBNA2-T; G, C, and *T* = DNA. All linkages are phosphodiesters (PO).

### Thermal Stability of Duplexes Formed by scpBNA2-T-Modified Oligonucleotides

We then evaluated the thermal stability of the duplexes formed by scpBNA2-T-modified oligonucleotides (**ON1**–**ON4**) with complementary single-stranded RNA (ssRNA) or DNA (ssDNA) using UV-melting experiments. Unmodified oligonucleotide (**ON6**) and 2′,4′-BNA/LNA-T-modified oligonucleotides (**ON7**–**ON10**) were included as controls. The melting temperatures (*T*_m_) are listed in Table 2. Compared with natural **ON6**, the scpBNA2-T-modified oligonucleotides exhibited increased *T*_m_ values (Δ*T*_m_/mod. of +5.0 to +6.0 °C) toward ssRNA, which are comparable to those of the 2′,4′-BNA/LNA-T-modified oligonucleotides. However, scpBNA2-T-modified oligonucleotides displayed slightly lower *T*_m_ values toward ssDNA than their 2′,4′-BNA/LNA-modified counterparts, depending on the number of modifications. A similar trend was previously observed with scpBNA-T-modified oligonucleotides.^15^ We hypothesize that the additional cycloalkanes at the bridge of scpBNA and scpBNA2 introduce steric perturbations in the narrow minor groove of the DNA/DNA (B-form) duplexes. In contrast, for DNA/RNA (A-form) duplexes, cycloalkanes are positioned outside the duplex, resulting in no reduction in duplex-forming ability compared to 2′,4′-BNA/LNA.

**Table 2:** *T*_m_ Values (°C) of Duplexes Formed by Oligonucleotides with Complementary ssRNA or ssDNA^a^

		toward ssRNA		toward ssDNA	
ID	sequence (5′-3′)^b^	*T*_m_ (°C)	Δ*T*_m_/mod. (°C)	*T*_m_ (°C)	Δ*T*_m_/mod. (°C)
**ON6**	GCGTTTTTTGCT	47	–	51	–
**ON1**	GCGTT **X** TTTGCT	53	+6.0	52	+1.0
**ON7**	GCGTT **Y** TTTGCT	52	+5.0	52	+1.0
**ON2**	GCG **X** T **X** T **X** TGCT	63	+5.3	55	+1.3
**ON8**	GCG **Y** T **Y** T **Y** TGCT	63	+5.3	56	+1.7
**ON3**	GCGT **XXX** TTGCT	62	+5.0	53	+0.7
**ON9**	GCGT **YYY** TTGCT	62	+5.0	55	+1.3
**ON4**	GCG **XXXXXX** GCT	79	+5.3	62	+1.8
**ON10**	GCG **YYYYYY** GCT	81	+5.7	68	+2.8

a Conditions: 10 mM phosphate buffer (pH 7.2), 100 mM NaCl, and 4 μM of each oligonucleotide. Monitored at 260 nm (0.5 °C/min). *T*_m_ values reflect the average of three measurements. The sequences of ssRNA and ssDNA are 5′-r­(AGCAAAAAACGC)-3′ and 5′-d­(AGCAAAAAACGC)-3′, respectively. Δ*T*_m_/mod. represents the change in *T*_m_ value (Δ*T*_m_) per modification compared to the unmodified standard strand (**ON6**).

b **X** = scpBNA2-T; **Y** = 2′,4′-BNA/LNA-T; G, C, and *T* = DNA. All linkages are phosphodiesters (PO).

### 3′-Exonuclease Stability of scpBNA2-T-Modified Oligonucleotides

We assessed the stability of the scpBNA2-T-modified oligonucleotide against 3′-exonuclease activity by using snake venom phosphodiesterase (svPDE). Poly-T oligonucleotides, each containing either 5′-phosphorothioate-modified thymidine (5′-PS-modified T; a 1:1 mixture of *S*_p_ and *R*_p_ isomers), scpBNA-T, or scpBNA2-T at the 3′-terminous, were incubated with 1.5 μg/mL svPDE. The time-dependent degradation of these oligonucleotides was monitored using HPLC and plotted against incubation time (Figure 2, see also Figure S1). After 40 min, 31% of the scpBNA-T-modified oligonucleotide (**ON12**) retained its integrity, whereas 38% of the PS-modified oligonucleotide (**ON11**) remained intact. The scpBNA2-T-modified oligonucleotide (**ON5**) exhibited greater stability, with 71% of it remaining intact after the same incubation period. This remarkable stability is attributed to the cyclopentane moiety of scpBNA2, which sterically blocks nuclease access to the 3′-end phosphodiester (PO) bond. Notably, our previous studies showed that the scpBNA-T modification conferred 40-fold greater stability against svPDE compared to the 2′,4′-BNA/LNA-modified counterpart.^15^ The introduction of the scpBNA2 modification further enhanced this stability by more than 4-fold relative to scpBNA. Collectively, these modifications provide significant advantages in boosting nuclease resistance.

**Figure 2:**
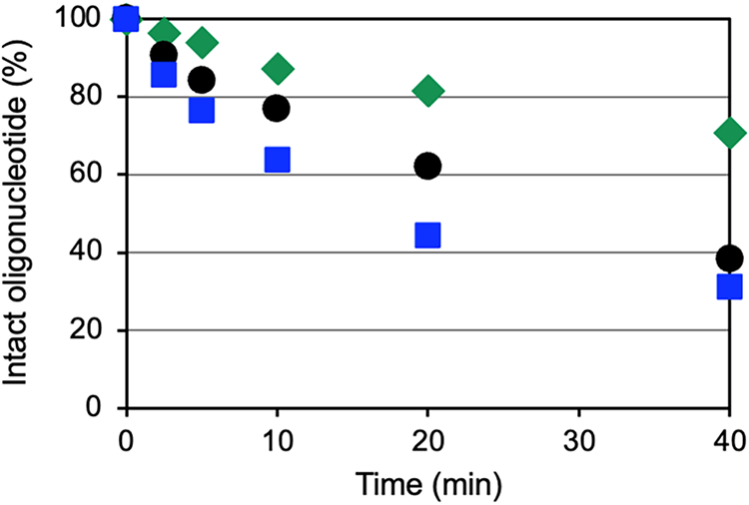
Stability of 5′-d­(TTTTTTTTT **X**)-3′ against svPDE. **X** = scpBNA2-T (green diamonds, **ON5**), 5′-PS-modified thymidine (black circles, **ON11**), or scpBNA-T (blue squares, **ON12**). Conditions: 50 mM Tris-HCl (pH 8.0), 10 mM MgCl_2_, 7.5 μM of each oligonucleotide, and 1.5 μg/mL svPDE at 37 °C.

### *In Vitro* Antisense Activity of scpBNA2-Modified ASOs

To investigate the activity of scpBNA2-modified ASOs, **ON19**–**ON21** targeted mouse *Malat1* RNA^18^ was synthesized using phosphoramidites **12** and **17** (Table 3, see also Table S1 for the obtained yields for **ON19**–**ON21**). For comparison, 2′,4′-BNA/LNA- and scpBNA-modified counterparts (**ON13**–**ON18**) were prepared and evaluated. It was hypothesized that reducing the number of PS modifications in 2′,4′-BNA/LNA-modified ASOs leads to decreased activity, whereas nuclease-resistant scpBNA- and scpBNA2-modified ASOs would retain their activity even with fewer PS modifications. The ASOs were tested in mouse hepatocyte-derived NMuLi cells. Cells were exposed to either 30 or 100 μM ASO under gymnotic conditions (without transfection reagents) and incubated for 48 h. Postincubation, the expression levels of the target *Malat1* RNA were measured using quantitative reverse transcription-polymerase chain reaction (qRT-PCR). The results are shown in Figure 3. The results revealed that 2′,4′-BNA/LNA gapmers with partial PS modifications (**ON14** and **ON15**) showed lower activity than the fully PS-modified **ON13**. In contrast, scpBNA- and scpBNA2-modified ASOs maintained their activity even when PS modifications were reduced. Interestingly, although no significant difference in activity was observed between **ON19** and **ON21**, the fully PS-modified **ON19** displayed slightly lower activity than the partially PS-modified **ON21** and **ON20**. According to the RP-HPLC results for **ON13**–**ON21** (Figure S2), scpBNA2-modified ASOs demonstrated higher hydrophobicity than those modified with scpBNA and 2′,4′-BNA/LNA. The most hydrophobic ASO, fully PS-modified **ON19**, may interact more strongly with proteins (e.g., fetal bovine serum proteins or cell membrane proteins), potentially reducing the effective concentration of ASOs available to reach the target RNA.^19,20^ All ASOs demonstrated a similar duplex-forming ability toward complementary ssRNA (Table S2).

**Table 3:**
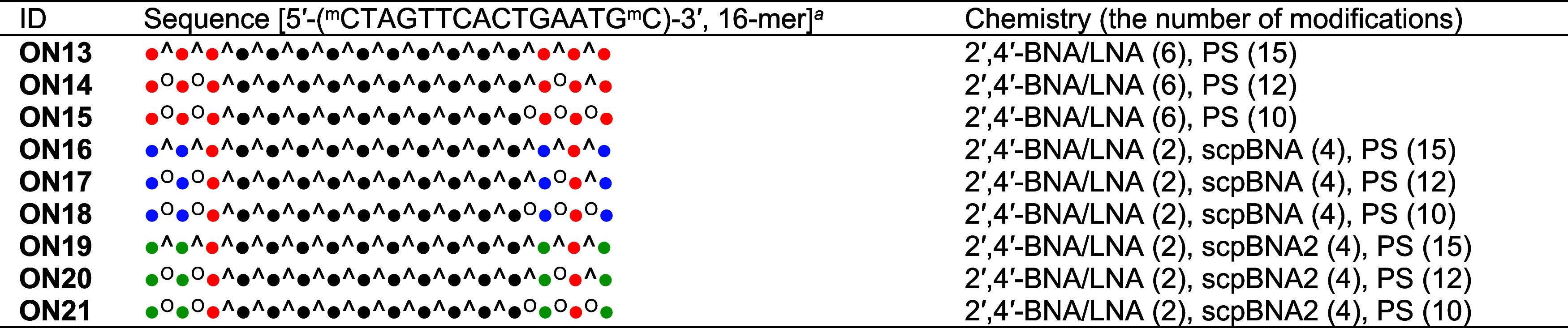
Chemistries Used in ASOs Targeting *Malat1*

a Marks “^∧^” and “^O^” indicate phosphorothioate (PS) and phosphodiester (PO) linkages, respectively. Red, black, blue, and green circles indicate 2′,4′-BNA/LNA, DNA, scpBNA, and scpBNA2, respectively. ^m^C represents 5-methylcytosine.

**Figure 3:**
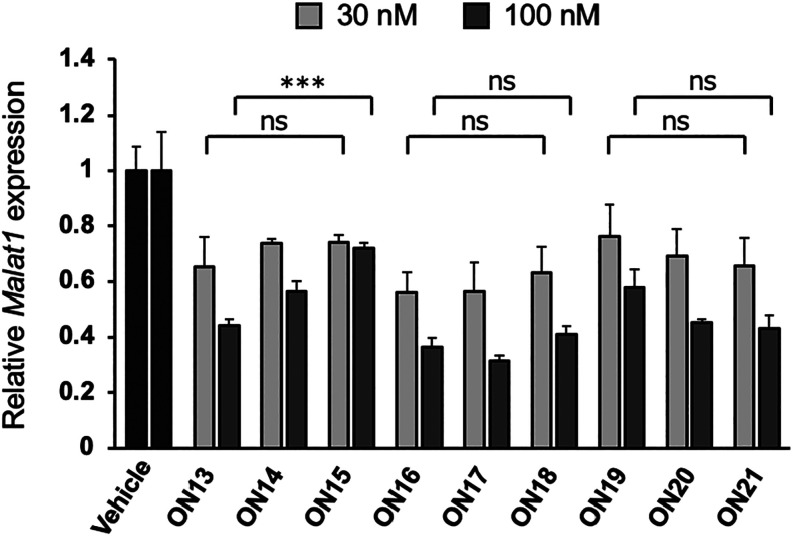
qRT-PCR analysis of the relative *Malat1* RNA expression levels in NMuLi cells after gymnotic treatment with ASOs (30 or 100 μM) for 48 h. This experiment was conducted in triplicate (*n* = 3 per group). Data are presented as mean ± SD. Statistical significance was established using the Tukey’s multiple comparison test. *** *P* < 0.001; ns, not significant.

Next, we synthesized **ON25**–**ON29** (Table S3), which featured different modification patterns than **ON14**–**ON21**, while retaining the same sequence as the ASOs in Table 3. Consistent with the results shown in Figure 3, **ON28**, the most hydrophobic scpBNA2-modified ASO, demonstrated lower activity, whereas its PS-reduced counterpart (**ON29**) exhibited activity comparable to **ON13** (Figure S3; see Figure S4 for HPLC retention behavior, and Table S4 for the duplex-forming ability of these ASOs). In contrast, reducing the number of PS modifications in the 2′,4′-BNA/LNA gapmers resulted in a significant loss of activity (**ON13** vs **ON25**). Remarkably, only scpBNA2-modified ASOs showed improved activity upon reducing PS modifications.

### *In Vivo* Antisense Activity of scpBNA2-Modified ASOs

Although *in vitro* evaluations, such as those conducted under gymnotic conditions or with Ca^2+^ enrichment,^21,22^ offer valuable insights into potential *in vivo* activity, it is important to recognize that *in vitro* results do not always directly correlate with *in vivo* efficacy because of differences in trafficking mechanisms and nuclease activity. Therefore, we extended our study to investigate the *in vivo* antisense activity of **ON13**–**ON21**. To assess *in vivo* knockdown activity, 6-week-old mice were intravenously administered a single dose of 20 nmol (approximately 0.1 mg) of ASO via the tail vein. Liver samples were collected 72 h after administration, and *Malat1* RNA expression levels were quantified (Figure 4). Similar to the *in vitro* results, 2′,4′-BNA/LNA-modified ASOs exhibited reduced activity in the liver when PS modifications were decreased. In contrast, scpBNA- and scpBNA2-modified ASOs maintained their activity, even with reduced PS modifications. These findings underscore the ability of both scpBNA and scpBNA2 to retain their *in vivo* activity despite a reduction in PS content. Reducing PS modifications in gapmer ASOs is currently an attractive strategy to mitigate acute neurotoxicity during intracerebroventricular administration,^23^ even though it can sometimes compromise target knockdown activity. Our results suggest that scpBNA and scpBNA2 may offer a way to enhance the safety of ASOs targeting the nervous system without this trade-off.

**Figure 4:**
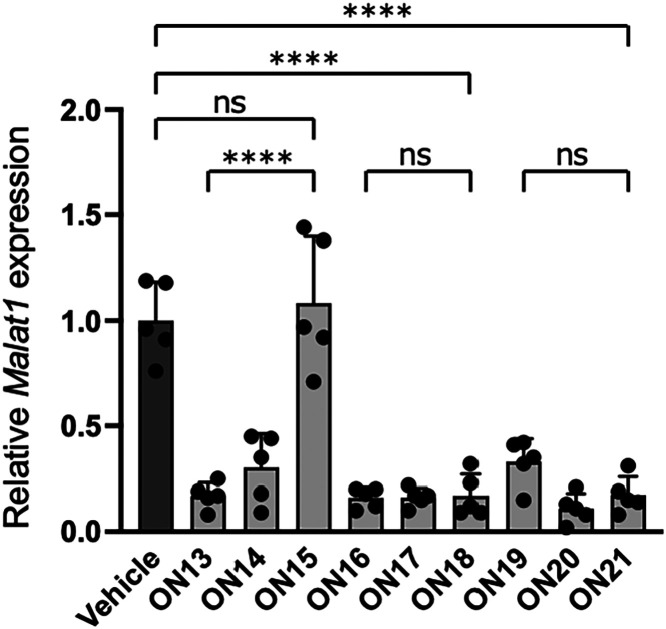
qRT-PCR analysis of relative *Malat1* RNA expression levels after the administration of ASOs. BALB/cAnNCrlCrlj mice were intravenously injected with 20 nmol of ASOs. After 72 h, mice livers were harvested, and the expression levels of *Malat1* RNA were quantified. Data are presented as mean ± SD (*n* = 5 per group). Statistical significance was established using Tukey’s multiple comparison test. **** *P* < 0.0001; ns, not significant.

### Reduction of Hepatotoxicity by scpBNA Series

We further investigated the potential of scpBNA and scpBNA2 in gapmer ASO design to mitigate hepatotoxicity. Hepatotoxicity associated with gapmer ASOs, independent of off-target RNA reductions, is believed to arise from interactions between ASOs and cellular proteins, particularly those in hepatocytes.^5,6,24,25^ We hypothesized that the additional cycloalkane moiety in scpBNA and scpBNA2 could alter or reduce these interactions, thereby lowering hepatotoxicity. Previous studies have shown that even minor chemical modifications can significantly impact the risk of hepatotoxicity, likely by modifying ASO-protein interactions.^26–33^ To test this hypothesis, we selected a 2′,4′-BNA/LNA-modified gapmer-type ASO (**ON22**, Table 4) which we screened and identified as an ASO that induces severe hepatotoxicity.^32^ We then synthesized **ON23** and **ON24** by replacing 2′,4′-BNA/LNA-T in the wing regions of **ON22** with scpBNA-T and scpBNA2-T, respectively (details of **ON24** yield are provided in Table S5). Mice (*n* = 5 per group) were administrated a single intraperitoneal (*i.p.*) dose of 20 mg/kg of each oligonucleotide, and alanine aminotransferase (ALT) and aspartate aminotransferase (AST) levels in serum were measured 96 h postadministration. All the mice injected with **ON22** died within 96 h. In contrast, mice treated with **ON23** or **ON24** survived without any observable abnormalities, allowing for blood sample collection and subsequent evaluation of ALT and AST levels. As summarized in Table 4, scpBNA- and scpBNA2-modified oligonucleotides caused only minor increases in ALT and AST levels compared to the saline control group (ALT: 22.8 ± 1.6 U/L, AST: 73.8 ± 37.5 U/L). Moreover, no abnormal liver discoloration or enlargement was observed. These findings indicated that replacing 2′,4′-BNA/LNA with scpBNA or scpBNA2 effectively reduced hepatotoxicity. In our previous research, the chemical modification of **ON22** notably reduced hepatotoxicity without significant changes in off-target gene expression,^32^ suggesting that the hepatotoxicity of **ON22** is primarily driven by hybridization-independent pathways. Therefore, the reduced hepatotoxicity observed with **ON23** and **ON24** is likely attributed to the suppression of these hybridization-independent pathways, facilitated by the incorporation of cycloalkanes.

**Table 4:**
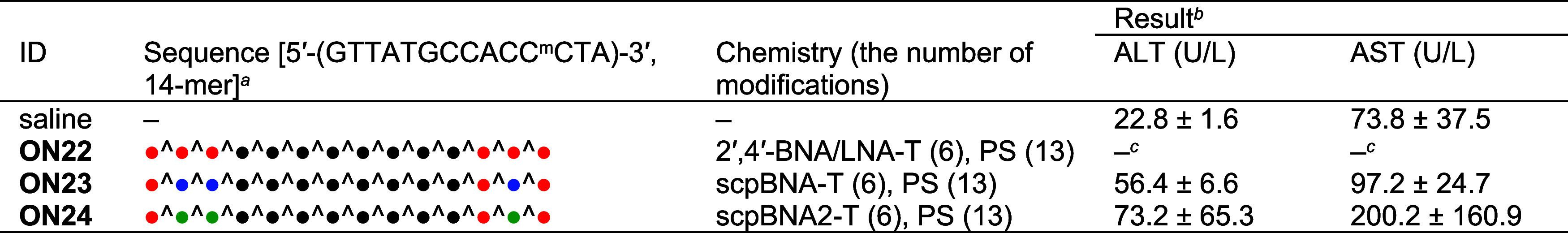
Relationship between Modifications and Hepatotoxicity (ALT/AST Data)

a Mark “^∧^” indicates phosphorothioate (PS) linkage. Red, black, blue, and green circles indicate LNA, DNA, scpBNA, and scpBNA2, respectively. ^m^C represents 5-methylcytosine.

b ALT and AST levels in mice 96 h after a single dose of 20 mg/kg ASOs. Data are presented as mean ± SD.

c All mice died.

We also evaluated another ASO sequence targeting *Nr3c1* (Table 5; see Table S6 for the yields of **ON34** and **ON35**). The parent 2′,4′-BNA/LNA-modified ASO, **ON30**, is known for its hepatotoxicity.^32–36^ In this study, mice (*n* = 5 per group) received a single intravenous dose of 100 nmol ASO (approximately 0.5 mg). Target RNA knockdown activity in the liver, along with serum ALT and AST levels, was assessed 96 h postadministration (Figure 5). Consistent with previous reports, **ON30** effectively reduced liver *Nr3c1* expression but caused significant hepatotoxicity, as evidenced by elevated ALT and AST levels and reduced body weight (Figure S5). In contrast, its scpBNA- and scpBNA/scpBNA2-modified counterparts (**ON32** and **ON34**, respectively) demonstrated similar knockdown activity to **ON30** but did not cause any increases in ALT or AST levels. The primary structural difference among these ASOs is the presence or absence of cycloalkanes in the wing regions, which minimally impact hybridization ability. These findings suggest that the hepatotoxicity associated with **ON30** is primarily driven by a hybridization-independent mechanism, as observed with **ON22**, and that this toxicity can be mitigated by incorporating cycloalkanes. Meanwhile, **ON31**, a 2′,4′-BNA/LNA-modified ASO with six PO linkages, showed neither RNA knockdown activity nor toxicity. ALT and AST levels, as well as body weight, remained comparable to those in the saline control group. This lack of activity and toxicity may be due to its rapid renal excretion facilitated by the reduced PS modifications, i.e., the six PO linkages in **ON31** likely promote faster degradation, generating shorter ASO metabolites with reduced plasma protein-binding ability,^37^ which are quickly excreted in urine. The six PO linkages in **ON31** may also reduce the internalization of **ON31** into hepatocytes. Among the three ASOs containing six PO linkages, scpBNA-modified **ON33** and scpBNA/scpBNA2-modified **ON35** demonstrated *Nr3c1* knockdown activity, though at lower levels than their fully PS-modified counterparts (**ON32** and **ON34**). This contrasts with the findings in Figure 4, where **ON18** and **ON21** exhibited comparable activity to **ON16** and **ON19**. The difference likely arises from the shorter length and higher PO linkage content of **ON33** and **ON35**. Notably, **ON35** exhibited higher activity than **ON33**, likely due to its greater nuclease stability. Importantly, both **ON33** and **ON35** showed no signs of toxicity. This absence of toxicity is likely attributed to several factors, including lower hepatocyte distribution and rapid degradation caused by the reduction the number of PS modifications, as well as reduced or altered interactions with toxicity-related proteins via cycloalkane incorporation. While further studies are required to elucidate the underlying mechanisms and validate the broader applicability of these findings, our data highlight that substituting 2′,4′-BNA/LNA with scpBNA or scpBNA2 can significantly enhance the safety profile of gapmer-type ASOs. Moreover, a recent independent study—using a different ASO sequence—similarly reported reduced hepatotoxicity when 2′,4′-BNA/LNA was replaced with scpBNA,^38^ further supporting the translational potential of this strategy.

**Table 5:**
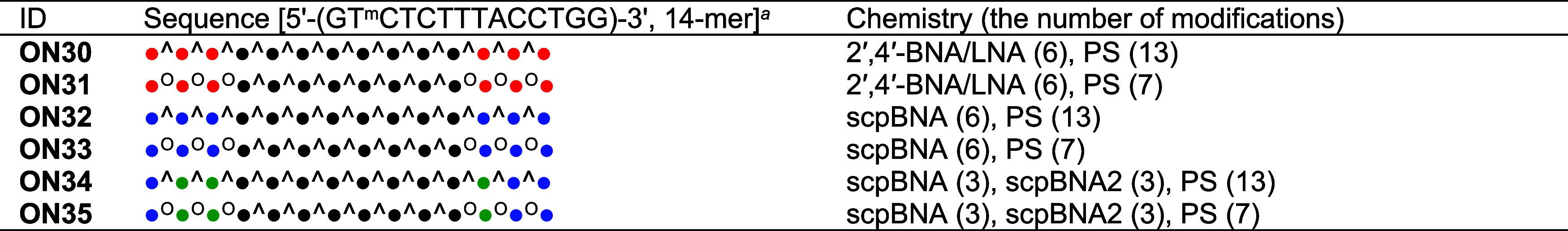
Chemistries Used in ASOs Targeting *Nr3c1*

a Marks “^∧^” and “^O^” indicate phosphorothioate (PS) and phosphodiester (PO) linkages, respectively. Red, black, blue, and green circles indicate 2′,4′-BNA/LNA, DNA, scpBNA, and scpBNA2, respectively.

**Figure 5:**
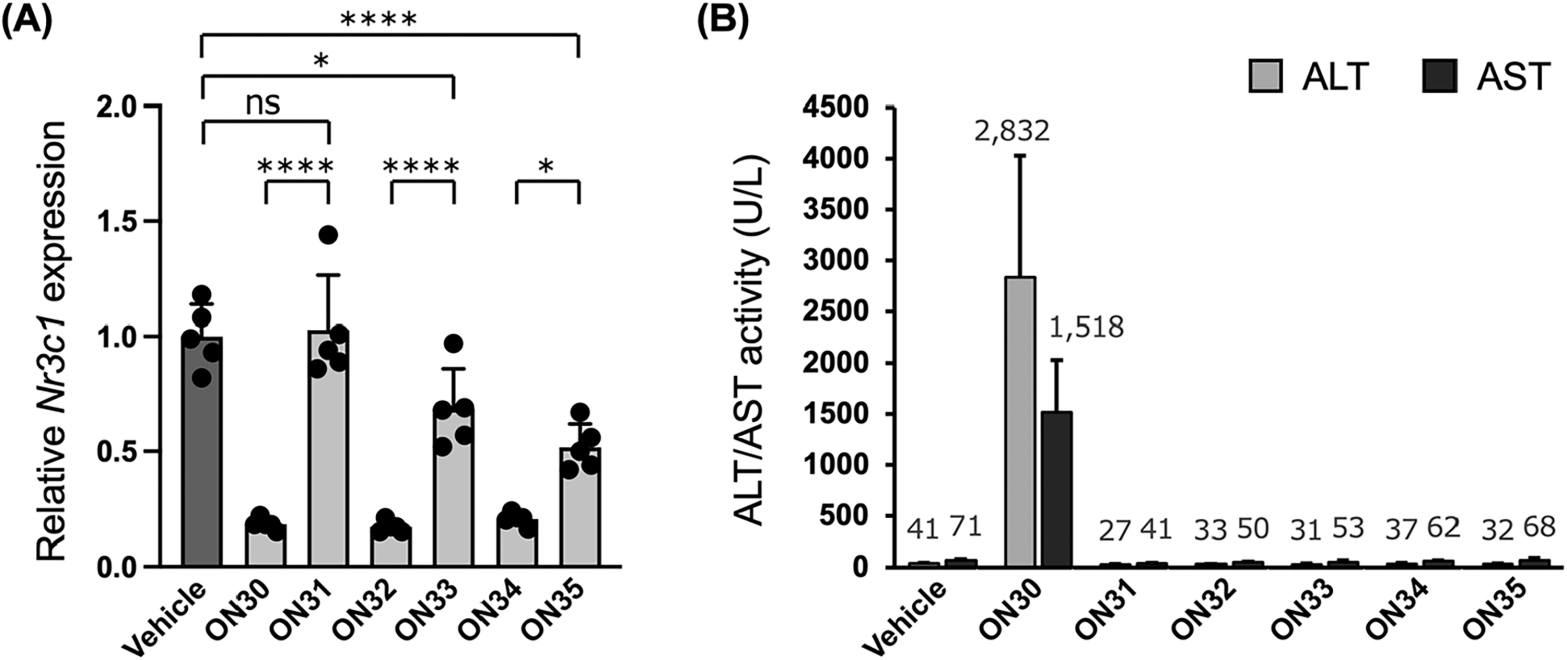
Target knockdown activity and hepatotoxicity after administration of ASOs. (A) qRT-PCR analysis of relative *Nr3c1* RNA expression levels after the administration of ASOs. C57BL/6NCrl mice were intravenously injected with 100 nmol of ASOs. After 96 h, mice livers were harvested, and the expression levels of *Nr3c1* RNA were quantified. Data are shown as mean ± SD (*n* = 5 per group). Statistical significance was established using Tukey’s multiple comparison test. * *P* < 0.05; **** *P* < 0.0001; ns, not significant. (B) ALT and AST levels at 96 h postadministration.

## Conclusion

In this study, scpBNA2 phosphoramidites bearing T or ^m^C nucleobases were synthesized with good yields and successfully incorporated into oligonucleotides. These modified oligonucleotides were evaluated for their duplex-forming ability with complementary ssRNA and their stability against 3′-exonuclease activity. The scpBNA2-modified oligonucleotides showed robust duplex-forming capabilities comparable to those of 2′,4′-BNA/LNA- and scpBNA-modified oligonucleotides. Notably, they exhibited greater nuclease stability than their 2′,4′-BNA/LNA-modified oligonucleotides and surpassed the stability of scpBNA-modified oligonucleotides. Gapmer-type ASOs incorporating scpBNA or scpBNA2 achieved RNA knockdown activity equivalent to that of ASOs containing 2′,4′-BNA/LNA. Remarkably, the RNA knockdown activity of scpBNA- or scpBNA2-modified ASOs was maintained even when the number of PS modifications was partially reduced, a feature likely attributed to their enhanced nuclease stability—a property not observed with 2′,4′-BNA/LNA-modified ASOs. Furthermore, substituting 2′,4′-BNA/LNA with scpBNA or scpBNA2 in gapmer ASO design significantly reduced hepatotoxicity. These findings highlight the considerable potential of scpBNA and scpBNA2 for developing safer antisense oligonucleotides.

## Material and Methods

### Synthesis of scpBNA2 Phosphoramidites

All moisture-sensitive reactions were performed in well-dried glassware under N_2_ or Ar atmosphere. Anhydrous acetonitrile (MeCN), dichloromethane (CH_2_Cl_2_), *N,N*-dimethylformamide (DMF), pyridine, and tetrahydrofuran (THF) were used as received. ^1^H, ^13^C, and ^31^P NMR spectra were recorded using JNM-AL300, JNM-ECS400, and JNM-ECA500 spectrometers (JEOL Ltd.). Chemical shift values are expressed in δ values (ppm) relative to tetramethylsilane (0.00 ppm) as the internal standard, residual CHCl_3_ (7.26 ppm) or CH_3_OH (3.31 ppm) for ^1^H NMR, and tetramethylsilane (0.00 ppm), chloroform-*d*_1_ (77.00 ppm), or methanol-*d*_4_ (49.00 ppm) for ^13^C NMR. For ^31^P NMR, 5% H_3_PO_4_ (0.00 ppm) was used as the external standard. The mass spectra of all the new compounds were recorded using a MALDI-TOF mass spectrometer (SpiralTOF JMS-S3000, JEOL Ltd.). Flash column chromatography was performed using an EPCLC-W-Prep 2XY column (YAMAZEN Co.). The synthetic procedure for the scpBNA2 phosphoramidites is provided in the Supporting Information.

### Synthesis, Purification, and Characterization of scpBNA2-Modified Oligonucleotides

Oligonucleotides modified with scpBNA2 were synthesized using an nS-8 oligonucleotide synthesizer (GeneDesign Inc.). The coupling time of the scpBNA2 phosphoramidites increased from 40 s to 12.5 min. 5-[3,5-Bis­(trifluoromethyl)-phenyl]-1*H*-tetrazole (Activator 42, 0.25 M in MeCN) was used as the activator. The other synthetic procedures followed the standard phosphoramidite protocol. Cleavage from the solid support and removal of all protecting groups were performed using saturated aq. NH_4_OH for 1.5 h at room temperature, and then overnight at 55 °C. The resulting DMTr-on oligonucleotides were briefly purified using a Sep-Pak Plus C18 cartridge, and the DMTr group was removed using 2% aq. trifluoroacetic acid in the cartridge. Oligonucleotides were further purified using RP-HPLC (Waters XTerra MS C18 2.5 μm, 10 × 50 mm column). The purity of the purified oligonucleotides was analyzed by reverse-phase HPLC (Waters XTerra MS C18 2.5 μm, 4.6 × 50 mm column), and their compositions were confirmed using MALDI-TOF mass analysis (JEOL SpiralTOF JMS-S3000, or Bruker Daltonics Autoflex maX TOF/TOF). For HPLC analysis, Shimadzu DGU-20A_3R_, LC-20AD, CBM-20A, CTO-20AC, SPD-20A, and FRC-10A instruments were used. The absorbance at 260 nm was measured using Shimadzu UV-1800 and DeNovix DS-11 spectrometers to determine the oligonucleotide concentration.

### UV-Melting Experiments

Melting temperatures (*T*_m_) were determined using Shimadzu UV–vis–UV-1650PC and UV-1800PC spectrophotometers equipped with a TMSPC-8 *T*_m_ analysis accessory. Equimolar amounts of the target single-stranded RNA (ssRNA) or DNA (ssDNA) and oligonucleotides were dissolved in 10 mM sodium phosphate buffer (pH 7.2) with 100 mM NaCl to achieve a final concentration of 4 μM for each strand. The samples were then heated to 100 °C and slowly cooled to room temperature for annealing. The absorbance at 260 nm was recorded at a scan rate of 0.5 °C/min from 5 to 90 °C. *T*_m_ values were determined from the temperatures at which half of the duplexes dissociated based on sigmoidal melting curves.

### Nuclease Resistance Study

Oligonucleotides (750 pmol) were dissolved in 50 mM Tris-HCl buffer (pH 8.0, 95 μL) containing 10 mM MgCl_2_. To each sample, 0.15 μg of phosphodiesterase I from *Crotalus adamanteus* venom (snake venom phosphodiesterase [svPDE]) in 5 μL of water was added, followed by incubation at 37 °C. Aliquots (10 μL) were taken at 0, 2.5, 5.0, 10, 20, and 40 min, heated at 90 °C to deactivate the enzyme, and analyzed using reverse-phase HPLC (Waters XTerra MS C18 2.5 μm, 4.6 × 50 mm column) to assess the intact oligonucleotide proportion. The HPLC data are shown in Figure S1.

### Cell-Based Assay

NMuLi cells were purchased from the American Type Culture Collection and cultured in Dulbecco’s modified Eagle’s medium with 4500 mg/L glucose, l-glutamine, sodium pyruvate, and sodium bicarbonate (Sigma-Aldrich), supplemented with 10% heat-inactivated fetal bovine serum (Thermo Fisher Scientific Inc.) and 1% antibiotic-antimycotic (Sigma-Aldrich) at 37 °C in a 5% CO_2_ incubator. Cells were seeded in 96-well plates (Iwaki) at a density of 5.0 × 10^3^ cells/well (*n* = 3/group) and treated with ASOs after 24 h to achieve a final concentration of 30 or 100 nM (gymnosis). After an additional 48 h, cDNA was prepared using the SuperPrep II Cell Lysis & RT Kit for qPCR (Toyobo Co., Ltd.), following the manufacturer’s instructions. qRT-PCR was performed using PowerTrack SYBR Green Master Mix (Thermo Fisher Scientific Inc.) and StepOnePlus Real-Time PCR System (Thermo Fisher Scientific Inc.). The expression of target genes was normalized to *Gapdh* expression levels. The primer sequences used in this study were as follows: *Malat1* forward: 5′-ACATTCCTTGAGGTCGGCAA-3′; reverse: 5′-CACCCGCAAAGGCCTACATA-3′; *Gapdh* forward: 5′-TCACCACCATGGAGAAGGC-3′; reverse: 5′-GCTAAGCAGTTGGTGGTGCA-3′. The primers were synthesized by Hokkaido System Science Co., Ltd.

### Animal Experiment

All animal experiments were performed in accordance with the animal welfare bylaws of The University of Osaka and LSIM Safety Institute Corporation (Ibaraki, Japan). All experimental procedures were reviewed and approved by the Institutional Animal Care and Use Committee.

The experimental procedures for the data in Figure 4 were as follows: Mice (BALB/cAnNCrlCrlj, female, 5 weeks old) were purchased from Charles River Laboratories Japan and housed in a ventilated animal room maintained between 19–25 °C under a 12/12 h light/dark cycle with food and water supplied *ad libitum*. After acclimation, mice (*n* = 5 per group) at 7 weeks of age were intravenously administered saline (200 μL) or 100 μM solution of ASO in saline (200 μL). Seventy-two h after the injection, the mice were anesthetized by isoflurane inhalation and euthanized by exsanguination after cutting the abdominal aorta. The liver (lateral left lobe) was collected and stored overnight in RNAlater (RNA Stabilization Reagent, QIAGEN K. K.) in a refrigerator (4 °C). RNAlater was discarded, and the tissue samples were stored at approximately −80 °C until RNA extraction. The tissue samples were homogenized using a μT-12 beads crusher (TAITEC Co.) with zirconia beads for 30 s, and total RNA was isolated using an RNeasy Mini Kit (QIAGEN K.K.), according to the manufacturer’s instructions. qRT-PCR was performed using the One-Step TB Green PrimeScript RT-PCR Kit (Takara Bio Inc.) and analyzed using a 7500 Real-Time PCR System (Applied Biosystems). Primer sequences were the same as those described for the cell-based assay. Primers were synthesized by Fasmac Co., Ltd.

The experimental procedures for the data in Table 4 were as follows: Mice (C57BL/6J, male, 6 weeks old, *n* = 5 per group) were intraperitoneally administered saline or ASO in saline at 20 mg/kg. Ninety-six h after injection, blood samples were collected from the abdominal aorta under anesthesia by isoflurane, and serum was used to analyze alanine aminotransferase (ALT) and aspartate aminotransferase (AST) levels. Serum ALT and AST levels were measured using an automated clinical analyzer (DRI-CHEM 4000 V; FUJIFILM).

Experimental procedures for the data in Figure 5 and Figure S5 are as follows: Mice (C57BL/6NCrl, male, 5 weeks old) were purchased from The Jackson Laboratory Japan and housed in a ventilated animal room maintained at 19–25 °C under a 12/12 h light/dark cycle with food and water supplied *ad libitum*. After acclimation, mice (*n* = 5 per group) at 6 weeks of age were intravenously administered saline (200 μL) or a 500 μM solution of ASO in saline (200 μL). Ninety-six h after the injection, approximately 0.5 mL of blood samples were collected from the posterior vena cava under anesthesia by inhalation of isoflurane. The mice were euthanized by exsanguination after cutting the abdominal aorta. Blood samples were allowed to stand for 30–60 min and centrifuged at 10,000*g* for 3 min to obtain the serum. Serum ALT and AST levels were measured using an automated clinical analyzer (TBA-2000FR; Canon Medical Systems Inc.). The liver (lateral left lobe) was collected, and RNA was isolated and analyzed using the same method described for Figure 4. The expression levels of the target genes were normalized to that of mouse *Gapdh*. The primer sequences used in this study were as follows: *Nr3c1* forward: 5′-ACTGTCCAGCATGCCGCTAT-3′; reverse: 5′-GCAGTGGCTTGCTGAATTCC-3′; *Gapdh* forward: 5′-TCACCACCATGGAGAAGGC-3′; reverse: 5′-GCTAAGCAGTTGGTGGTGCA-3′.

**ON22**–**ON24** were dosed intraperitoneally following the established protocol at The University of Osaka, whereas **ON13–ON21** and **ON30–ON35** were dosed intravenously (tail vein) at LSIM Safety Institute Corporation and the National Institute of Health Sciences according to the institutions’ standard protocols, and both routes provide systemic exposure.

## Supplementary Material



## Notes

All relevant data sets supporting the findings of this study, including the procedures for monomer synthesis and characterization data for the compounds (including ^1^H, ^13^C, and ^31^P NMR spectra for all synthesized monomers, as well as HPLC and MS data for the synthesized oligomers) (PDF)

## References

[1] Crooke S. T., Liang X. H., Baker B. F., Crooke R. M. (2021). Antisense technology:
A review. J. Biol. Chem..

[2] Crooke S. T., Baker B. F., Crooke R. M., Liang X. H. (2021). Antisense technology:
An overview and prospectus. Nat. Rev. Drug Discovery.

[3] Wan W. B., Seth P. P. (2016). The medicinal
chemistry of therapeutic oligonucleotides. J.
Med. Chem..

[4] Egli M., Manoharan M. (2023). Chemistry, structure and function
of approved oligonucleotide
therapeutics. Nucleic Acids Res..

[5] Crooke S. T., Seth P. P., Vickers T. A., Liang X. H. (2020). The interaction
of phosphorothioate-containing RNA targeted drugs with proteins is
a critical determinant of the therapeutic effects of these agents. J. Am. Chem. Soc..

[6] Crooke S. T., Vickers T. A., Liang X. H. (2020). Phosphorothioate
modified oligonucleotide-protein
interactions. Nucleic Acids Res..

[7] Obika S., Nanbu D., Hari Y., Morio K., In Y., Ishida T., Imanishi T. (1997). Synthesis
of 2′-*O*,4′-*C*-methyleneuridine
and -cytidine. Novel
bicyclic nucleosides having a fixed C3′-endo sugar puckering. Tetrahedron Lett..

[8] Obika S., Nanbu D., Hari Y., Andoh J., Morio K., Doi T., Imanishi T. (1998). Stability
and structural features of the duplexes containing
nucleoside analogues with a fixed N-type conformation, 2′-*O*,4′-*C*-methyleneribonucleosides. Tetrahedron Lett..

[9] Singh S. K., Nielsen P., Koshkin A. A., Wengel J. (1998). LNA (locked nucleic
acids): synthesis and high-affinity nucleic acid recognition. Chem. Commun..

[10] Koshkin A. A., Singh S. K., Nielsen P., Rajwanshi V. K., Kumar R., Meldgaard M., Olsen C. E., Wengel J. (1998). LNA (Locked
Nucleic Acids): Synthesis of the adenine, cytosine, guanine, 5-methylcytosine,
thymine and uracil bicyclonucleoside monomers, oligomerisation, and
unprecedented nucleic acid recognition. Tetrahedron.

[11] Swayze E. E., Siwkowski A. M., Wancewicz E. V., Migawa M. T., Wyrzykiewicz T. K., Hung G., Monia B. P., Bennett C. F. (2007). Antisense oligonucleotides
containing locked nucleic acid improve potency but cause significant
hepatotoxicity in animals. Nucleic Acids Res..

[12] Abdur
Rahman S. M., Seki S., Obika S., Yoshikawa H., Miyashita K., Imanishi T. (2008). Design, synthesis, and properties
of 2′,4′-BNA­(NC): A bridged nucleic acid analogue. J. Am. Chem. Soc..

[13] Seth P. P., Vasquez G., Allerson C. A., Berdeja A., Gaus H., Kinberger G. A., Prakash T. P., Migawa M. T., Bhat B., Swayze E. E. (2010). Synthesis and biophysical evaluation of 2′,4′-constrained
2′*O*-methoxyethyl and 2′,4′-constrained
2′*O*-ethyl nucleic acid analogues. J. Org. Chem..

[14] Yahara A., Shrestha A. R., Yamamoto T., Hari Y., Osawa T., Yamaguchi M., Nishida M., Kodama T., Obika S. (2012). Amido-bridged
nucleic acids (AmNAs): synthesis, duplex stability, nuclease resistance,
and in vitro antisense potency. ChemBioChem.

[15] Yamaguchi T., Horiba M., Obika S. (2015). Synthesis and properties of 2′-*O*,4′-*C*-spirocyclopropylene bridged
nucleic acid (scpBNA), an analogue of 2′,4′-BNA/LNA
bearing a cyclopropane ring. Chem. Commun..

[16] Horiba M., Yamaguchi T., Obika S. (2016). Synthesis of v-mC, -A, and -G monomers
and evaluation of the binding affinities of scpBNA-modified oligonucleotides
toward complementary ssRNA and ssDNA. J. Org.
Chem..

[17] Yamaguchi T., Horie N., Aoyama H., Kumagai S., Obika S. (2023). Mechanism
of the extremely high duplex-forming ability of oligonucleotides modified
with *N*-*tert*-butylguanidine- or *N*-*tert*-butyl-*N′*-methylguanidine-bridged nucleic acids. Nucleic
Acids Res..

[18] Hung G. N., Xiao X. K., Peralta R., Bhattacharjee G., Murray S., Norris D., Guo S. L., Monia B. P. (2013). Characterization
of target mRNA reduction through in situ RNA hybridization in multiple
organ systems following systemic antisense treatment in animals. Nucleic Acids Ther..

[19] Chappell A. E., Gaus H. J., Berdeja A., Gupta R., Jo M., Prakash T. P., Oestergaard M., Swayze E. E., Seth P. P. (2020). Mechanisms
of palmitic acid-conjugated antisense oligonucleotide distribution
in mice. Nucleic Acids Res..

[20] Tanaka Y., Tanioku Y., Nakayama T., Aso K., Yamaguchi T., Kamada H., Obika S. (2023). Synthesis of multivalent
fatty acid-conjugated
antisense oligonucleotides: Cell internalization, physical properties,
and in vitro and in vivo activities. Bioorg.
Med. Chem..

[21] Stein C. A., Hansen J. B., Lai J., Wu S., Voskresenskiy A., Høg A., Worm J., Hedtjärn M., Souleimanian N., Miller P., Soifer H. S., Castanotto D., Benimetskaya L., Ørum H., Koch T. (2010). Efficient gene silencing
by delivery of locked nucleic acid antisense oligonucleotides, unassisted
by transfection reagents. Nucleic Acids Res..

[22] Hori S.-i., Yamamoto T., Waki R., Wada S., Wada F., Noda M., Obika S. (2015). Ca^2+^ enrichment in culture
medium potentiates effect of oligonucleotides. Nucleic Acids Res..

[23] Moazami M. P., Rembetsy-Brown J. M., Sarli S. L., McEachern H. R., Wang F., Ohara M., Wagh A., Kelly K., Krishnamurthy P. M., Weiss A., Marosfoi M., King R. M., Motwani M., Gray-Edwards H., Fitzgerald K. A., Brown R. H., Watts J. K. (2024). Quantifying and mitigating motor
phenotypes induced by antisense oligonucleotides in the central nervous
system. Mol. Ther..

[24] Zhang L., Vickers T. A., Sun H., Liang X. H., Crooke S. T. (2021). Binding
of phosphorothioate oligonucleotides with RNase H1 can cause conformational
changes in the protein and alter the interactions of RNase H1 with
other proteins. Nucleic Acids Res..

[25] Vickers T. A., Rahdar M., Prakash T. P., Crooke S. T. (2019). Kinetic and subcellular
analysis of PS-ASO/protein interactions with P54nrb and RNase H1. Nucleic Acids Res..

[26] Seth P. P., Siwkowski A., Allerson C. R., Vasquez G., Lee S., Prakash T. P., Wancewicz E. V., Witchell D., Swayze E. (2009). Short antisense
oligonucleotides with novel 2′-4′ conformationally restricted
nucleoside analogues show improved potency without increased toxicity
in animals. J. Med. Chem..

[27] Shen W., De Hoyos C. L., Migawa M. T., Vickers T. A., Sun H., Low A., Bell T. A., Rahdar M., Mukhopadhyay S., Hart C. E., Bell M., Riney S., Murray S. F., Greenlee S., Crooke R. M., Liang X. H., Seth P. P., Crooke S. T. (2019). Chemical modification
of PS-ASO therapeutics reduces
cellular protein-binding and improves the therapeutic index. Nat. Biotechnol..

[28] Migawa M. T., Shen W., Wan W. B., Vasquez G., Oestergaard M. E., Low A., De Hoyos C. L., Gupta R., Murray S., Tanowitz M., Bell M., Nichols J. G., Gaus H., Liang X. H., Swayze E. E., Crooke S. T., Seth P. P. (2019). Site-specific replacement
of phosphorothioate with alkyl phosphonate linkages enhances the therapeutic
profile of gapmer ASOs by modulating interactions with cellular proteins. Nucleic Acids Res..

[29] Vasquez G., Freestone G. C., Wan W. B., Low A., De Hoyos C. L., Yu J., Prakash T. P., Ostergaard M. E., Liang X. H., Crooke S. T., Swayze E. E., Migawa M. T., Seth P. P. (2021). Site-specific incorporation
of 5′-methyl DNA enhances the therapeutic profile of gapmer
ASOs. Nucleic Acids Res..

[30] Prakash T. P., Yu J., Shen W., De Hoyos C. L., Berdeja A., Gaus H., Liang X. H., Crooke S. T., Seth P. P. (2021). Site-specific incorporation
of 2,5-linked nucleic acids enhances therapeutic profile of antisense
oligonucleotides. ACS Med. Chem. Lett..

[31] Anderson B. A., Freestone G. C., Low A., De-Hoyos C. L., Lii W. J. D., Ostergaard M. E., Migawa M. T., Fazio M., Wan W. B., Berdeja A., Scandalis E., Burel S. A., Vickers T. A., Crooke S. T., Swayze E. E., Liang X., Seth P. P. (2021). Towards next generation antisense
oligonucleotides: mesylphosphoramidate modification improves therapeutic
index and duration of effect of gapmer antisense oligonucleotides. Nucleic Acids Res..

[32] Yoshida T., Morihiro K., Naito Y., Mikami A., Kasahara Y., Inoue T., Obika S. (2022). Identification of nucleobase chemical
modifications that reduce the hepatotoxicity of gapmer antisense oligonucleotides. Nucleic Acids Res..

[33] Sakurai Y., Yamaguchi T., Yoshida T., Horiba M., Inoue T., Obika S. (2023). Synthesis
and properties of nucleobase-sugar dual modified nucleic
acids: 2′-OMe-RNA and scpBNA bearing a 5-hydroxycytosine nucleobase. J. Org. Chem..

[34] Kasuya T., Hori S., Watanabe A., Nakajima M., Gahara Y., Rokushima M., Yanagimoto T., Kugimiya A. (2016). Ribonuclease H1-dependent
hepatotoxicity caused by locked nucleic acid-modified gapmer antisense
oligonucleotides. Sci. Rep..

[35] Yasuhara H., Yoshida T., Sasaki K., Obika S., Inoue T. (2022). Reduction
of off-target effects of gapmer antisense oligonucleotides by oligonucleotide
extension. Mol. Diagn. Ther..

[36] Stanton R., Sciabola S., Salatto C., Weng Y., Moshinsky D., Little J., Walters E., Kreeger J., DiMattia D., Chen T., Clark T., Liu M., Qian J., Roy M., Dullea R. (2012). Chemical modification
study of antisense gapmers. Nucleic Acid Ther..

[37] Geary R. S., Yu R. Z., Watanabe T., Henry S. P., Hardee G. E., Chappell A., Matson J., Sasmor H., Cummins L., Levin A. A. (2003). Pharmacokinetics of a tumor necrosis factor-alpha phosphorothioate
2′-*O*-(2-methoxyethyl) modified antisense oligonucleotide:
comparison across species. Drug Metab. Dispos..

[38] Kawanobe T., Asano S., Kandori H., Aoki M., Shrestha A. R., Sekiguchi K., Yokoyama K., Fukuda R., Umemoto T. (2025). Hepatotoxicity
reduction profiles of antisense oligonucleotides containing amido-bridged
nucleic acid and 2′-O,4′-C-spirocyclopropylene bridged
nucleic acid. Nucleic Acid Ther..

