# Supplementary material for: Cycloalkane Incorporation Into the 2′,4′-Bridge of Locked Nucleic Acid: Enhancing Nuclease Stability, Reducing Phosphorothioate Modifications, and Lowering Hepatotoxicity in Antisense Oligonucleotides
Source: JACS Au. 2025 Oct 15;5(10):5111–20. doi: 10.1021/jacsau.5c01005 (PMC12569656; doi:10.1021/jacsau.5c01005)

Supplementary Information

Cycloalkane incorporation into the 2′,4′-bridge of locked nucleic acid: enhancing nuclease stability, reducing phosphorothioate modifications, and lowering hepatotoxicity in antisense oligonucleotides

Takao Yamaguchi,[1,*] Hibiki Komine,[1,†] Takaya Sugiura,[1,†] Riku Kumagai,[1] Tokuyuki Yoshida,[2] Kiyomi Sasaki,[2] Taisuke Nakayama,[3] Haruhiko Kamada,[3] Takao Inoue,[2] and Satoshi Obika[1,3,4,*]

[1]*Graduate School of Pharmaceutical Sciences, The University of Osaka, 1-6 Yamadaoka, Suita, Osaka 565-0871, Japan*

[2]*National Institute of Health Sciences, 3-25-26 Tonomachi, Kawasaki-ku, Kawasaki, Kanagawa, 210-9501, Japan*

[3]*National Institutes of Biomedical Innovation, Health and Nutrition, 7-6-8 Saito-Asagi, Ibaraki, Osaka-567-0085, Japan*

[4]*Institute for Open and Transdisciplinary Research Initiatives, The University of Osaka, 1-1 Yamadaoka, Suita, Osaka 565-0871, Japan*

*E-mail: yamaguchi-ta@phs.osaka-u.ac.jp (TY), obika@phs.osaka-u.ac.jp (SO)

[†]These authors contributed equally to this work.

Table of Contents

1. Supplementary data
2. Procedures for phosphoramidite synthesis
3. ^{1}H NMR, ^{13}C NMR, and ^{31}P NMR spectra of novel compounds
4. Characterization data (HPLC and mass data) of synthesized oligonucleotides

1. Supplementary data

scpBNA2-modified oligonucleotide (**ON5**)

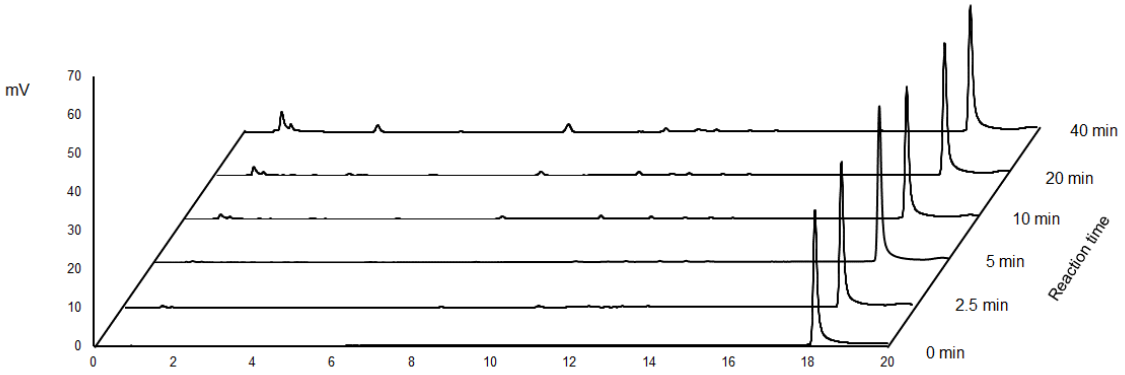

PS-modified oligonucleotide (**ON11**)

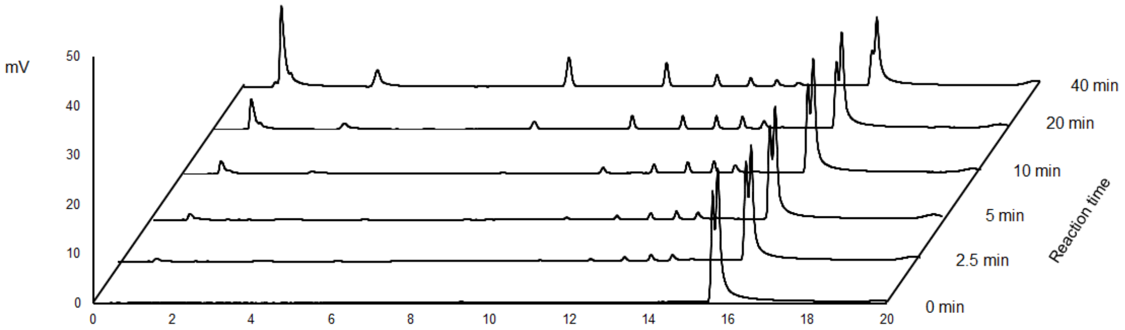

scpBNA-modified oligonucleotide (**ON12**)

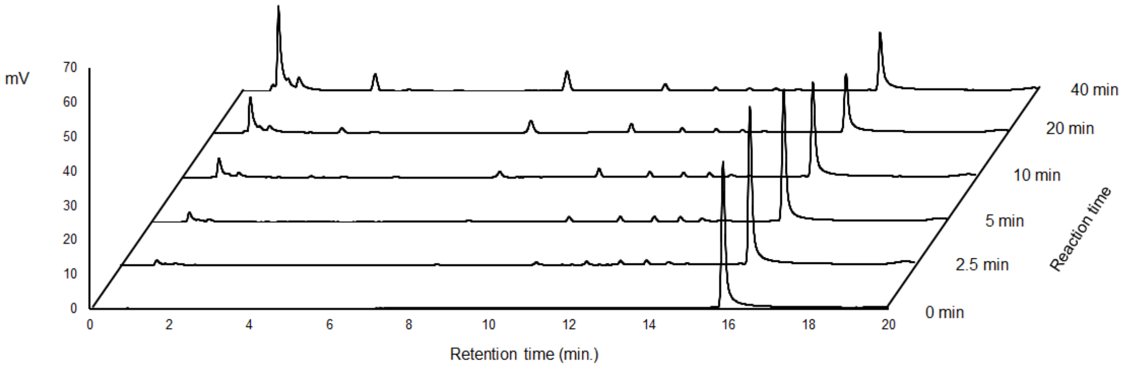

Figure S1. HPLC analysis of the nuclease stability of 5′-d(TTTTTTTTT**X**)-3′ against 3′-exonuclease (snake venom phosphodiesterase, svPDE). **X** = scpBNA2-T (green diamonds, **ON5**), 5′-PS-modified thymidine (black circles, **ON11**), or scpBNA-T (blue squares, **ON12**). Conditions: 50 mM Tris-HCl (pH 8.0), 10 mM $MgCl_2$, 7.5 µM of each oligonucleotide, and 1.5 µg/mL svPDE at 37 °C. Column: Waters XBridge™OST C18 2.5 µm (4.6 × 50 mm). Mobile phase: linear gradient of MeCN (5%–15.5% for 20 min) in 0.1 M triethylammonium acetate (pH 7.0). Flow rate: 1.0 mL/min. Detection: absorbance at 260 nm.

Table S1. Isolated yields and MALDI-TOF MS data of scpBNA- and scpBNA2-modified ASOs targeting *Malat1*

ID	Sequence [5′-(^{m}CTAGTTCACTGAATG^{m}C)-3′, 16-mer][a]	Yield (%)	Calcd. [M-H]$^{-}$	Found [M-H]$^{-}$
**ON13**	●^●^●^●^●^●^●^●^●^●^●^●^●^●^●^●	–[b]	5288.5	5293.0
**ON14**	●O●O●^●^●^●^●^●^●^●^●^●^●^●^●O●^●	13	5240.6	5241.8
**ON15**	●O●O●^●^●^●^●^●^●^●^●^●^●^●O●O●O●	14	5208.6	5209.3
**ON16**	●^●^●^●^●^●^●^●^●^●^●^●^●^●^●^●	28	5392.6	5392.6
**ON17**	●O●O●^●^●^●^●^●^●^●^●^●^●^●^●O●^●	18	5344.6	5344.2
**ON18**	●O●O●^●^●^●^●^●^●^●^●^●^●^●O●O●O●	18	5312.7	5312.6
**ON19**	●^●^●^●^●^●^●^●^●^●^●^●^●^●^●^●	11	5504.7	5505.3
**ON20**	●O●O●^●^●^●^●^●^●^●^●^●^●^●^●O●^●	25	5456.8	5457.2
**ON21**	●O●O●^●^●^●^●^●^●^●^●^●^●^●O●O●O●	15	5424.8	5425.0

[a]Marks "^" and "O" indicate phosphorothioate (PS) and phosphodiester (PO) linkages, respectively. Red, black, blue, and green circles indicate 2′,4′-BNA/LNA, DNA, scpBNA, and scpBNA2, respectively. ^{m}C represents 5-methylcytosine. [b]Synthesized by GeneDesign, Inc.

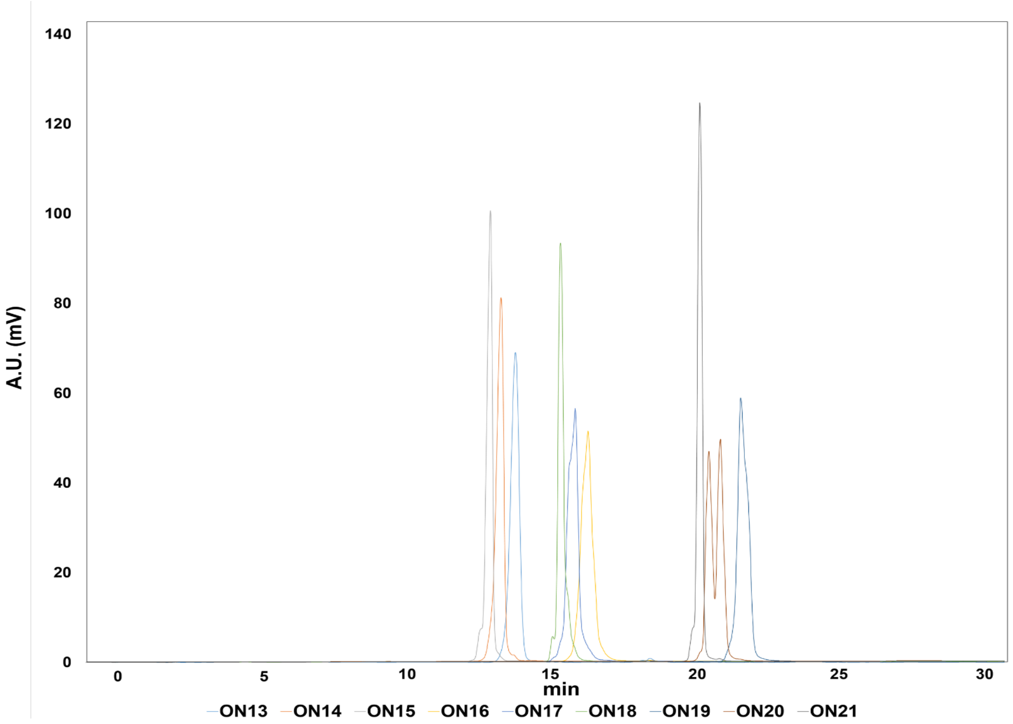

Figure S2. HPLC analysis of **ON13–ON21**. The samples were analyzed using a reverse-phase HPLC column (Waters Xterra® MS C18 2.5 µm, 4.6×50 mm column; eluent A (water/HFIP/triethylamine: 100/1/0.1) and eluent B (MeOH), eluted with a linear gradient of 10%–35% of eluent B.

Table S2. T_m values (°C) of duplexes formed between oligonucleotides and complementary ssRNA[a]

ID	Sequence [5′-(^{m}CTAGTTCACTGAATG^{m}C)-3′, 16-mer][b]	T_m (°C) toward complementary ssRNA
**ON13**	🔴^🔴^🔴^⚫^⚫^⚫^⚫^⚫^⚫^⚫^⚫^⚫^⚫^🔴^🔴^🔴	63
**ON14**	🔴O🔴O🔴^⚫^⚫^⚫^⚫^⚫^⚫^⚫^⚫^⚫^⚫^🔴O🔴^🔴	64
**ON15**	🔴O🔴O🔴^⚫^⚫^⚫^⚫^⚫^⚫^⚫^⚫^⚫^⚫O🔴O🔴O🔴	65
**ON16**	🔵^🔵^🔴^⚫^⚫^⚫^⚫^⚫^⚫^⚫^⚫^⚫^⚫^🔵^🔴^🔵	62
**ON17**	🔵O🔵O🔴^⚫^⚫^⚫^⚫^⚫^⚫^⚫^⚫^⚫^⚫^🔵O🔴^🔵	64
**ON18**	🔵O🔵O🔴^⚫^⚫^⚫^⚫^⚫^⚫^⚫^⚫^⚫^⚫O🔵O🔴O🔵	65
**ON19**	🟢^🟢^🔴^⚫^⚫^⚫^⚫^⚫^⚫^⚫^⚫^⚫^⚫^🟢^🔴^🟢	62
**ON20**	🟢O🟢O🔴^⚫^⚫^⚫^⚫^⚫^⚫^⚫^⚫^⚫^⚫^🟢O🔴^🟢	64
**ON21**	🟢O🟢O🔴^⚫^⚫^⚫^⚫^⚫^⚫^⚫^⚫^⚫^⚫O🟢O🔴O🟢	64

[a]Conditions: 10 mM sodium phosphate buffer (pH 7.2), 100 mM NaCl, and 4 μM of each oligonucleotide. Sequence of the complementary ssRNA: 5′-r(GCAUUCAGUGAACUAG)-3′. T_m values reflect the average of at least three measurements. [b]Marks "^" and "O" indicate phosphorothioate (PS) and phosphodiester (PO) linkages, respectively. Red, black, blue, and green circles indicate 2′,4′-BNA/LNA, DNA, scpBNA, and scpBNA2, respectively. ^{m}C represents 5-methylcytosine.

Table S3. Isolated yields and MALDI-TOF mass data of scpBNA- and scpBNA2-modified ASOs targeting *Malat1*

ID	Sequence [5′-(^{m}CTAGTTCACTGAATG^{m}C)-3′, 16-mer][a]	Yield (%)	Calcd. $[M–H]^-$	Found $[M–H]^-$
**ON13**	🔴^🔴^🔴^⚫^⚫^⚫^⚫^⚫^⚫^⚫^⚫^⚫^⚫^🔴^🔴^🔴	–[b]	5288.5	5293.0
**ON25**	🔴O🔴O🔴O⚫^⚫^⚫^⚫^⚫^⚫^⚫^⚫^⚫^⚫O🔴O🔴O🔴	16	5192.6	5193.3
**ON26**	🔵^🔵^🔵^⚫^⚫^⚫^⚫^⚫^⚫^⚫^⚫^⚫^⚫^🔵^🔵^🔵	17	5444.6	5444.3
**ON27**	🔵O🔵O🔵O⚫^⚫^⚫^⚫^⚫^⚫^⚫^⚫^⚫^⚫O🔵O🔵O🔵	18	5348.7	5348.9
**ON28**	🟢^🟢^🔵^⚫^⚫^⚫^⚫^⚫^⚫^⚫^⚫^⚫^⚫^🟢^🔵^🟢	7	5556.7	5556.7
**ON29**	🟢O🟢O🔵O⚫^⚫^⚫^⚫^⚫^⚫^⚫^⚫^⚫^⚫O🟢O🔵O🟢	10	5460.9	5460.8

[a]Marks "^" and "O" indicate phosphorothioate (PS) and phosphodiester (PO) linkages, respectively. Red, black, blue, and green circles indicate 2′,4′-BNA/LNA, DNA, scpBNA, and scpBNA2, respectively. ^{m}C represents 5-methylcytosine. [b]Synthesized by GeneDesign, Inc.

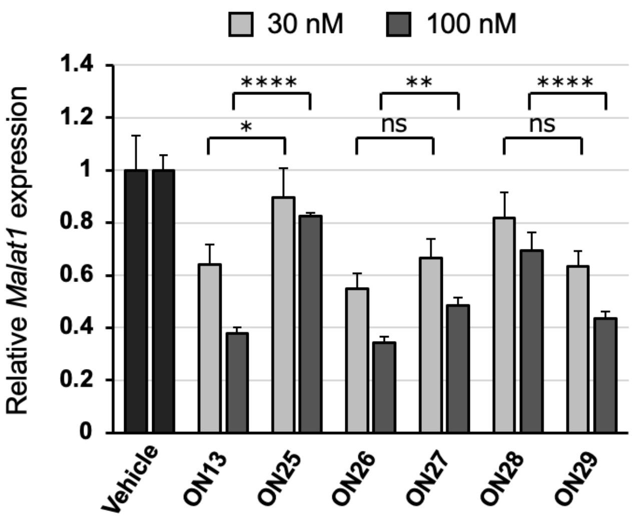

Figure S3. Real-time qPCR analysis of relative *Malat1* RNA expression levels in NMuLi cells after gymnosis treatment of ASOs for 48 h. NMuLi cells were incubated with 30 or 100 nM of ASOs at 37 °C in an incubator containing 95% air and 5% CO_2 atmosphere. After incubation for 48 h, total RNA was isolated and the expression levels of *Malat1* RNA were quantified. This experiment was conducted in triplicate (n = 3 per group). Data are presented as mean ± SD. Statistical significance was established using the Tukey's multiple comparison test. **** P < 0.0001; ** P < 0.01; * P < 0.05; ns, not significant.

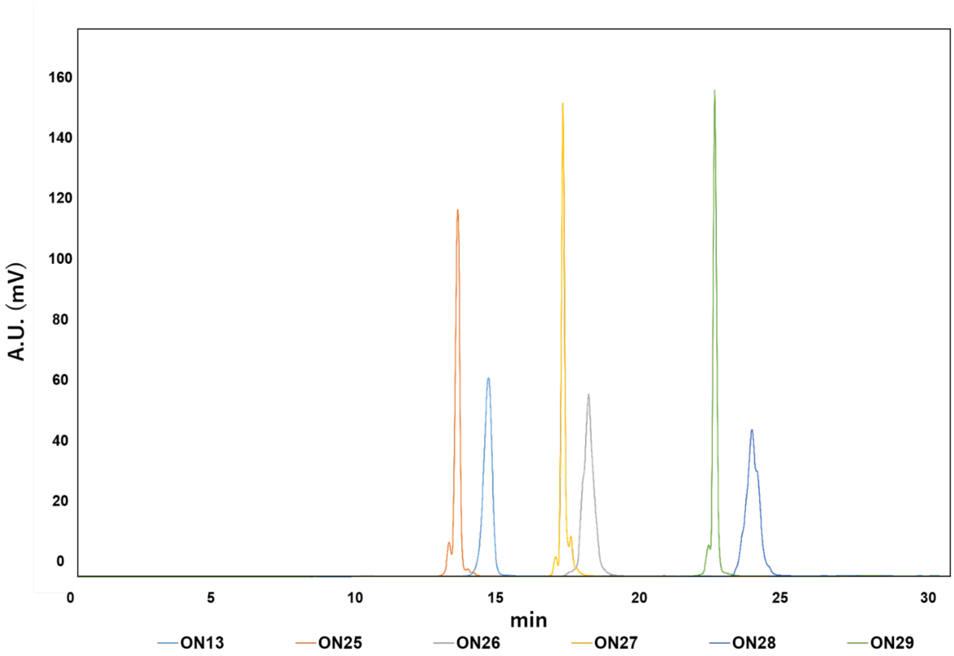

Figure S4. HPLC analysis of **ON13** and **ON25–ON29**. Samples were analyzed using reverse-phase HPLC (Waters Xterra® MS C18 2.5 µm, 4.6×50 mm column; eluent A (water/HFIP/triethylamine: 100/1/0.1) and eluent B (MeOH), eluted with a linear gradient of 10%–35% of eluent B).

Table S4. T_m values (°C) of duplexes formed between oligonucleotides and complementary ssRNA[a]

ID	Sequence [5′-(^{m}CTAGTTCACTGAATG^{m}C)-3′, 16-mer][b]	T_m (°C) toward complementary ssRNA
**ON13**	●^●^●^●^●^●^●^●^●^●^●^●^●^●^●^●	63
**ON25**	●O●O●O●^●^●^●^●^●^●^●^●^●O●O●O●	66
**ON26**	●^●^●^●^●^●^●^●^●^●^●^●^●^●^●^●	62
**ON27**	●O●O●O●^●^●^●^●^●^●^●^●^●O●O●O●	65
**ON28**	●^●^●^●^●^●^●^●^●^●^●^●^●^●^●^●	61
**ON29**	●O●O●O●^●^●^●^●^●^●^●^●^●O●O●O●	64

[a]Conditions: 10 mM sodium phosphate buffer (pH 7.2), 100 mM NaCl, and 4 µM of each oligonucleotide. The sequence of complementary ssRNA is 5′-r(GCAUUCAGUGAACUAG)-3′. T_m values reflect the average of at least three measurements. [b]Marks "^" and "O" indicate phosphorothioate (PS) and phosphodiester (PO) linkages, respectively. Red, black, blue, and green circles indicate 2′,4′-BNA/LNA, DNA, scpBNA, and scpBNA2, respectively. ^{m}C represents 5-methylcytosine.

Table S5. Isolated yields and MALDI-TOF mass data of scpBNA- and scpBNA2-modified ASOs (non-target)

**ID**	Sequence [5′-(GTTATGCCACC^{m}CTA)-3′][a]	Yield (%)	Calcd. [M–H]$^{-}$	Found [M–H]$^{-}$
**ON22**	●^●^●^●^●^●^●^●^●^●^●^●^●^●^●	–[b]	4589.7	4589.9
**ON23**	●^●^●^●^●^●^●^●^●^●^●^●^●^●^●	–[b]	4667.8	4665.8
**ON24**	●^●^●^●^●^●^●^●^●^●^●^●^●^●^●	19	4747.1	4747.6

[a]Marks "^" and "O" indicate phosphorothioate (PS) and phosphodiester (PO) linkages, respectively. Red, black, blue, and green circles indicate 2′,4′-BNA/LNA, DNA, scpBNA, and scpBNA2, respectively. ^{m}C represents 5-methylcytosine. [b]Synthesized by GeneDesign, Inc.

Table S6. Isolated yields and MALDI-TOF mass data of scpBNA- and scpBNA2-modified ASOs targeting *Nr3c1*

ID	Sequence [5′-(GT^{m}CTCTTTACCTGG)-3′][a]	Yield (%)	Calcd. [M–H]$^-$	Found [M–H]$^-$
**ON30**	●^●^●^●^●^●^●^●^●^●^●^●^●^●^●^●	–[b]	4611.7	4613.2
**ON31**	●O●O●O●^●^●^●^●^●^●^●^●^●O●O●O●	–[b]	4515.3	4514.3
**ON32**	●^●^●^●^●^●^●^●^●^●^●^●^●^●^●^●	–[b]	4768.0	4769.9
**ON33**	●O●O●O●^●^●^●^●^●^●^●^●^●O●O●O●	–[b]	4671.6	4672.7
**ON34**	●^●^●^●^●^●^●^●^●^●^●^●^●^●^●^●	21	4847.7	4847.4
**ON35**	●O●O●O●^●^●^●^●^●^●^●^●^●O●O●O●	20	4751.8	4752.5

[a]Marks "^" and "O" indicate phosphorothioate (PS) and phosphodiester (PO) linkages, respectively. Red, black, blue, and green circles indicate 2′,4′-BNA/LNA, DNA, scpBNA, and scpBNA2, respectively. ^{m}C represents 5-methylcytosine. [b]Synthesized by GeneDesign, Inc.

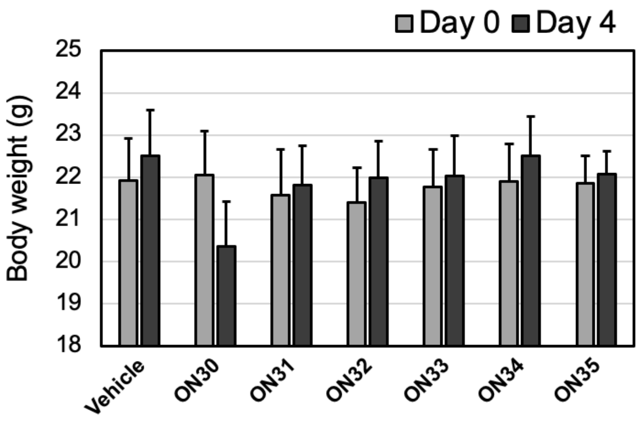

Figure S5. Mice (C57BL/6NCrl mice, female, six-week-old, n = 5 per group) were injected intravenously with a single dose of 100 nmol of each oligonucleotide. Weight changes were evaluated at 96 h post-administration.

2. Procedures for phosphoramidite synthesis

*Synthesis of Compound **2***

To the solution of **1** (3.69 g, 8.61 mmol) and cerium (III) chloride anhydrous (7.99 g, 32.4 mmol) in dry tetrahydrofuran (60 mL) was added 1M allylmagnesium bromide in tetrahydrofuran (33.0 mL, 33.0 mmol) at 0 °C. After stirring at room temperature for 14 h, saturated aq. NH_4Cl was added and the organic layer was then removed under reduced pressure. The residual aqueous solution was filtered through Celite® and extracted with AcOEt. The combined organic layer was washed with water and brine, dried over Na_2SO_4, and concentrated. The crude product was purified by column chromatography (SiO_2, *n*-hexane : AcOEt = 32 : 1 to 6 : 1) to afford **2** (4.06 g, 98%) as a colorless paste. Compound **2**: ^{1}H NMR (300 MHz, $CDCl_3$) δ 7.24–7.29 (m, 10H), 5.84–5.98 (m, 3H), 4.88–5.00 (m, 4H), 4.85 (d, *J* = 10.7 Hz, 1H), 4.78 (dd, *J* = 4.1, 5.5 Hz, 1H), 4.53 (d, *J* = 12.0 Hz, 1H), 4.42 (d, *J* = 11.7 Hz, 1H), 4.37–4.41 (m, 2H), 4.02 (bs, 1H), 3.66 (d, *J* = 9.6 Hz, 1H), 3.58 (d, *J* = 9.3 Hz, 1H), 2.76 (dd, *J* = 5.8, 14.4 Hz, 1H), 2.41–2.52 (m, 2H), 2.28 (dd, *J* = 7.6, 14.4 Hz, 1H), 1.61 (s, 3H), 1.39 (s, 3H); ^{13}C NMR (75.6 MHz, $CDCl_3$) δ 137.7, 136.9, 134.9, 134.7, 128.5, 128.1, 128.1, 127.8, 127.5, 117.1, 117.1, 114.7, 105.9, 92.6, 81.7, 80.9, 76.3, 74.5, 73.7, 73.5, 41.2, 40.6, 27.7, 27.2; HRMS (MALDI) Calcd. for $C_{29}H_{36}O_6Na$ $[M+Na]^+$: 503.2404, Found 503.2404.

*Synthesis of Compound **3***

To the solution of **2** (1.60 g, 3.54 mmol) in dry dichloromethane (110 mL) was added Grubbs 2nd catalyst (425 mg, 0.501 mmol) at room temperature. After stirring at room temperature for 4 h, dichloromethane was removed under reduced pressure. The crude product was purified by column chromatography (SiO_2, *n*-hexane : AcOEt = 32 : 1 to 10 : 1) to afford **3** (1.35 g, 90%) as a yellow paste. Compound **3**: ^{1}H NMR (300 MHz, $CDCl_3$) δ 7.28–7.36 (m, 10H), 5.92 (d, *J* = 3.5 Hz, 1H), 5.58–5.65 (m, 2H), 4.87 (d, *J* = 11.3 Hz, 1H), 4.77 (dd, *J* = 4.1, 8.6 Hz, 1H), 4.54 (d, *J* = 12.0 Hz, 1H), 4.38–4.47 (m, 3H), 3.75 (bs, 1H), 3.53 (d, *J* = 9.6 Hz, 1H), 3.16 (bs, 2H), 2.62–2.81 (m, 1H), 2.34–2.45 (m, 1H), 2.29 (d, *J* = 16.8 Hz, 1H), 1.63 (s, 3H), 1.38 (s, 3H); ^{13}C NMR (75.6 MHz, $CDCl_3$) δ 138.0, 137.2, 128.5, 128.4, 128.1, 128.1, 127.7, 127.4, 127.0, 114.6, 105.7, 92.1, 83.0, 80.8, 80.5, 75.1, 73.7, 73.4, 45.1, 27.3, 27.1; HRMS (MALDI) Calcd. for $C_{27}H_{32}O_6Na$ $[M+Na]^+$: 475.2091, Found 475.2091.

*Synthesis of Compound **4***

To the solution of **3** (1.21 g, 2.67 mmol) in dry dichloromethane (31 mL) was added 2,6-lutidine (2.20 mL, 19.0 mmol) and *tert*-butyldimethylsilyl trifluoromethanesulfonate (3.00 mL, 13.1 mmol) at 0 °C. After stirring at room temperature for 20 h, saturated aq. $NaHCO_3$ was added and the resulting mixture was extracted with AcOEt. The combined organic layer was washed with water and brine, dried over Na_2SO_4, and concentrated. The crude product was purified by column chromatography (SiO_2, *n*-hexane : AcOEt = 1 : 0 to 9 : 1) to afford **4** (1.43 g, 94%) as a colorless paste. Compound **4**: ^{1}H NMR (300 MHz, $CDCl_3$) δ 7.26–7.40 (m, 10H), 5.93 (d, *J* = 4.5 Hz, 1H), 5.60–5.63

(m, 2H), 4.88 (dd, *J* = 4.8, 5.9 Hz, 1H), 4.78 (d, *J* = 11.0 Hz, 1H), 4.58 (d, *J* = 11.7 Hz, 1H), 4.45 (d, *J* = 12.0 Hz, 1H), 4.38 (d, *J* = 11.0 Hz, 1H), 4.14 (d, *J* = 5.8 Hz, 1H), 3.89 (d, *J* = 8.2 Hz, 1H), 3.61 (d, *J* = 9.3 Hz, 1H), 2.88–3.15 (m, 2H), 2.39–2.70 (m, 1H), 2.20 (d, *J* = 16.8 Hz, 1H), 1.51 (s, 3H), 1.36 (s, 3H), 0.76 (s, 9H), –0.04 (s, 3H), –0.14 (s, 3H); ^{13}C NMR (75.6 MHz, $CDCl_3$) δ 138.5, 138.1, 128.5, 127.9, 127.7, 127.5, 127.1, 114.3, 105.6, 86.4, 82.5, 80.5, 76.0, 73.8, 73.6, 45.5, 44.2, 28.0, 27.4, 26.0, 18.3, –3.5, –3.6; HRMS (MALDI) Calcd. for $C_{33}H_{46}O_6NaSi$ $[M+Na]^+$: 589.2956, Found 589.2956.

Synthesis of Compound **5**

To the solution of **4** (911 mg, 1.61 mmol) in acetic acid (1.85 mL, 32.3 mmol) was added acetic anhydride (3.05 mL, 32.3 mmol) and trifluoroacetic acid (295 µL, 3.86 mmol) at 0 °C. After stirring at room temperature for 2 h, saturated aq. $NaHCO_3$ was added and the resulting mixture was extracted with AcOEt. The combined organic layer was washed with water and brine, dried over Na_2SO_4, and concentrated. The crude product was purified by column chromatography (SiO_2, *n*-hexane : AcOEt = 9 : 1 to 4 : 1) to afford **5** (841 mg, 86%) as a colorless paste. Compound **5**: ^{1}H NMR (300 HMz, $CDCl_3$) δ 7.26–7.43 (m, 10H), 6.49 (d, *J* = 5.1 Hz, 1/2H), 6.35 (d, *J* = 5.1 Hz, 1/2H), 5.67–5.71 (m, 3/2H), 5.50–5.64 (m, 3/2H), 4.62–4.74 (m, 2H), 4.32–4.56 (m, 3H), 4.00 (d, *J* = 10.0 Hz, 1/2H), 3.97 (d, *J* = 12.0 Hz, 1/2H), 3.59 (d, *J* = 6.9 Hz, 1/2H), 3.57 (d, J = 6.9 Hz, 1/2H), 2.82–3.18 (m, 2H), 2.44 (d, *J* = 18.2 Hz, 1H), 2.16–2.25 (m, 1H), 2.11 (s, 3/2H), 2.04 (s, 3/2H), 1.89 (s, 3/2H), 1.84 (s, 3/2H), 0.79 (s, 9/2H), 0.78 (s, 9/2H), –0.01 (s, 3/2H), –0.03 (s, 3/2H), –0.10 (s, 3/2H), –0.13 (s, 3/2H); HRMS (MALDI) Calcd. for $C_{34}H_{46}O_8NaSi$ $[M+Na]^+$: 633.2854, Found 633.2851.

Synthesis of Compound **6**

To the solution of **5** (389 mg, 0.637 mmol) in dry acetonitrile (5.6 mL) was added thymine (242 mg, 1.91 mmol), *N*,*O*-bis-trimethylsilylacetoamide (780 µL, 3.18 mmol) at room temperature. After stirring at room temperature for 1 h, was dropped trimethylsilyl trifluoromethanesulfonate (175 µL, 0.969 mmol) at 0 °C. After refluxing for 5 h, saturated aq. $NaHCO_3$ was added and the resulting mixture was extracted with AcOEt. The combined organic layer was washed with water and brine, dried over Na_2SO_4, and concentrated. The crude product was purified by column chromatography (SiO_2, *n*-hexane : AcOEt = 9 : 1 to 4 : 1) to afford **6** (385 mg, 89%) as a white solid. Compound **6**: ^{1}H NMR (300 MHz, $CDCl_3$) δ 8.33 (s, 1H), 7.66 (d, *J* = 1.0 Hz, 1H), 7.27–7.46 (m, 10H), 6.42 (d, *J* = 8.9 Hz, 1H), 5.57–5.65 (m, 3H), 4.79 (d, *J* = 11.3 Hz, 1H), 4.70 (d, *J* = 11.7 Hz, 1H), 4.64 (d, *J* = 11.7 Hz, 1H), 4.49 (d, *J* = 11.3 Hz, 1H), 4.44 (d, *J* = 5.1 Hz, 1H), 4.06 (d, *J* = 9.6 Hz, 1H), 3.76 (d, *J* = 10.0 Hz, 1H), 2.83 (d, *J* = 16.8 Hz, 2H), 2.57 (d, *J* = 17.9 Hz, 1H), 2.22 (d, *J* = 16.1 Hz, 1H), 1.90 (s, 3H), 1.59 (s, 3H), 0.80 (s, 9H), –0.01 (s, 3H), –0.13 (s, 3H); ^{13}C NMR (75.6 MHz, $CDCl_3$) δ 170.7, 163.4, 150.7, 138.1, 137.1, 136.0, 129.2, 128.8, 128.3, 128.2, 127.7, 127.5, 127.3, 127.0, 111.5, 91.3, 87.4, 84.6, 81.3, 75.5, 75.0, 73.9, 73.8, 44.9, 44.5, 26.3, 20.6, 18.4, 12.2, –3.4, –3.4; HRMS (MALDI) Calcd. for $C_{37}H_{48}N_2O_8NaSi$ $[M+Na]^+$: 699.3072, Found 699.3069.

Synthesis of Compound 7

To the solution of **6** (986 mg, 1.46 mmol) in methanol (14.6 mL) was added potassium carbonate (607 mg, 4.39 mmol) at 0 °C, and the reaction mixture was stirred at room temperature for 5 h, water was added and the resulting mixture was extracted with Et_2O. The combined organic layer was washed with water and brine, dried over Na_2SO_4, and concentrated. The crude product was purified by column chromatography (SiO_2, *n*-hexane : AcOEt = 4 : 1 to 1 : 1) to afford **7** (895 mg, 97%) as a white solid. Compound **7**: ^{1}H NMR (300 MHz, $CDCl_3$) δ 8.32 (s, 1H), 7.58 (s, 1H), 7.26–7.41 (m, 10H), 5.99 (d, *J* = 8.2 Hz, 1H), 5.63–5.69 (m, 2H), 5.00 (d, *J* = 10.3 Hz, 1H), 4.66 (d, *J* = 11.7 Hz, 1H), 4.58 (d, *J* = 11.7 Hz, 1H), 4.57 (d, *J* = 11.0 Hz, 2H), 4.22 (d, *J* = 5.5 Hz, 1H), 4.06 (d, *J* = 8.6 Hz, 1H), 3.73 (d, *J* = 9.6 Hz, 1H), 2.60–2.87 (m, 4H), 2.24 (d, *J* = 16.8 Hz, 1H), 1.63 (s, 3H), 0.82 (s, 9H), 0.04 (s, 3H), –0.03 (s, 3H); ^{13}C NMR (75.6 MHz, $CDCl_3$) δ 163.3, 150.9, 147.3, 144.8, 137.2, 137.0, 136.0, 128.9, 128.8, 128.7, 128.2, 128.1, 127.6, 127.6, 111.3, 90.7, 87.6, 83.2, 75.9, 75.0, 74.0, 44.7, 44.6, 26.3, 18.4, 12.2, –3.2, –3.3; HRMS (MALDI) Calcd. for $C_{35}H_{46}N_2O_7NaSi$ $[M+Na]^+$: 657.2966, Found 657.2963.

*Synthesis of Compound **8***

To the solution of **7** (895mg, 1.41 mmol) in dry pyridine (14.5 mL) was added methanesulfonyl chloride (170 µL, 2.19 mmol) at 0 °C, and the reaction mixture was stirred at room temperature for 4 h. After addition of water, the resulting mixture was extracted with AcOEt. The combined organic layer was washed with water and brine, dried over Na_2SO_4, and concentrated. The crude product was purified by column chromatography (SiO_2, *n*-hexane : AcOEt = 9 : 1 to 4 : 1) to afford **8** (992 mg, quant.) as a white solid. Compound **8**: ^{1}H NMR (300 MHz, $CDCl_3$) δ 8.52 (s, 1H), 7.58 (s, 1H), 7.29–7.42 (m, 10H), 6.40 (d, *J* = 8.3 Hz, 1H), 5.67 (d, *J* = 8.3 Hz, 1H), 5.65 (d, *J* = 8.2 Hz, 1H), 5.58 (d, *J* = 5.5 Hz, 1H), 4.80 (d, *J* = 11.0 Hz, 1H), 4.76 (d, *J* = 11.3 Hz, 1H), 4.69 (d, *J* = 11.3 Hz, 1H), 4.64 (d, *J* = 11.7 Hz, 1H), 4.36 (d, *J* = 5.2 Hz, 1H), 4.04 (d, *J* = 10.0 Hz, 1H), 3.78 (d, *J* = 9.6 Hz, 1H), 2.84 (s, 3H), 2.76–2.82 (m, 2H), 2.58 (d, *J* = 17.9 Hz, 1H), 2.21 (d, *J* = 16.8 Hz, 1H), 1.59 (s, 3H), 0.81 (s, 9H), –0.01 (s, 3H), –0.12 (s, 3H); ^{13}C NMR (75.6 MHz, $CDCl_3$) δ 163.3, 150.7, 137.8, 136.7, 135.3, 129.2, 128.9, 128.3, 128.3, 127.7, 127.5, 127.2, 111.9, 91.2, 87.2, 84.3, 81.3, 75.0, 74.0, 73.5, 44.7, 44.4, 38.0, 26.2, 18.3, 12.2, –3.4, –3.5; HRMS (MALDI) Calcd. for $C_{36}H_{48}N_2O_9NaSiS$ $[M+Na]^+$: 735.2742, Found 735.2718.

*Synthesis of Compound **9***

To the solution of **8** (992 mg, 1.39 mmol) in tetrahydrofuran (13.9 mL) was added 1M tetrabutylammoniumfluoride in tetrahydrofuran (4.18 mL, 4.18 mmol) at 0 °C, and the reaction mixture was stirred at room temperature for 30 h. After addition of water, the resulting mixture was extracted with AcOEt. The combined organic layer was washed with water and brine, dried over Na_2SO_4, and concentrated. The crude paste was used immediately for the next reaction without further purification. To the solution of this crude in *N*,*N*-dimethylformamide (13.9 mL) was added potassium carbonate (577 mg, 4.17 mmol) at 0 °C, and the reaction mixture was stirred at 90 °C for 20 h. After

addition of water, the resulting mixture was extracted with Et_2O. The combined organic layer was washed with water and brine, dried over Na_2SO_4, and concentrated. The crude product was purified by column chromatography (SiO_2, *n*-hexane : AcOEt = 4 : 1 to 1 : 2) to afford **9** (583 mg, 83% in 2 steps) as a white solid. Compound **9**: ^{1}H NMR (300 MHz, $CDCl_3$) δ 8.47 (s, 1H), 7.55 (s, 1H), 7.25–7.33 (m, 10H), 5.63–5.67 (m, 2H), 5.55 (s, 1H), 4.65 (d, *J* = 11.3 Hz, 1H), 4.61 (s, 2H), 4.51 (d, *J* = 11.3 Hz, 1H), 4.51 (s, 1H), 4.05 (s, 1H), 3.87 (d, *J* = 11.0 Hz, 1H), 3.81 (d, *J* = 11.0 Hz, 1H), 2.94 (d, *J* = 18.2 Hz, 1H), 2.66 (s, 2H), 2.58 (d, *J* = 17.9 Hz, 1H), 1.57 (s, 3H); ^{13}C NMR (75.6 MHz, $CDCl_3$) δ 163.8, 149.8, 137.5, 136.9, 135.1, 128.6, 128.5, 128.3, 128.1, 127.7, 127.6, 127.4, 110.0, 92.8, 88.5, 86.4, 77.7, 77.5, 73.9, 72.2, 64.9, 42.3, 41.5, 12.2; HRMS (MALDI) Calcd. for $C_{29}H_{30}N_2O_6Na$ $[M+Na]^+$: 525.1996, Found 525.1997.

*Synthesis of Compound **10***

To the solution of **9** (110 mg, 0.219 mmol) in AcOEt (2.15 mL) was added palladium hydroxide 20% on carbon (44.0 mg, 40wt%). The reaction flask was degassed a few times with H_2 and the reaction mixture was stirred at room temperature for 0.5 h under H_2 atmosphere. After completion of reaction, the reaction mixture was filtered, washed by AcOEt and concentrated. The crude product was purified by preparative layer plates (PLC) (SiO_2, $CHCl_3$: MeOH = 7 : 1) to afford **10** (57.0 mg, 80%) as a white solid. Compound **10**: ^{1}H NMR (300 MHz, CD_3OD) δ 7.78 (d, *J* = 1.4 Hz, 1H), 5.46 (s, 1H), 4.24 (s, 1H), 4.16 (s, 1H), 3.96 (d, *J* = 12.7 Hz, 1H), 3.88 (d, *J* = 12.7 Hz, 1H), 1.44–2.11 (m, 8H), 1.88 (d, *J* = 1.0 Hz, 3H); ^{13}C NMR (75.6 MHz, CD_3OD) δ 168.8, 153.6, 137.0, 110.6, 95.5, 91.3, 87.7, 81.6, 72.4, 57.5, 36.8, 36.3, 26.5, 24.4, 13.0; HRMS (MALDI) Calcd. for $C_{15}H_{20}N_2O_6Na$ $[M+Na]^+$: 347.1214, Found 347.1209.

*Synthesis of Compound **11***

To the solution of **10** (170 mg, 0.524 mmol) in dry pyridine (5.3 mL) was added 4,4′-dimethoxytrityl chloride (320 mg, 0.944 mmol) at 0 °C, and the reaction mixture was stirred at room temperature for 8 h. After addition of saturated aq. $NaHCO_3$, the resulting mixture was extracted with AcOEt. The organic layer was washed with water and brine, dried over Na_2SO_4, and concentrated. The crude product was purified by column chromatography (SiO_2, 1.0% triethylamine in *n*-hexane : AcOEt = 4 : 1 to 1 : 4) to afford **11** (329 mg, quant.) as a white solid. Compound **11**: ^{1}H NMR (300 MHz, $CDCl_3$) δ 8.94 (s, 1H), 7.67 (s, 1H), 7.48 (d, *J* = 6.9 Hz, 2H), 7.24–7.49 (m, 7H), 6.86 (d, *J* = 8.6 Hz, 4H), 5.56 (s, 1H), 4.39 (s, 1H), 4.29 (d, *J* = 3.8 Hz, 1H), 3.80 (s, 6H), 3.56 (d, *J* = 11.0 Hz, 1H), 3.49 (d, *J* = 11.0 Hz, 1H), 2.37 (d, *J* = 6.2 Hz, 1H), 1.96–2.09 (m, 1H), 1.45–1.93 (m, 6H), 1.68 (s, 3H), 1.26–1.37 (m, 1H); ^{13}C NMR (75.6 MHz, $CDCl_3$) δ 163.9, 158.6, 149.8, 144.4, 135.4, 135.3, 134.8, 130.0, 130.0, 128.0, 127.1, 113.3, 110.2, 94.2, 89.6, 86.9, 86.2, 80.0, 73.0, 58.6, 55.2, 35.9, 35.8, 25.7, 23.4, 12.5; HRMS (MALDI) Calcd. for $C_{36}H_{38}N_2O_8Na$ $[M+Na]^+$: 649.2520, Found 649.2526.

*Synthesis of compound **12***

To the solution of **11** (329 mg, 0.525 mmol) in dry dichloromethane (5.3 mL) was added *N*,*N*-diisopropylethylamine (275 µL, 1.61 mmol) and 2-cyanoethyl-*N,N*-diisopropylchlorophosphoramidite (175 µL, 0.784 mmol) at 0 °C. After stirring at room temperature for 8 h, the reaction mixture was concentrated and the crude product was purified by column chromatography (SiO_2, 1.0% triethylamine in *n*-hexane : AcOEt = 4 : 1 to 2 : 1) to afford **12** (252 mg, 58%) as a white solid. Compound **12**: ^{1}H NMR (300 MHz, $CDCl_3$) δ 8.37 (s, 1H), 7.73 (s, 1H), 7.46 (d, *J* = 7.2 Hz, 2H), 7.24–7.37 (m, 7H), 6.82–6.88 (m, 4H), 5.89 (s, 1/2H), 5.58 (s, 1/2H), 4.52 (d, *J* = 7.9 Hz, 1H), 4.37 (dd, *J* = 9.3, 16.1 Hz, 1H), 3.81 (s, 3H), 3.80 (s, 3H), 3.46–3.54 (m, 4H), 2.54–2.78 (m, 1H), 2.37 (dd, *J* = 5.9, 12.0 Hz, 1H), 1.93–2.07 (m, 1H), 1.60–1.89 (m, 4H), 1.65 (s, 3/2H), 1.63 (s, 3/2H), 1.41–1.55 (m, 2H), 1.25–1.29 (m, 3H), 1.16 (d, *J* = 6.5 Hz, 3H), 1.12 (d, *J* = 6.9 Hz, 3H), 1.07 (d, *J* = 6.5 Hz, 3H), 0.98 (d, *J* = 6.5 Hz, 3H); ^{31}P NMR (121.7 MHz, $CDCl_3$) δ 149.1, 148.7; HRMS (MALDI) Calcd. for $C_{45}H_{55}N_4O_9NaP$ $[M+Na]^+$: 849.3599, Found 849.3602.

*Synthesis of Compound **13***

To the solution of **11** (5.23 g, 8.35 mmol) in dry pyridine (80 mL) was added chlorotriethylsilane (3.60 mL, 24.0 mmol) at 0 °C under N_2 atmosphere. After the solution was stirred at room temperature for 7 h, saturated aq. $NaHCO_3$ was added at 0 °C, and the resulting mixture was extracted with AcOEt. The combined organic layer was washed with water and brine, dried over Na_2SO_4, and concentrated. The crude product was purified by column chromatography (SiO_2, *n*-hexane/AcOEt = 3 : 2 to 2 : 3) to afford **13** (5.52 g, 89%) as a white solid. Compound **13**: ^{1}H NMR (500 MHz, $CDCl_3$) δ 8.22 (s, 1H), 7.79 (d, *J* = 1.2 Hz, 1H), 7.46 (d, *J* = 7.5 Hz, 2H), 7.25–7.36 (m, 7H), 6.84 (d, *J* = 9.2 Hz, 2H), 6.84 (d, *J* = 9.2 Hz, 2H), 5.53 (s, 1H), 4.32 (s, 1H), 4.29 (s, 1H), 3.80 (s, 6H), 3.44 (d, *J* = 10.9 Hz, 1H), 3.37 (d, *J* = 10.9 Hz, 1H), 1.66 (s, 3H), 1.50–1.93 (m, 7H), 1.24–1.27 (m, 1H), 0.83–0.86 (m, 9H), 0.48–0.58 (m, 6H); ^{13}C NMR (125.8 MHz, $CDCl_3$) δ 163.9, 158.7, 149.7, 144.4, 135.5, 135.3, 135.0, 130.1, 130.0, 128.1, 128.0, 127.1, 113.2, 113.2, 110.2, 94.5, 89.6, 86.8, 86.7, 80.0, 73.0, 58.5, 55.2, 35.8, 35.6, 25.5, 23.4, 12.6, 6.6, 4.7; HRMS (MALDI) calcd for $C_{42}H_{52}N_2O_8NaSi$ $[M+Na]^+$: 763.3391, found 763.3369.

*Synthesis of Compound **14***

To the solution of **13** (329 mg, 444 µmol) and triethylamine (1.1 mL, 7.9 mmol) in dry acetonitrile (4.8 mL) were added 1,2,4-triazole (537 mg, 7.77 mmol) and phosphoryl chloride (136 µL, 1.45 mmol) at 0 °C. After the mixture was stirred at room temperature for 1 h, saturated aq. $NaHCO_3$ was added, and the resulting mixture was extracted with AcOEt. The combined organic layer was washed with water and brine, dried over Na_2SO_4, and concentrated. The crude was used immediately for the next reaction without further purification. To a solution of the crude in 1,4-dioxane (2.0 mL) was added aq. ammonia (28 wt %, 500 µL, 7.3 mmol) at 0 °C, and the reaction mixture was stirred at room temperature for 1.5 h. After completion of the reaction, the resulting mixture was concentrated. The crude product was purified by column chromatography (SiO_2, $CHCl_3$/MeOH = 20:1) to afford **14** (322 mg, 98%, two steps) as a white solid. Compound **14**: ^{1}H NMR (500 MHz, $CDCl_3$) δ 7.84 (s, 1H), 7.48 (d, *J* = 7.5 Hz, 2H), 7.22–7.38 (m, 7H), 6.85 (d, *J* = 8.9 Hz, 2H), 6.84 (d, *J* = 8.9 Hz, 2H), 5.60 (s, 1H), 4.38 (s, 1H), 4.30 (s, 1H), 3.80 (s, 6H), 3.44 (d,

J = 10.9 Hz, 1H), 3.36 (d, J = 10.9 Hz, 1H), 1.68 (s, 3H), 1.43–1.92 (m, 7H), 1.22–1.26 (m, 1H), 0.81–0.84 (m, 9H), 0.45–0.55 (m, 6H); ^{13}C NMR (125.8 MHz, $CDCl_3$) δ 165.6, 158.6, 155.5, 144.6, 138.0, 135.6, 135.5, 130.1, 130.0, 128.1, 127.9, 127.0, 113.2, 113.1, 101.0, 94.2, 89.2, 87.3, 86.6, 80.0, 72.9, 58.7, 55.2, 35.8, 35.5, 25.6, 23.4, 13.2, 6.6, 4.7; HRMS (MALDI) calcd for $C_{42}H_{53}N_3O_7NaSi$ $[M+Na]^+$: 762.3550, found 762.3565.

*Synthesis of Compound **15***

To the solution of **14** (322 mg, 435 μmol) in dry pyridine (4.5 mL) was added benzoyl chloride (100 μL, 861 μmol) at 0 °C under N_2 atmosphere, and the reaction mixture was stirred at room temperature for 2.5 h. After addition of saturated aq. $NaHCO_3$, the resulting mixture was extracted with AcOEt. The combined organic layer was washed with water and brine, dried over Na_2SO_4, and concentrated. The crude product was purified by column chromatography (SiO_2, *n*-hexane/AcOEt = 5:1) to afford **15** (229 mg, 62%) as a white solid. Compound **15**: ^{1}H NMR (500 MHz, $CDCl_3$) δ 8.32 (d, J = 7.5 Hz, 2H), 7.97 (s, 1H), 7.42–7.53 (m, 5H), 7.26–7.38 (m, 7H), 6.84–6.87 (m, 4H), 5.59 (s, 1H), 4.34 (s, 1H), 4.34 (s, 1H), 3.81 (s, 6H), 3.47 (d, J = 10.9 Hz, 1H), 3.39 (d, J = 10.3 Hz, 1H), 1.45–1.95 (m, 7H), 1.56 (s, 3H), 1.24–1.29 (m, 1H), 0.83–0.86 (m, 9H), 0.48–0.58 (m, 6H); ^{13}C NMR (75.6 MHz, $CDCl_3$) δ 179.4, 159.7, 158.6, 147.4, 144.3, 137.0, 136.1, 135.3, 135.2, 132.3, 130.0, 129.9, 129.7, 128.0, 127.9, 127.0, 113.1, 113.1, 111.2, 94.4, 89.6, 87.0, 86.6, 79.8, 72.8, 58.4, 55.1, 35.7, 35.4, 25.4, 23.3, 13.6, 6.5, 4.5; HRMS (MALDI) calcd for $C_{49}H_{57}N_3O_8NaSi$ $[M+Na]^+$ 866.3813, found 866.3809.

*Synthesis of Compound **16***

To the solution of **15** (108 mg, 121 μmol) in tetrahydrofuran (1.2 mL) was added 1 M tetrabutylammonium fluoride in tetrahydrofuran (120 μL, 120 μmol) at 0 °C, and the reaction mixture was stirred at room temperature for 20 min. After completion of reaction, the reaction mixture was concentrated. The crude product was purified by column chromatography ($CHCl_3$/MeOH = 20 : 1) to afford **16** (101 mg, quant.) as a white solid. Compound **16**: ^{1}H NMR (300 MHz, $CDCl_3$) δ 8.32 (d, J = 7.2 Hz, 2H), 7.85 (s, 1H), 7.24–7.56 (m, 12H), 6.88 (d, J = 8.9 Hz, 4H), 5.61 (s, 1H), 4.40 (s, 1H), 4.30 (d, J = 7.2Hz, 1H), 3.82 (s, 6H), 3.55 (s, 2H), 1.89 (s, 3H), 1.60–2.06 (m, 7H), 1.25–1.34 (m, 1H); ^{13}C NMR (75.6 MHz, $CDCl_3$) δ 179.4, 159.7, 158.5, 147.5, 144.4, 136.8, 136.2, 135.3, 135.3, 132.4, 129.9, 129.7, 127.9, 127.0, 113.2, 111.2, 94.1, 89.6, 86.8, 86.5, 79.8, 72.6, 58.5, 55.1, 35.8, 35.6, 25.6, 23.3, 13.5; HRMS (MALDI) calcd for $C_{43}H_{43}N_3O_8Na$ $[M+Na]^+$ 752.2948, found 752.2939.

*Synthesis of Compound **17***

To the solution of **16** (71.0 mg, 97.3 μmol) in dry acetonitrile (980 μL) were added 4,5-dicyanoimidazole (35.0 mg, 296 μmol) and 2-cyanoethyl *N,N,N',N'*-tetraisopropylphosphorodiamidite (95.0 μL, 299 μmol) at 0 °C under N_2 atmosphere. After the solution was stirred at room temperature for 2 h, the resulting mixture was concentrated. The crude product was purified by column chromatography (SiO_2, 1% triethylamine in *n*-hexane/AcOEt = 3:1) to afford **17** (73.0 mg, 81%) as a white solid. Compound **17**: ^{1}H NMR (500 MHz, $CDCl_3$) δ 8.31 (d, J = 7.5 Hz, 2H) , 7.91 (s,

3/4H), 7.89 (s, 1/4H), 7.28–7.52 (m, 12H), 6.84–6.89 (m, 4H), 5.63 (s, 1H), 4.58 (s, 1/4H), 4.56 (s, 3/4H), 4.42 (d, *J* = 9.2 Hz, 3/4H), 4.38 (d, *J* = 7.4 Hz, 1/4H), 3.82 (s, 4H), 3.81 (s, 2H), 3.47–3.56 (m, 6H), 2.51–2.63 (m, 1/2H), 2.34–2.37 (m, 3/2H), 1.83 (s, 1H), 1.77 (s, 2H), 1.26–2.05 (m, 8H), 1.15 (d, *J* = 6.9 Hz, 4H), 1.12 (d, *J* = 6.9 Hz, 2H), 1.07 (d, *J* = 6.3 Hz, 4H), 0.98 (d, *J* = 6.9 Hz, 2H); ^{31}P NMR (161.8 MHz, $CDCl_3$) δ 149.4, 148.9; HRMS (MALDI) calcd for $C_{52}H_{60}N_5O_9NaP$ $[M+Na]^+$ 952.4021, found 952.4005.

3. ^{1}H NMR, ^{13}C NMR and ^{31}P NMR spectra of novel compounds

Compound **2** (^{1}H-NMR, $CDCl_3$, 300 MHz)

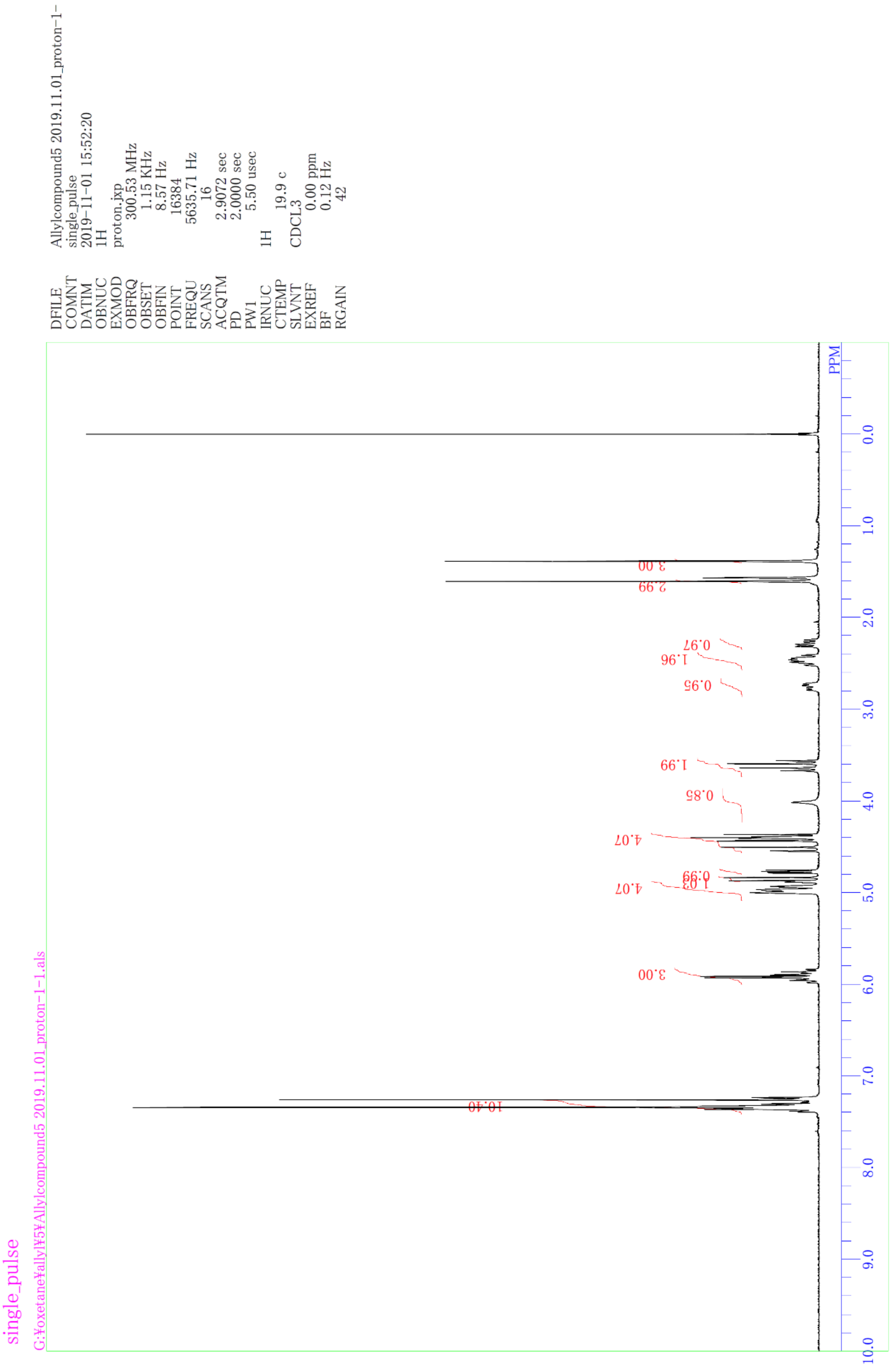

Compound **2** (^{13}C NMR, $CDCl_3$, 75.6 MHz)

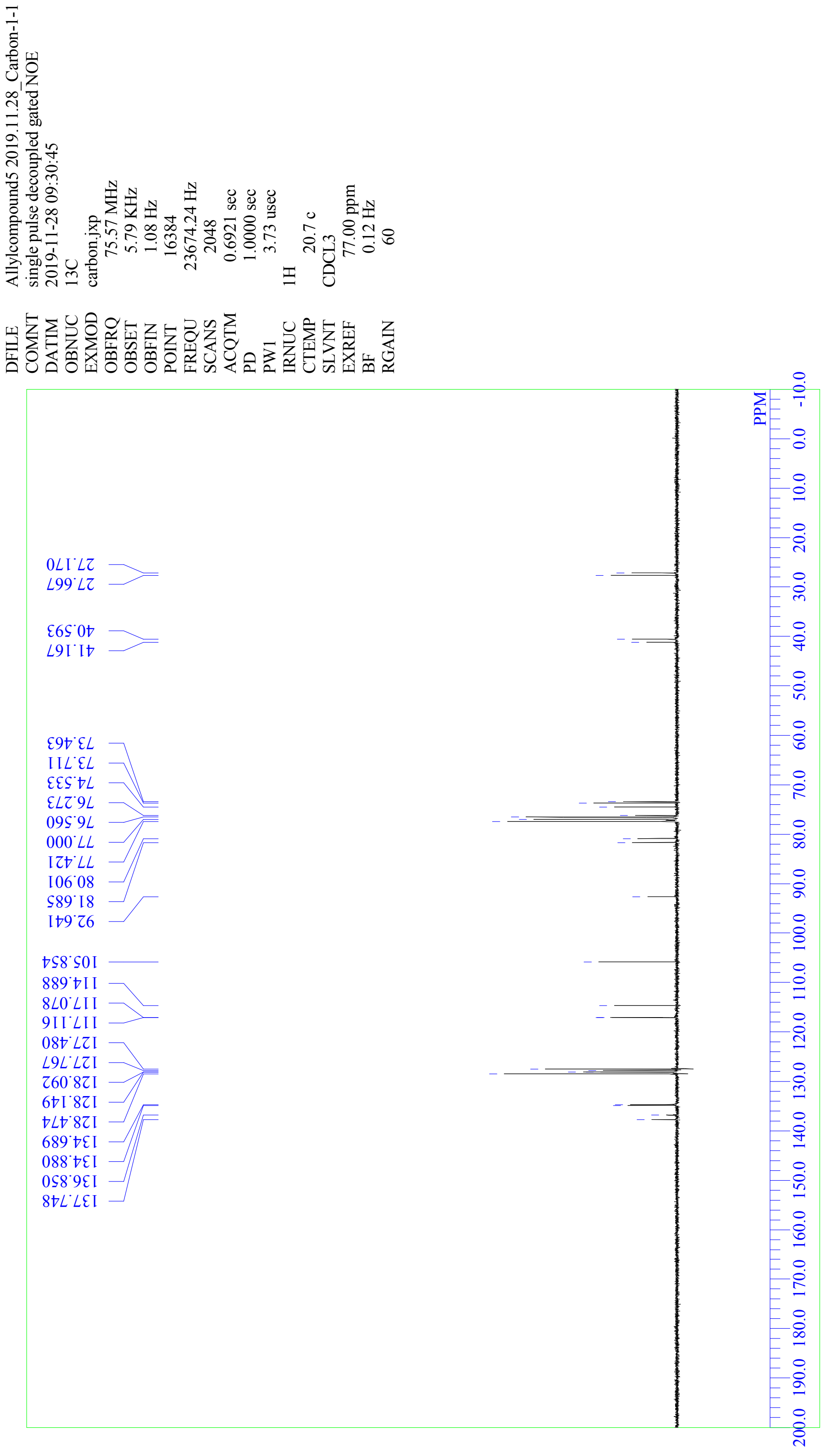

Compound **3** (^{1}H NMR, $CDCl_3$, 300 MHz)

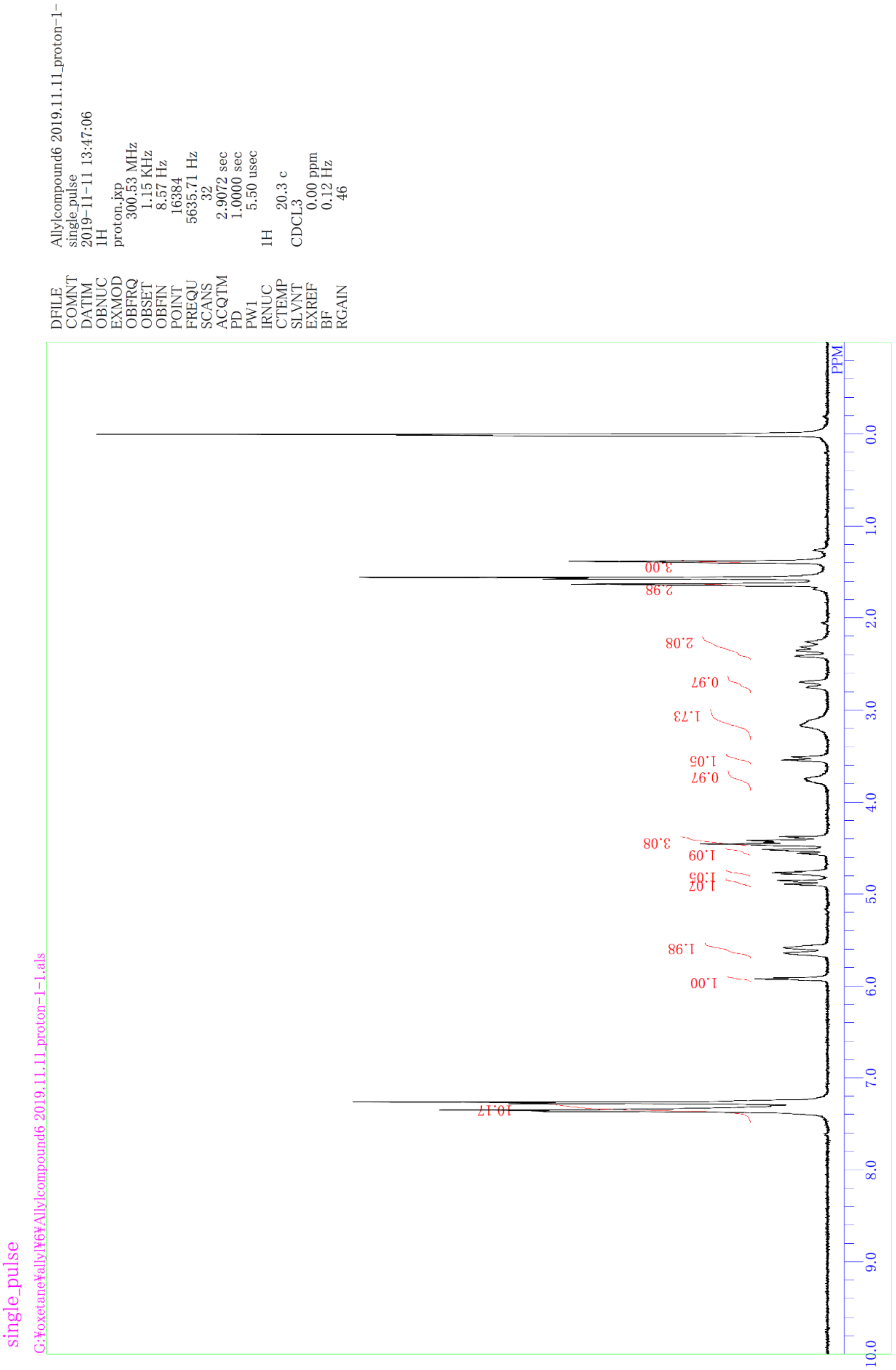

Compound **3** (^{13}C NMR, $CDCl_3$, 75.6 MHz)

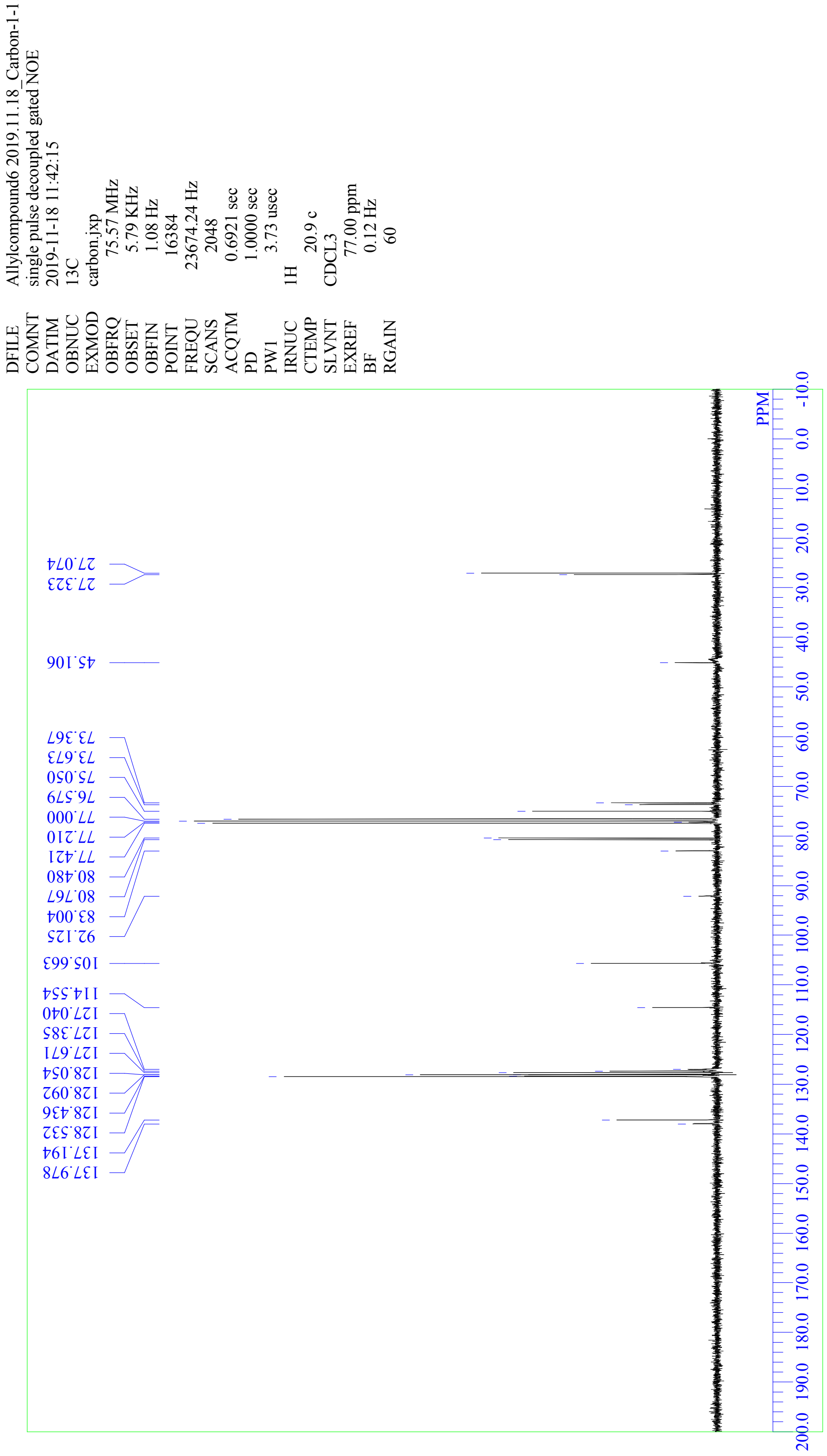

Compound **4** (^{1}H-NMR, $CDCl_3$, 300 MHz)

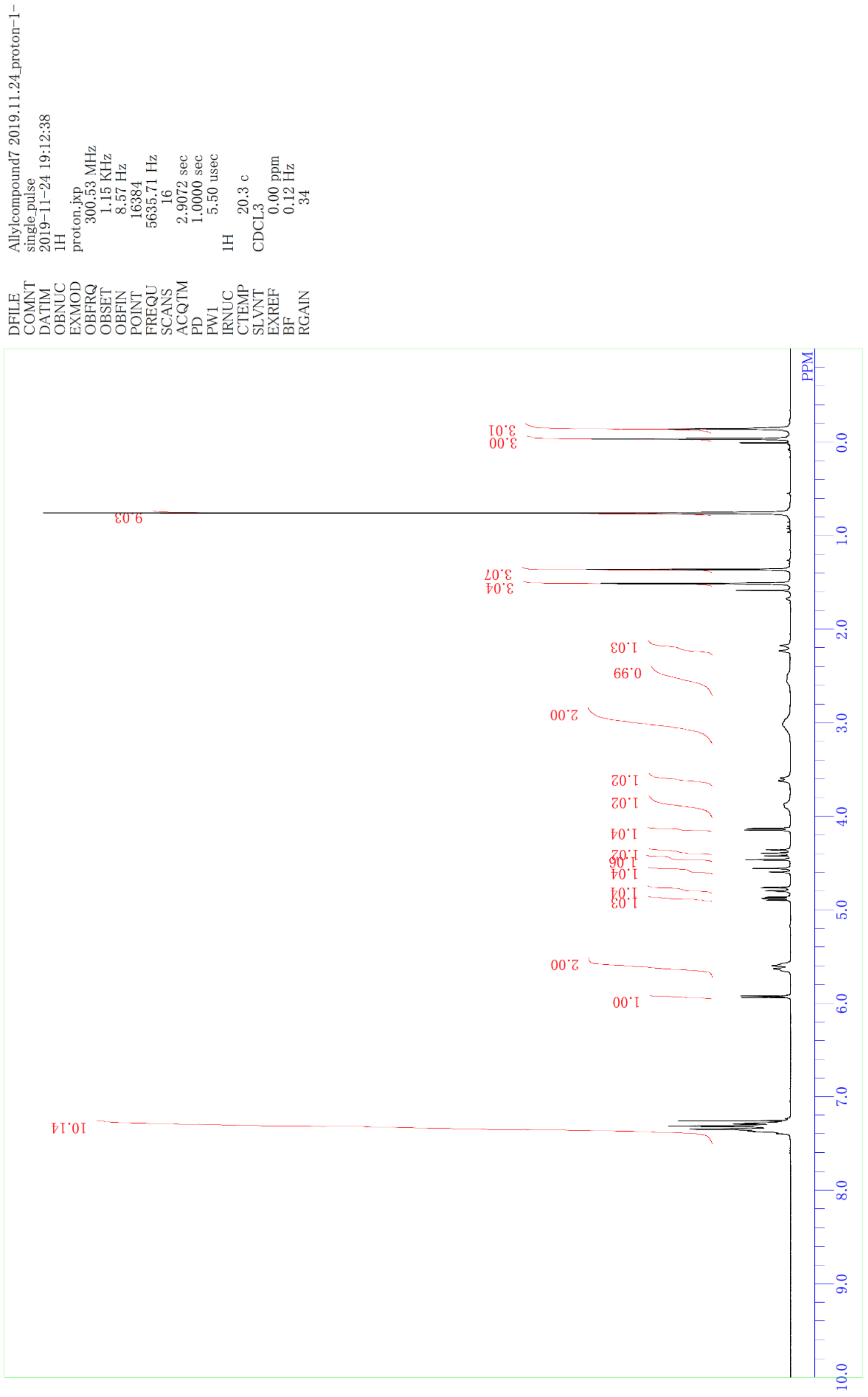

Compound **4** (^{13}C NMR, $CDCl_3$, 75.6 MHz)

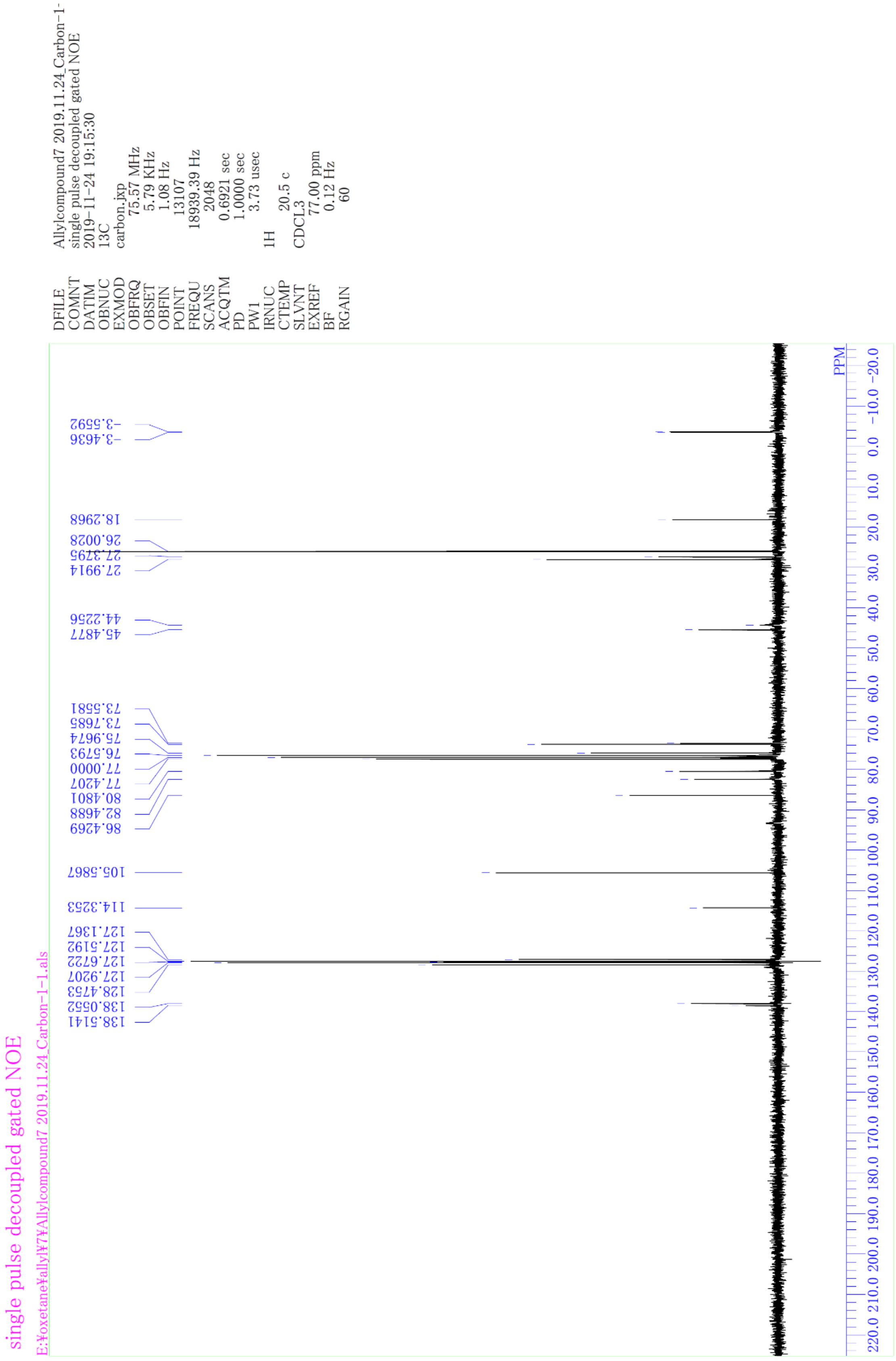

Compound **5** (^{1}H-NMR, $CDCl_3$, 300 MHz)

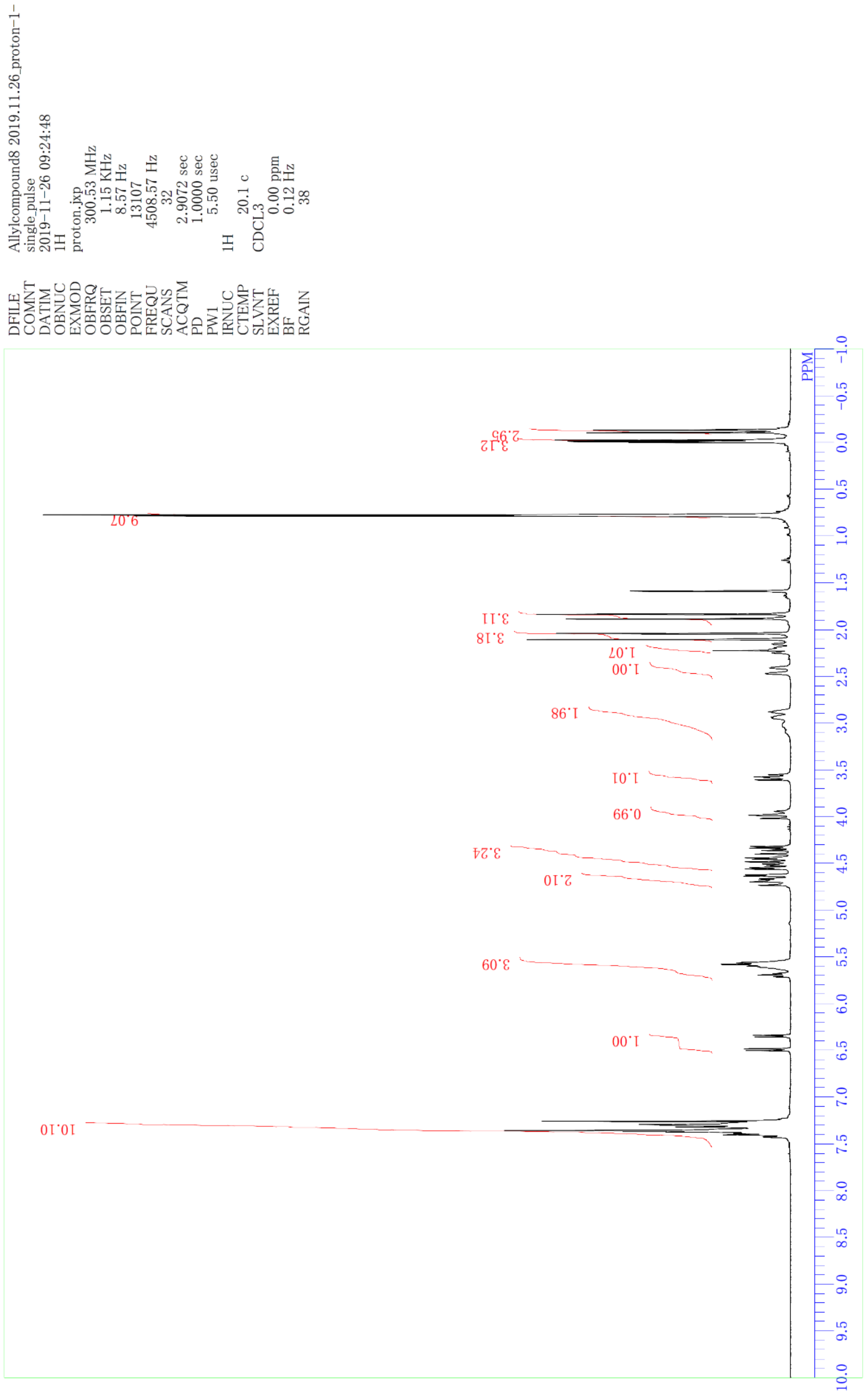

Compound **6** (^{1}H-NMR, $CDCl_3$, 300 MHz)

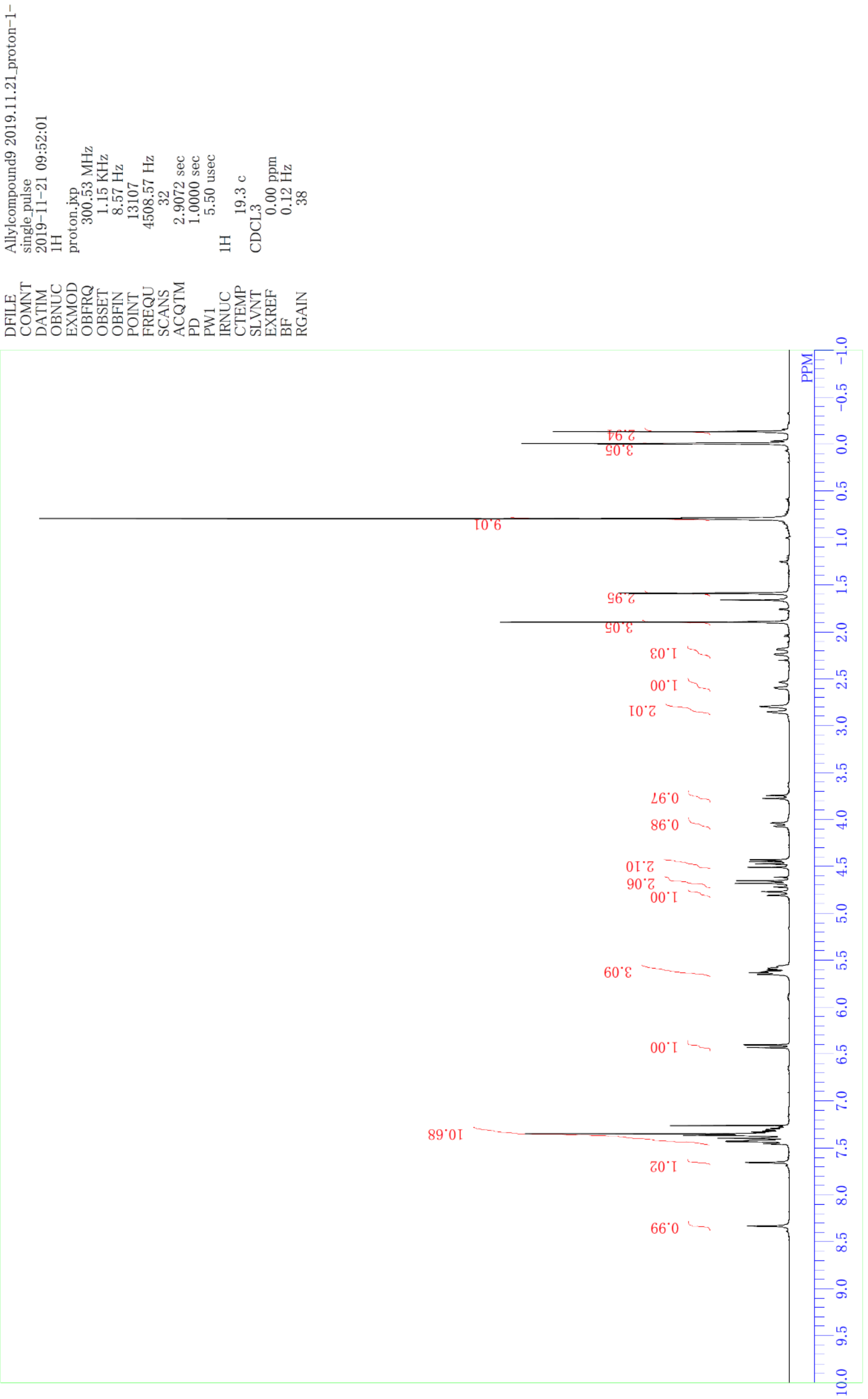

Compound **6** (^{13}C NMR, $CDCl_3$, 75.6 MHz)

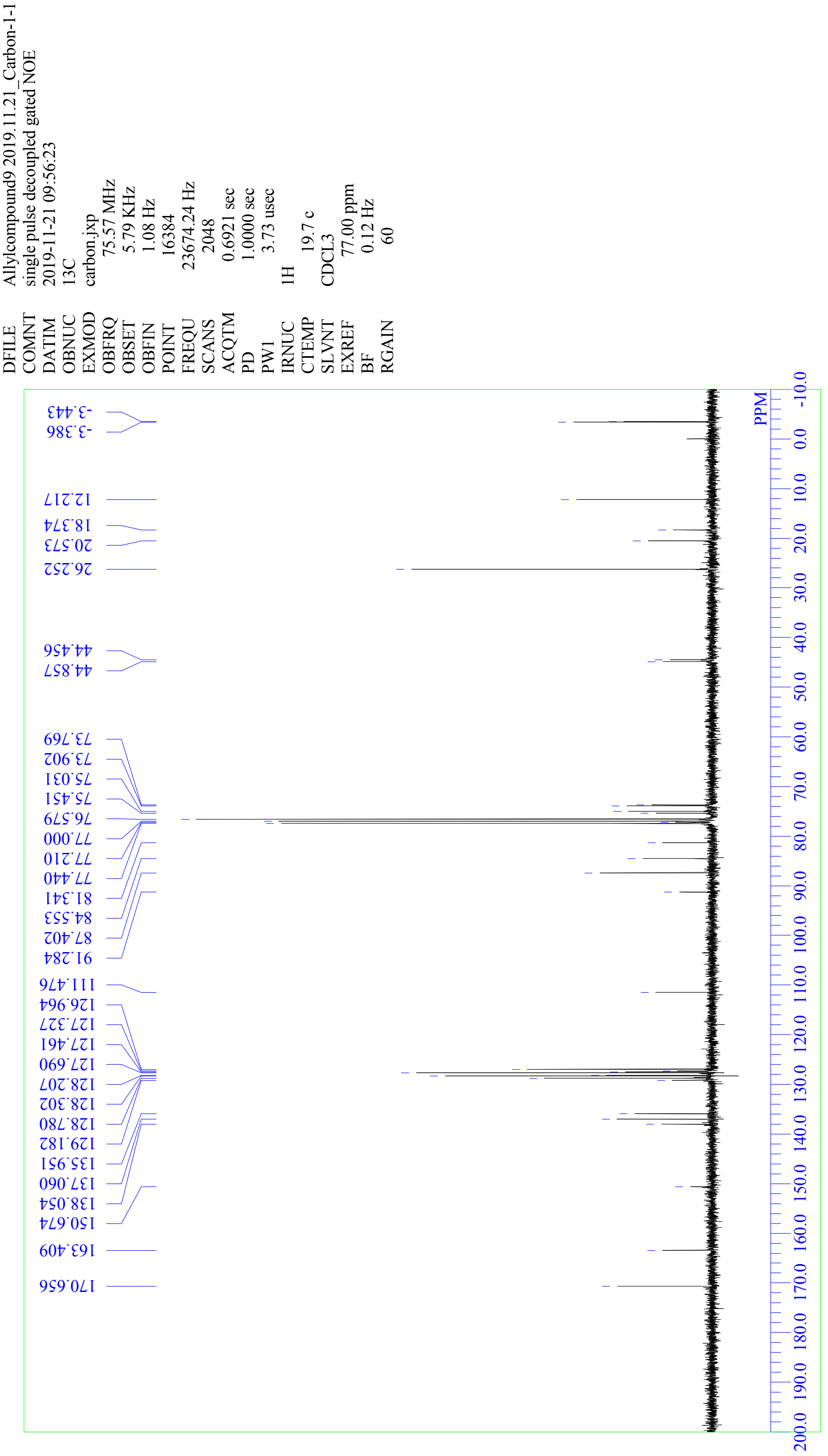

Compound **7** (^{1}H-NMR, $CDCl_3$, 300 MHz)

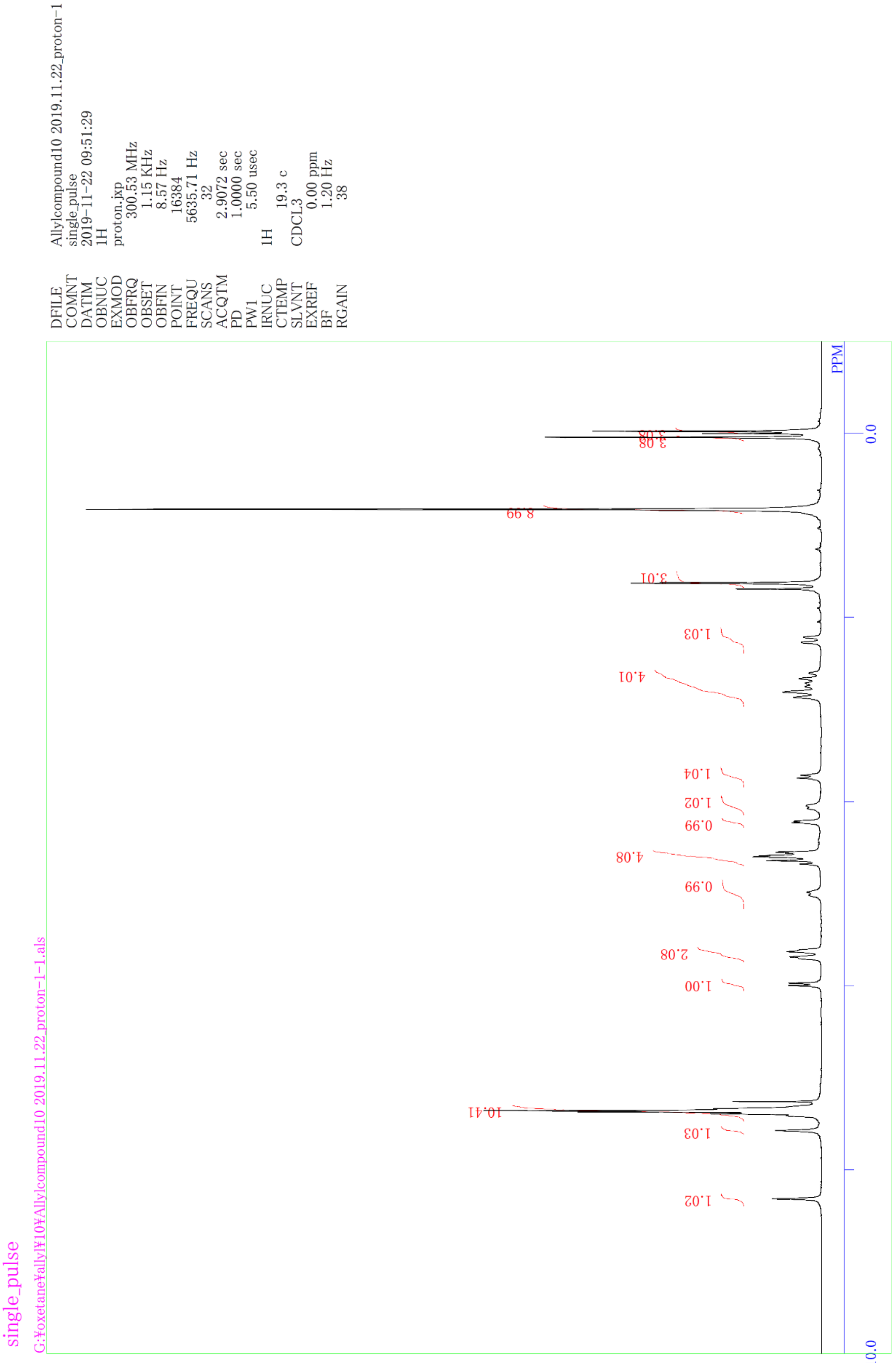

Compound **7** (^{13}C NMR, $CDCl_3$, 75.6 MHz)

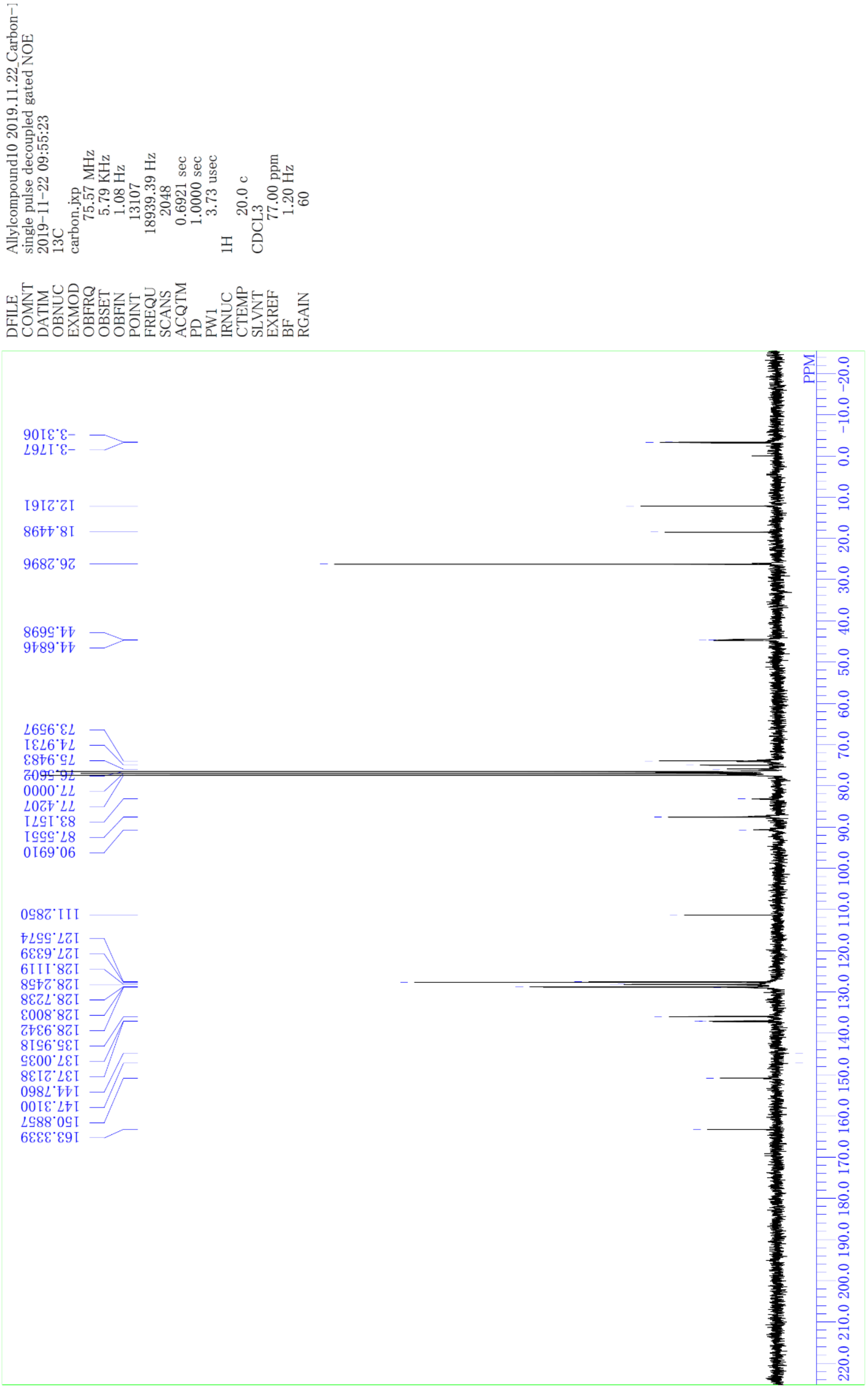

Compound **8** (^{1}H-NMR, $CDCl_3$, 300 MHz)

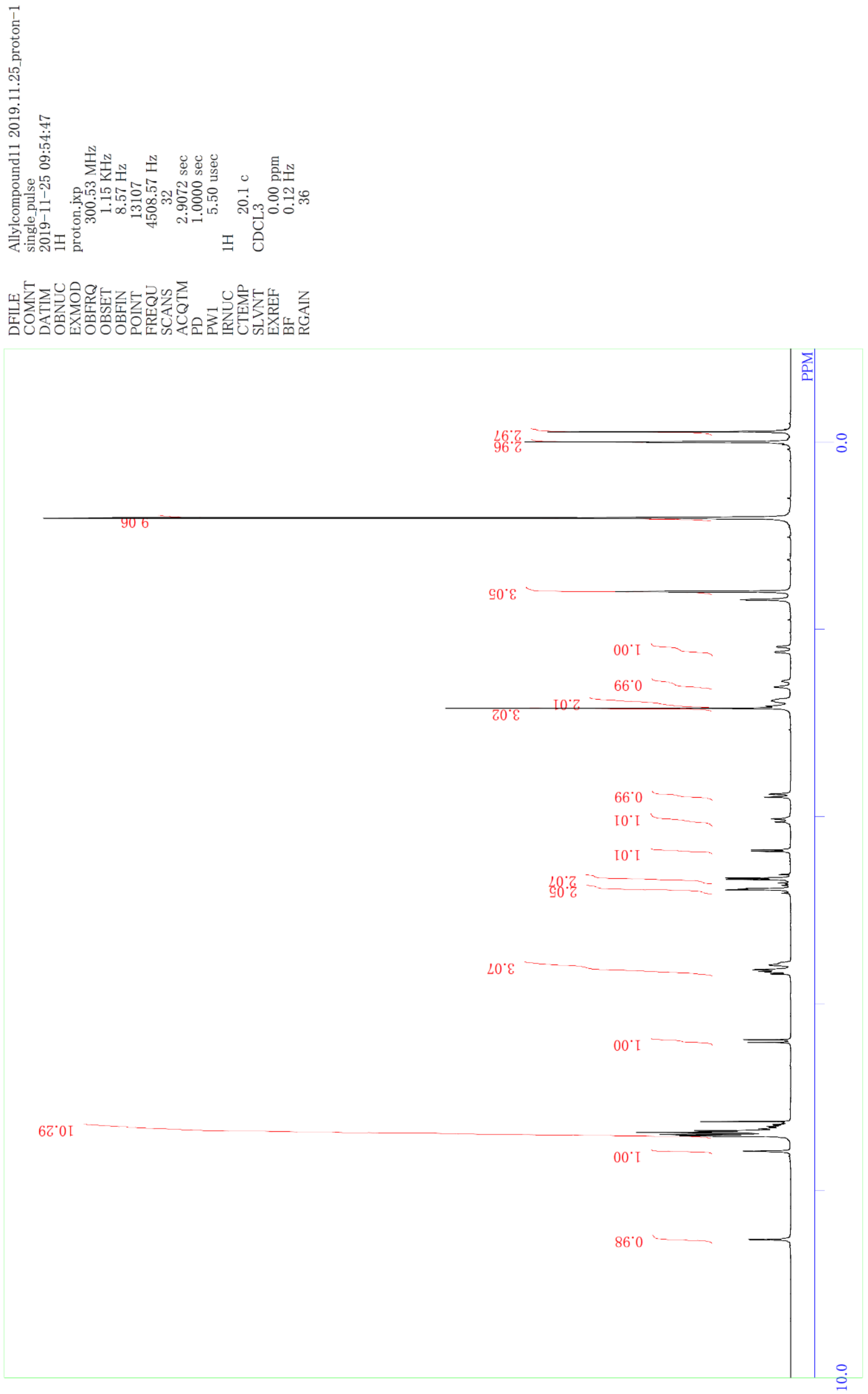

Compound **8** (^{13}C NMR, $CDCl_3$, 75.6 MHz)

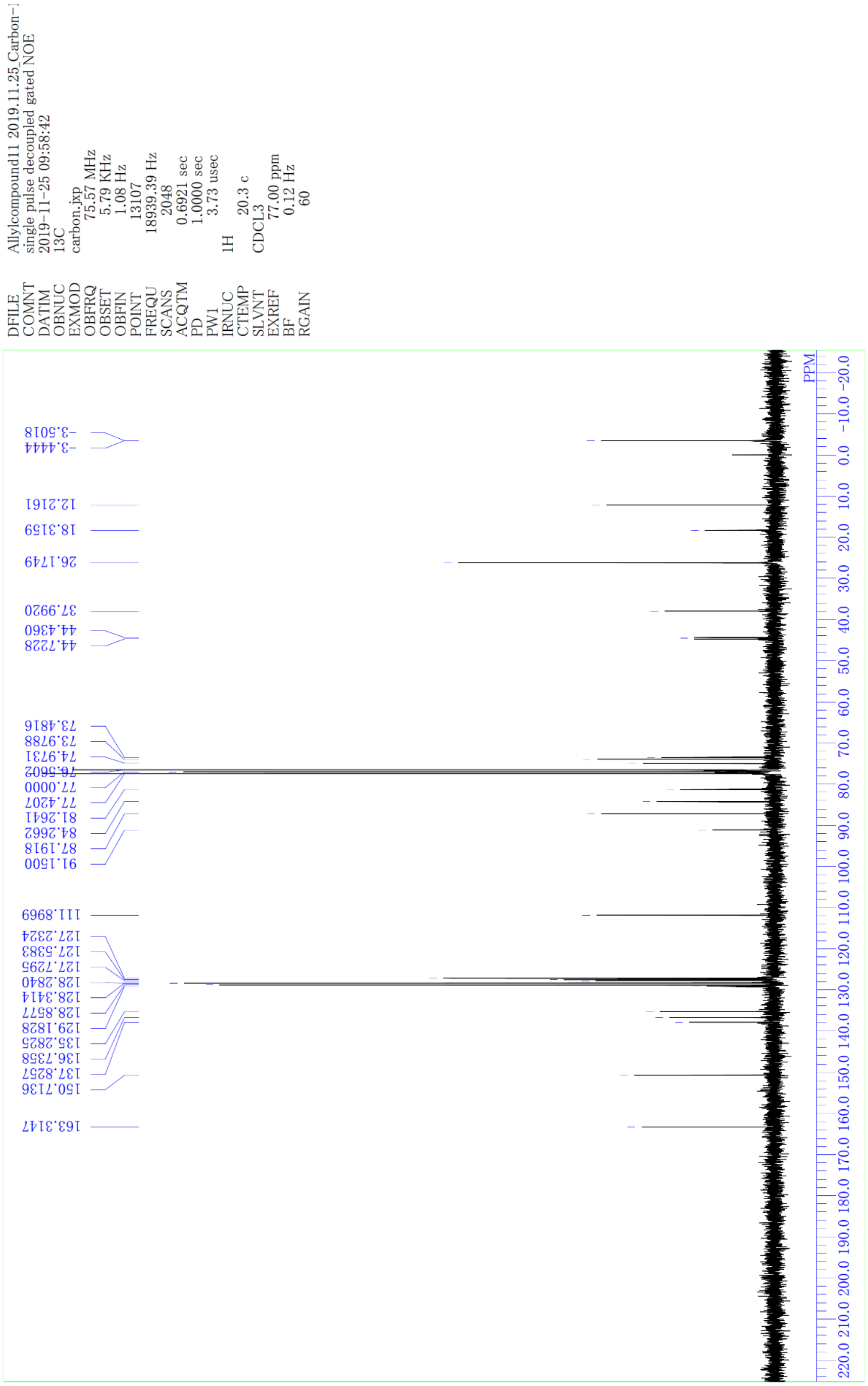

Compound **9** (^{1}H-NMR, $CDCl_3$, 300 MHz)

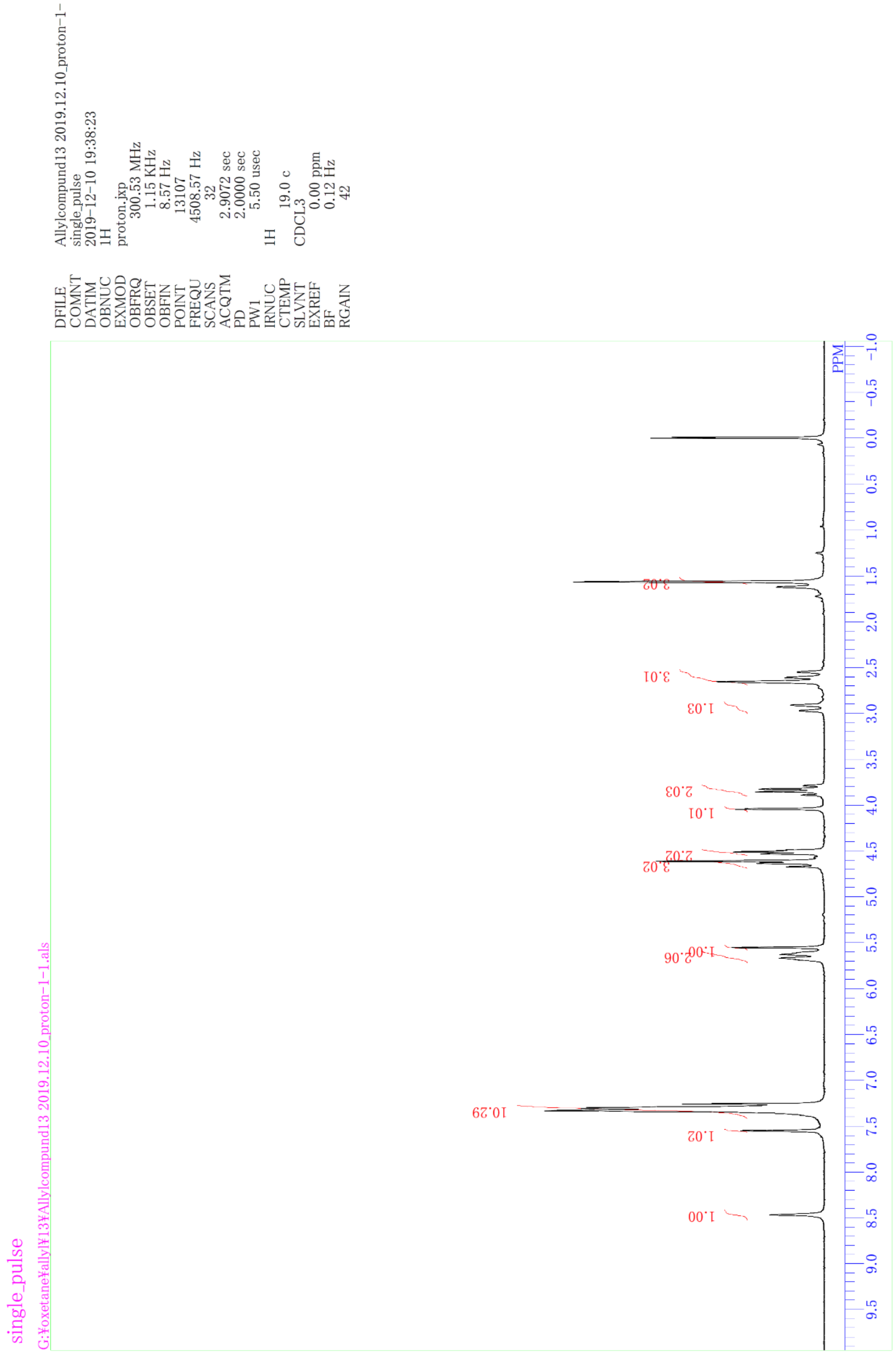

Compound **9** (^{13}C NMR, $CDCl_3$, 75.6 MHz)

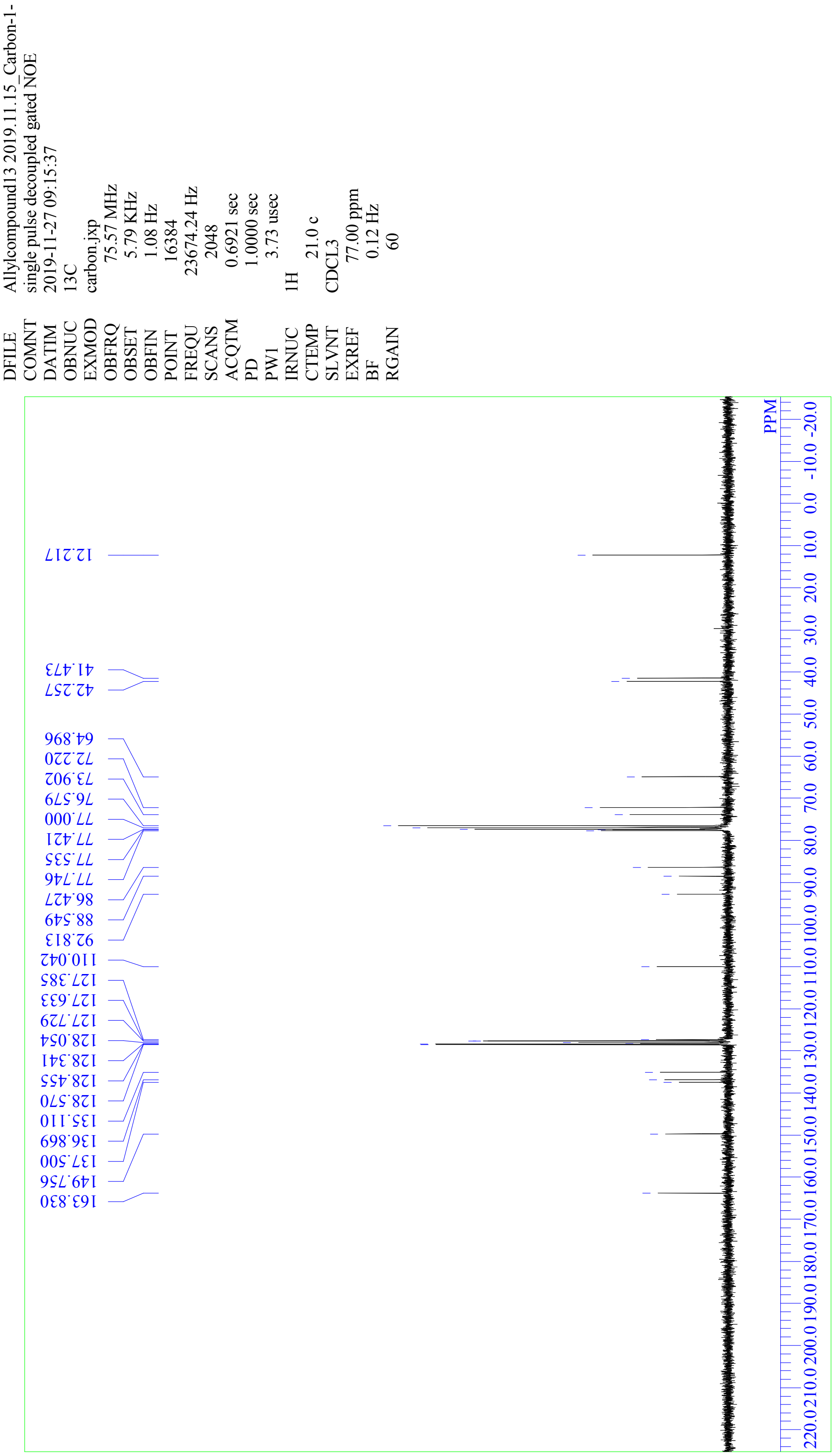

Compound **10** (^{1}H-NMR, MeOH-d_4, 300 MHz)

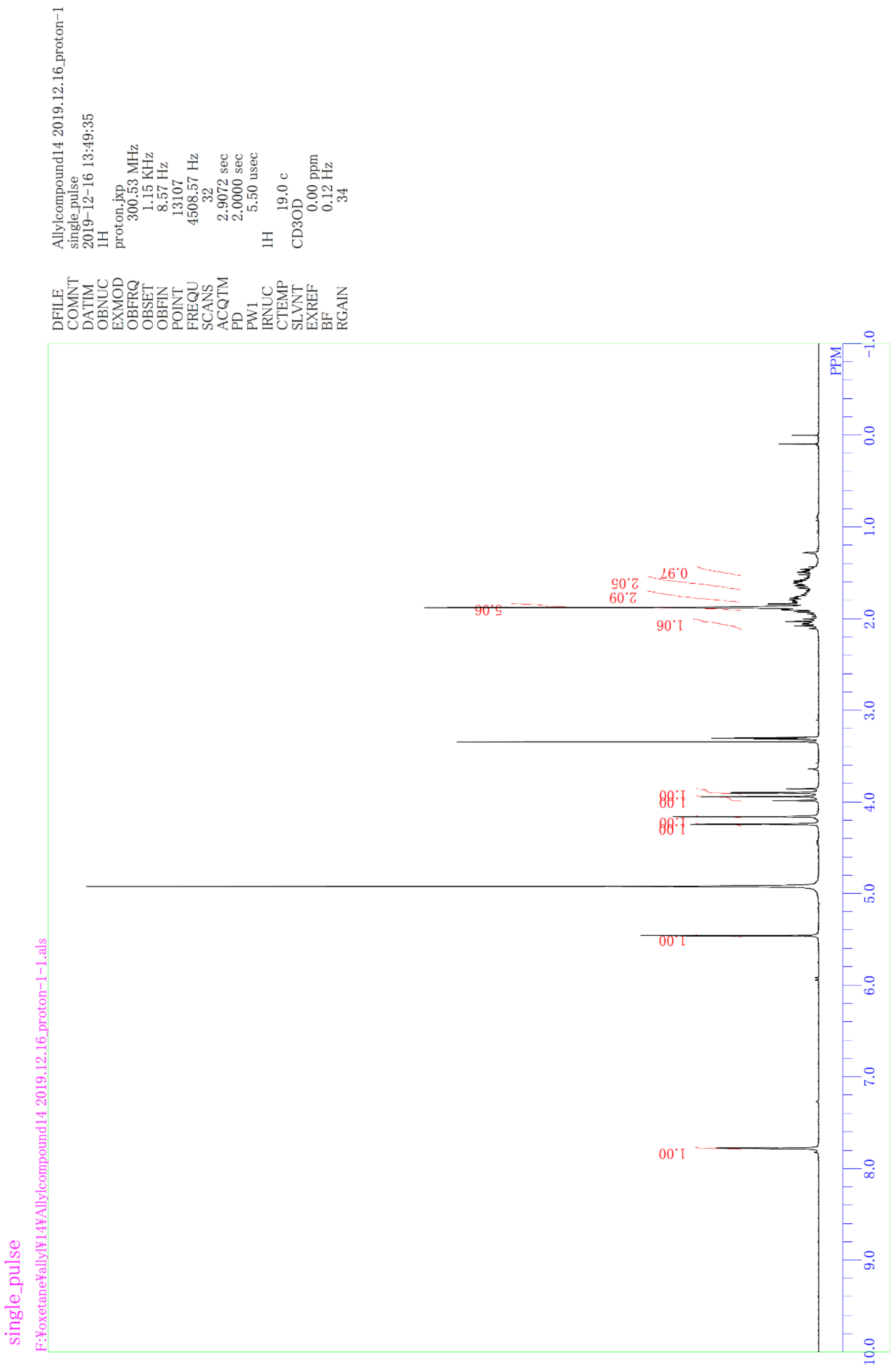

Compound **10** (^{13}C NMR, MeOH-d_4, 75.6 MHz)

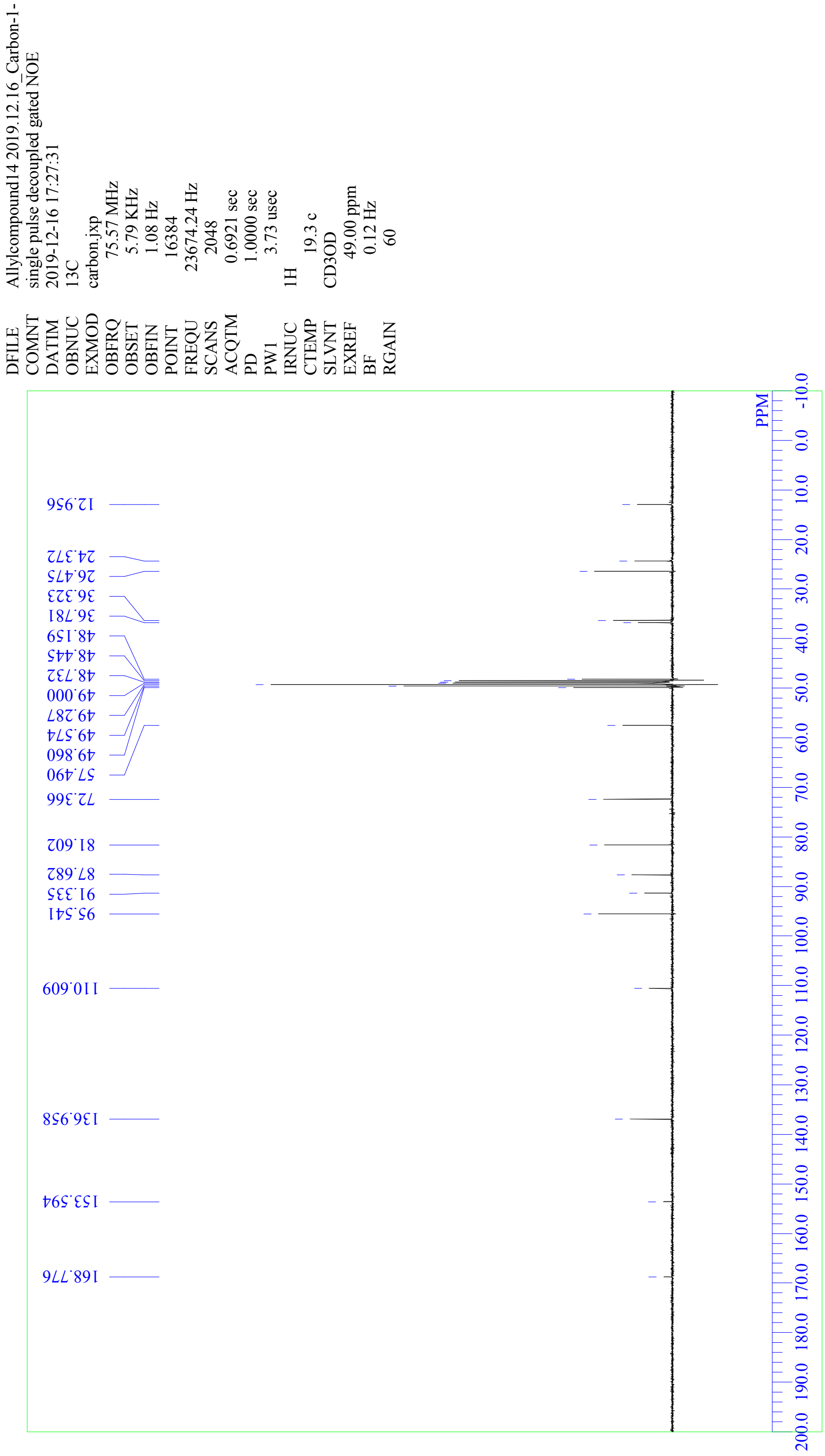

Compound **11** (^{1}H-NMR, $CDCl_3$, 300 MHz)

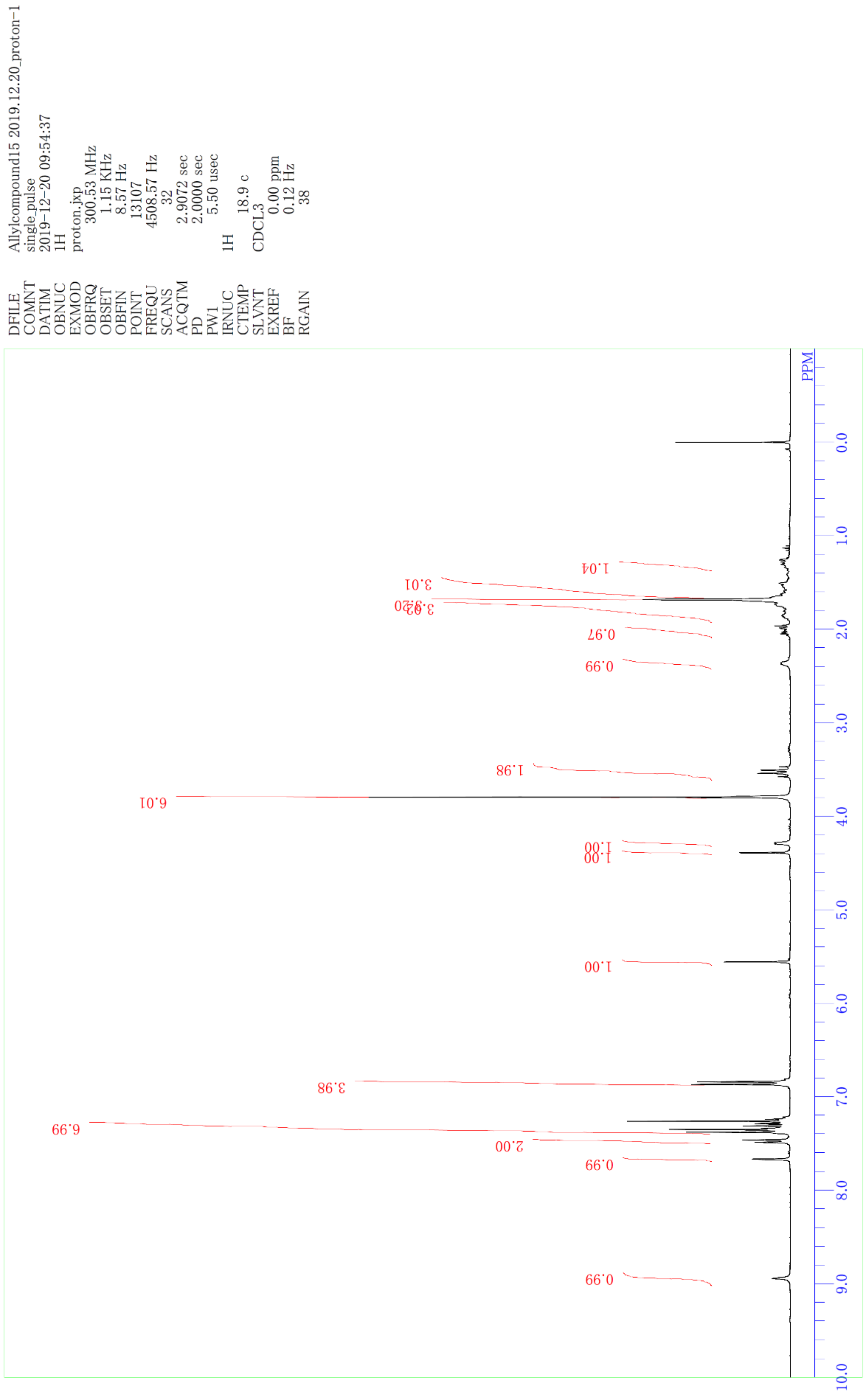

Compound **11** (^{13}C NMR, $CDCl_3$, 75.6 MHz)

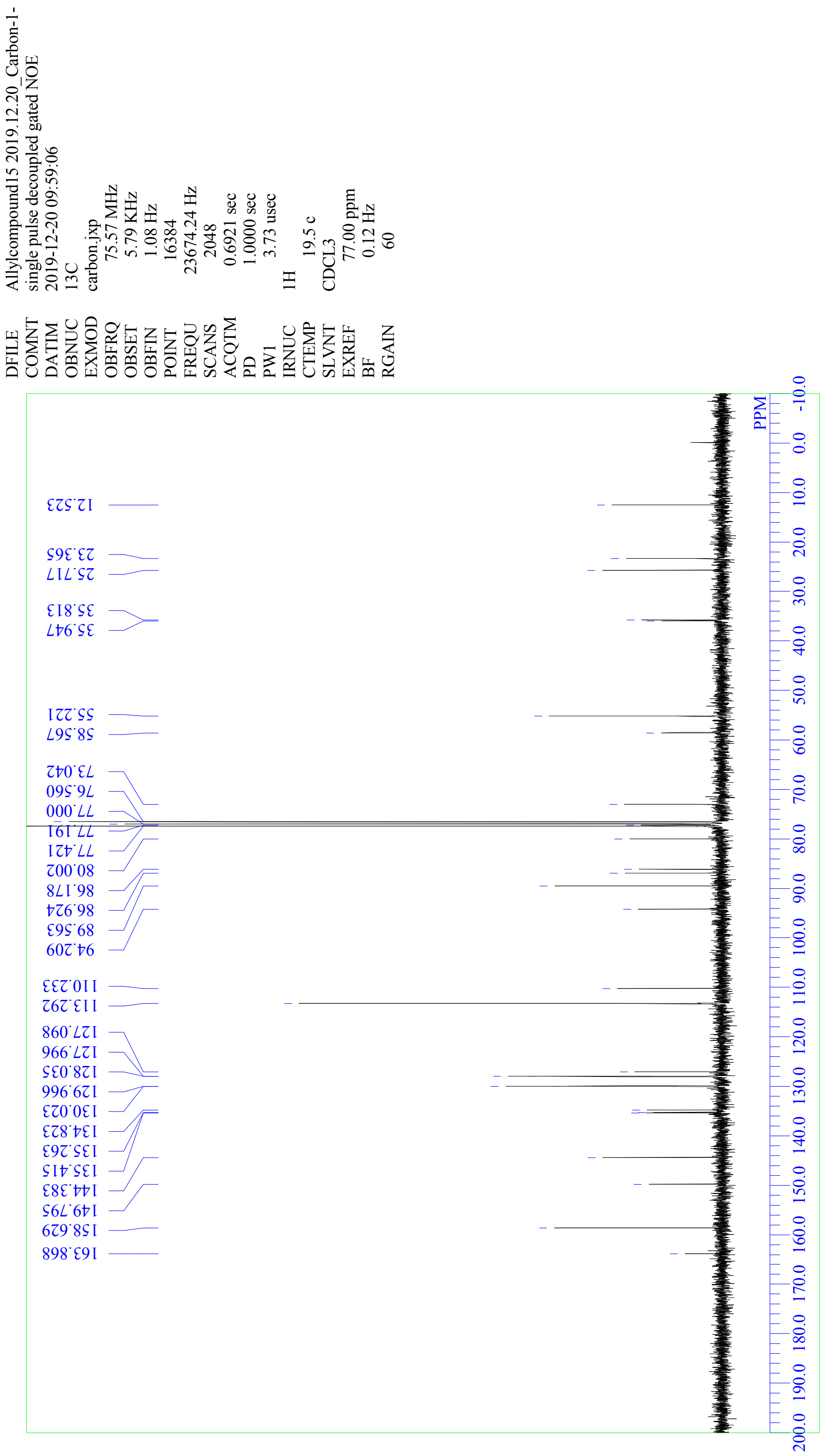

Compound **12** (^{1}H-NMR, $CDCl_3$, 300 MHz)

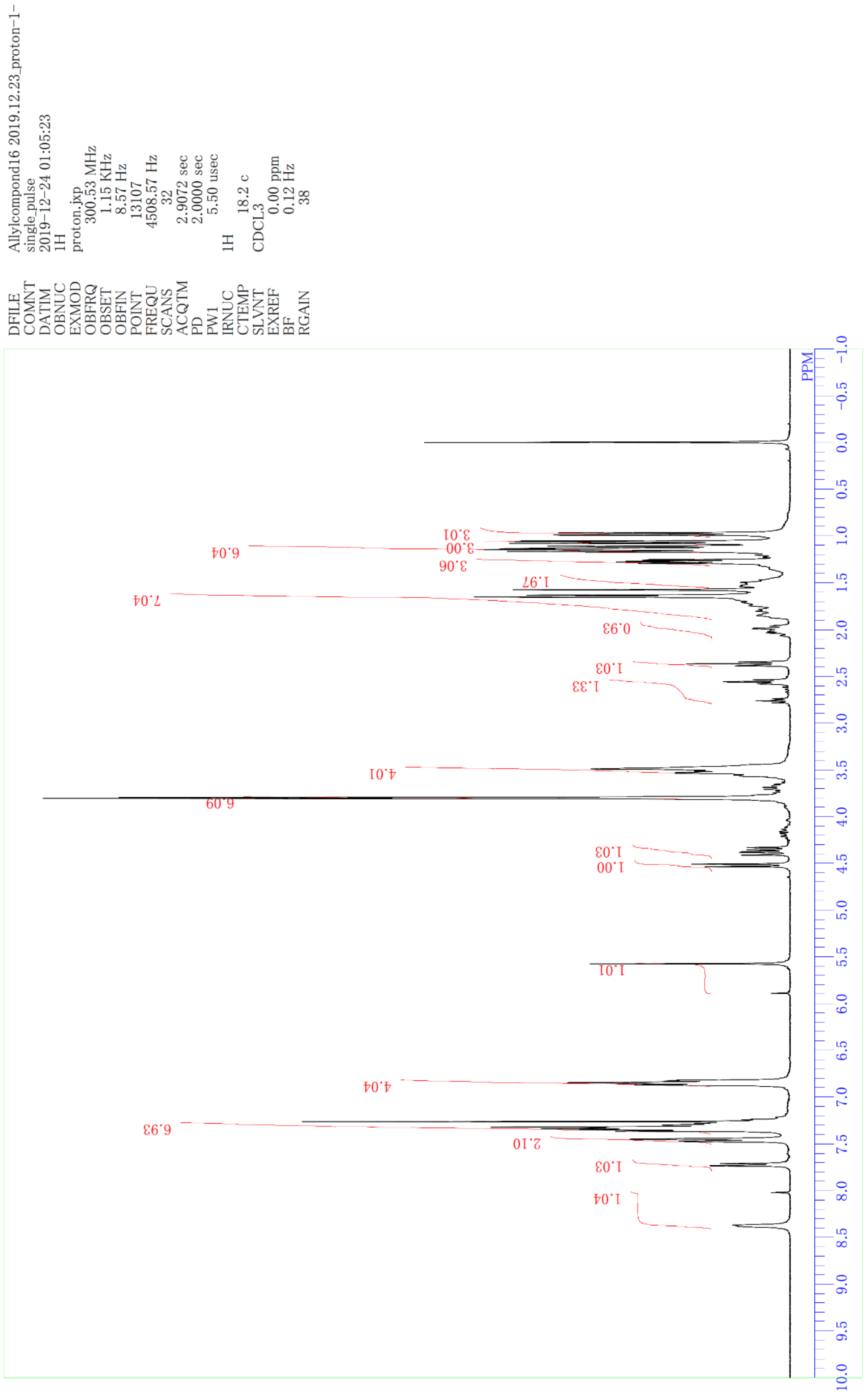

Compound **12** (^{31}P-NMR, $CDCl_3$, 121.7 MHz)

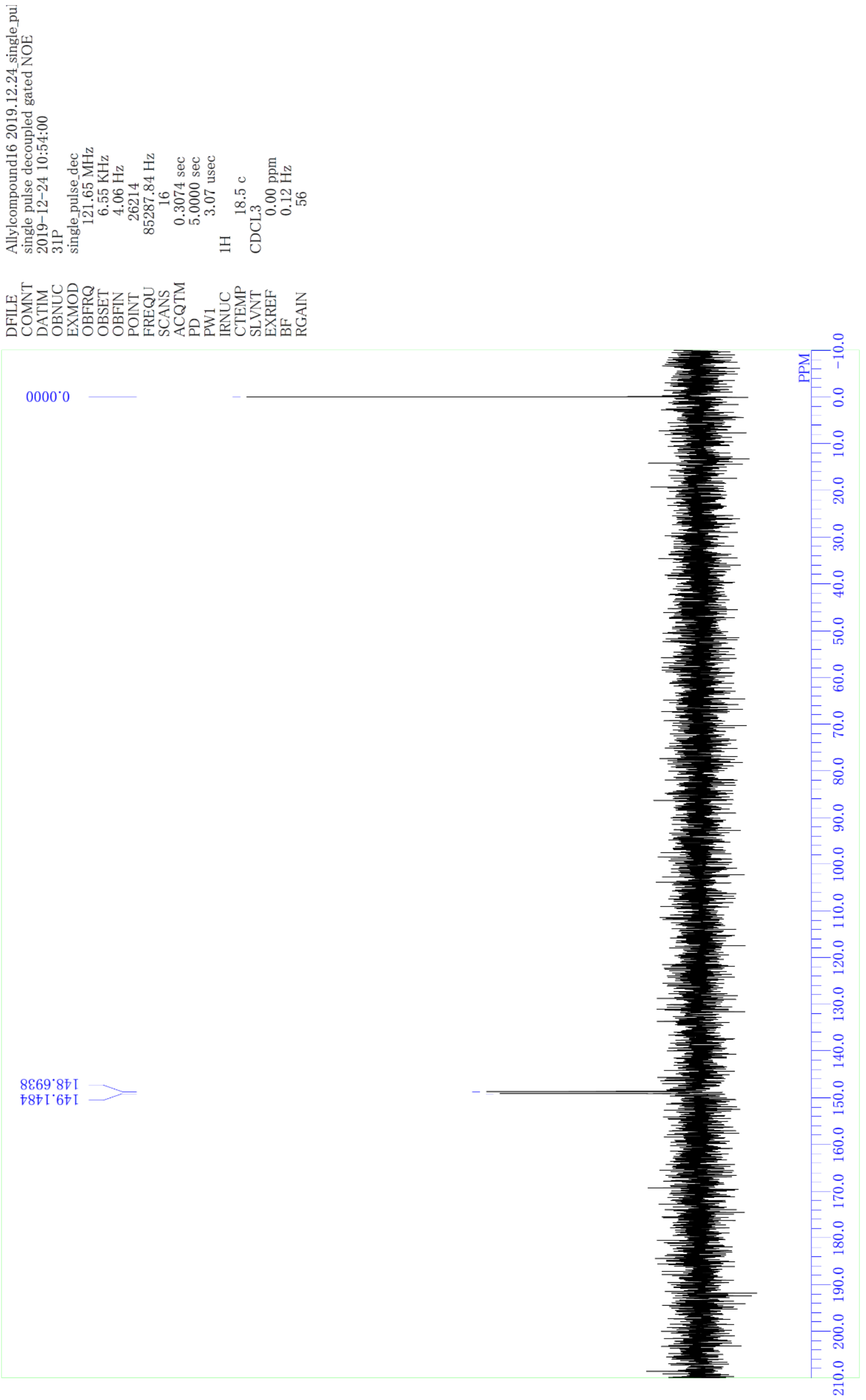

Compound **13** (^{1}H-NMR, $CDCl_3$, 500 MHz)

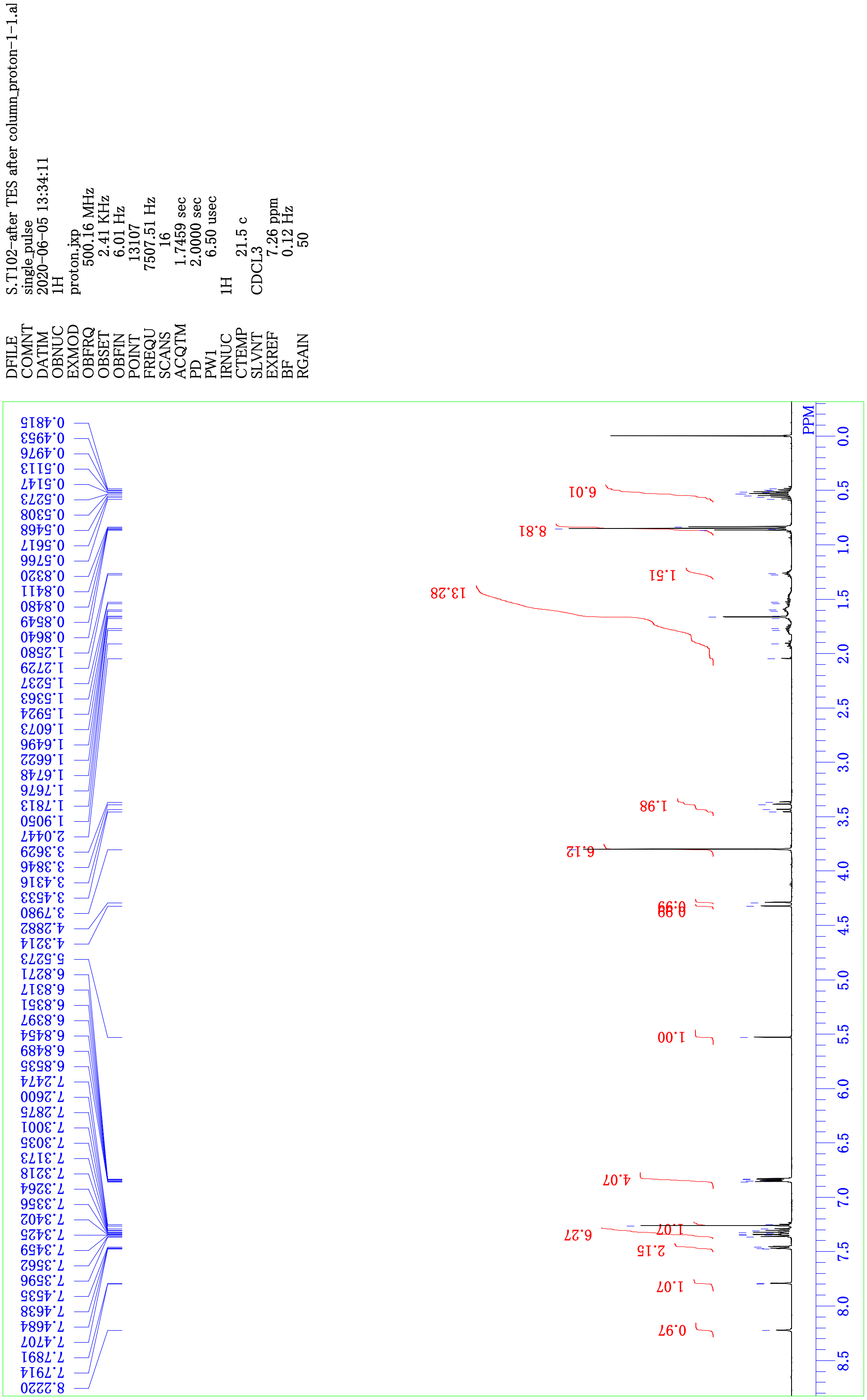

Compound **13** (^{13}C NMR, $CDCl_3$, 125.8 MHz)

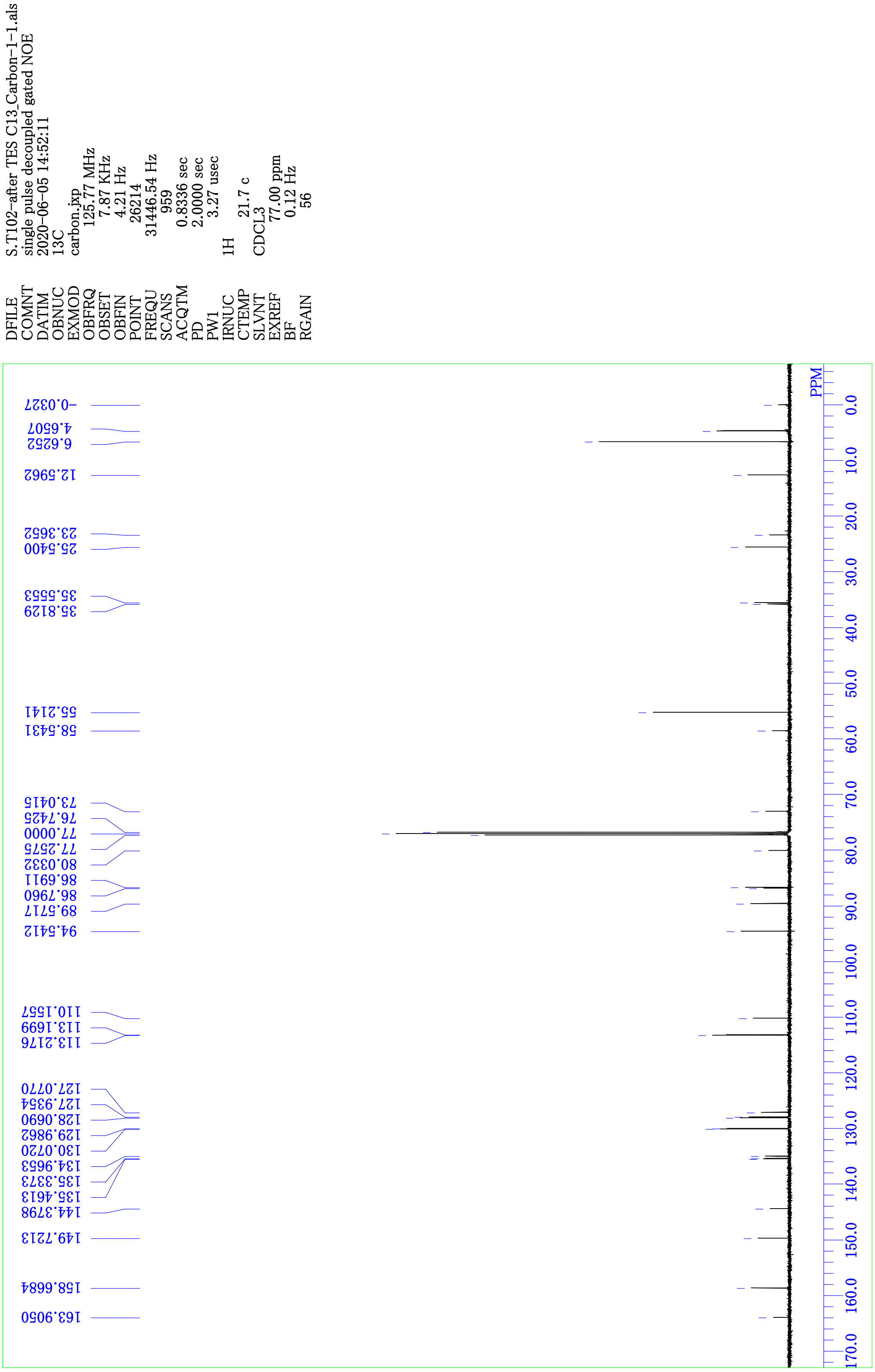

Compound **14** (^{1}H-NMR, $CDCl_3$, 500 MHz)

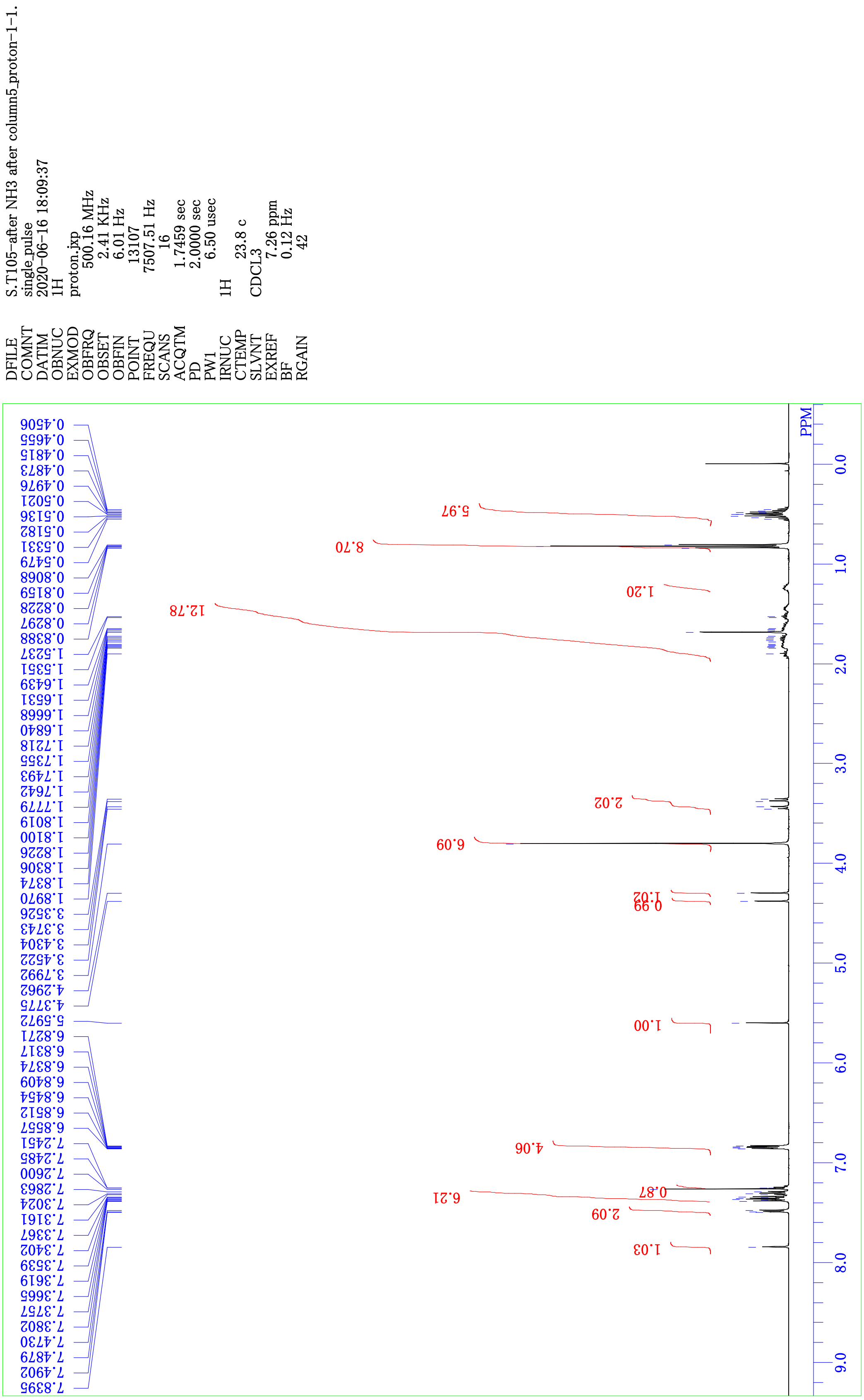

Compound **14** (^{13}C NMR, $CDCl_3$, 125.8 MHz)

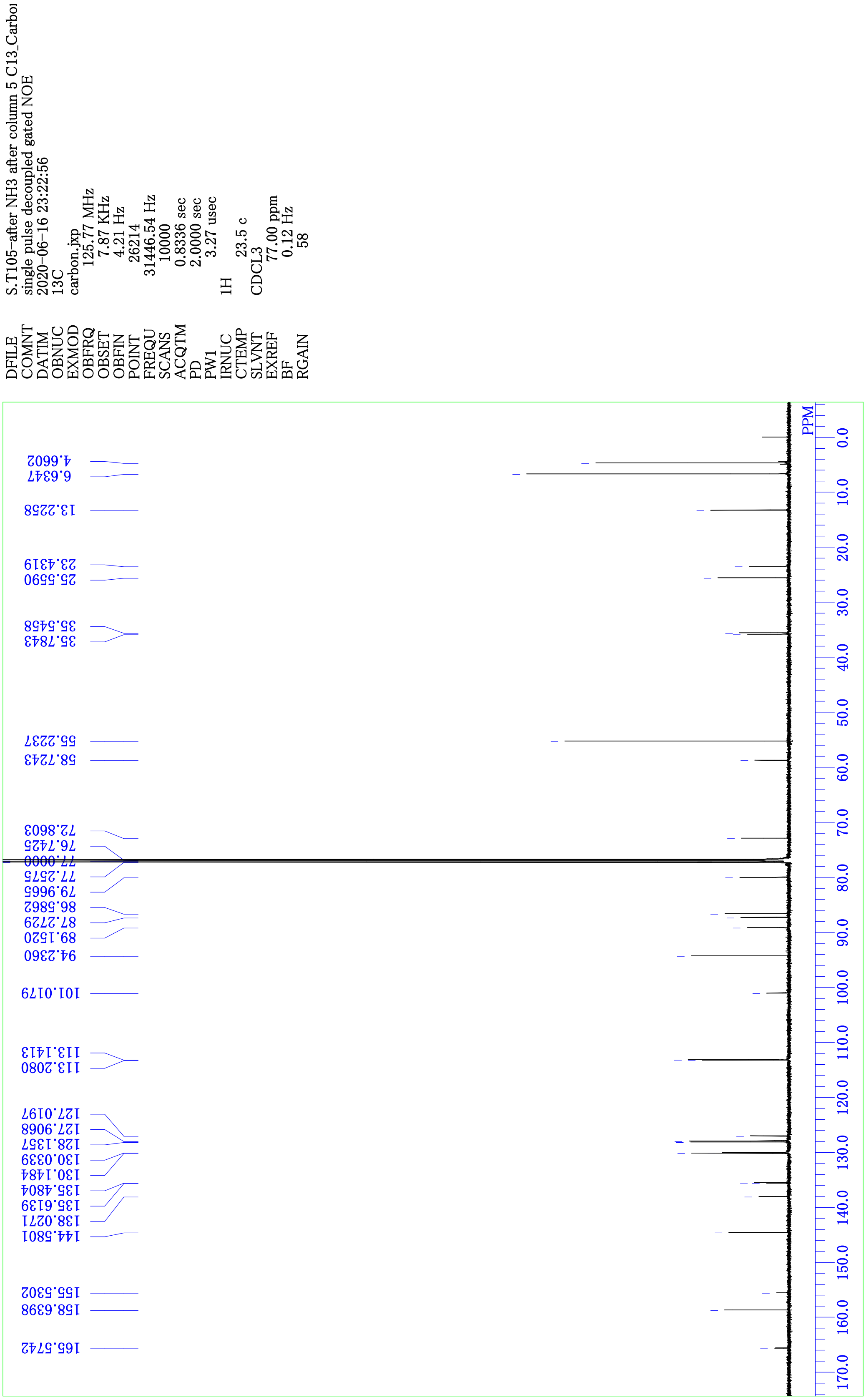

Compound **15** (^{1}H-NMR, $CDCl_3$, 500 MHz)

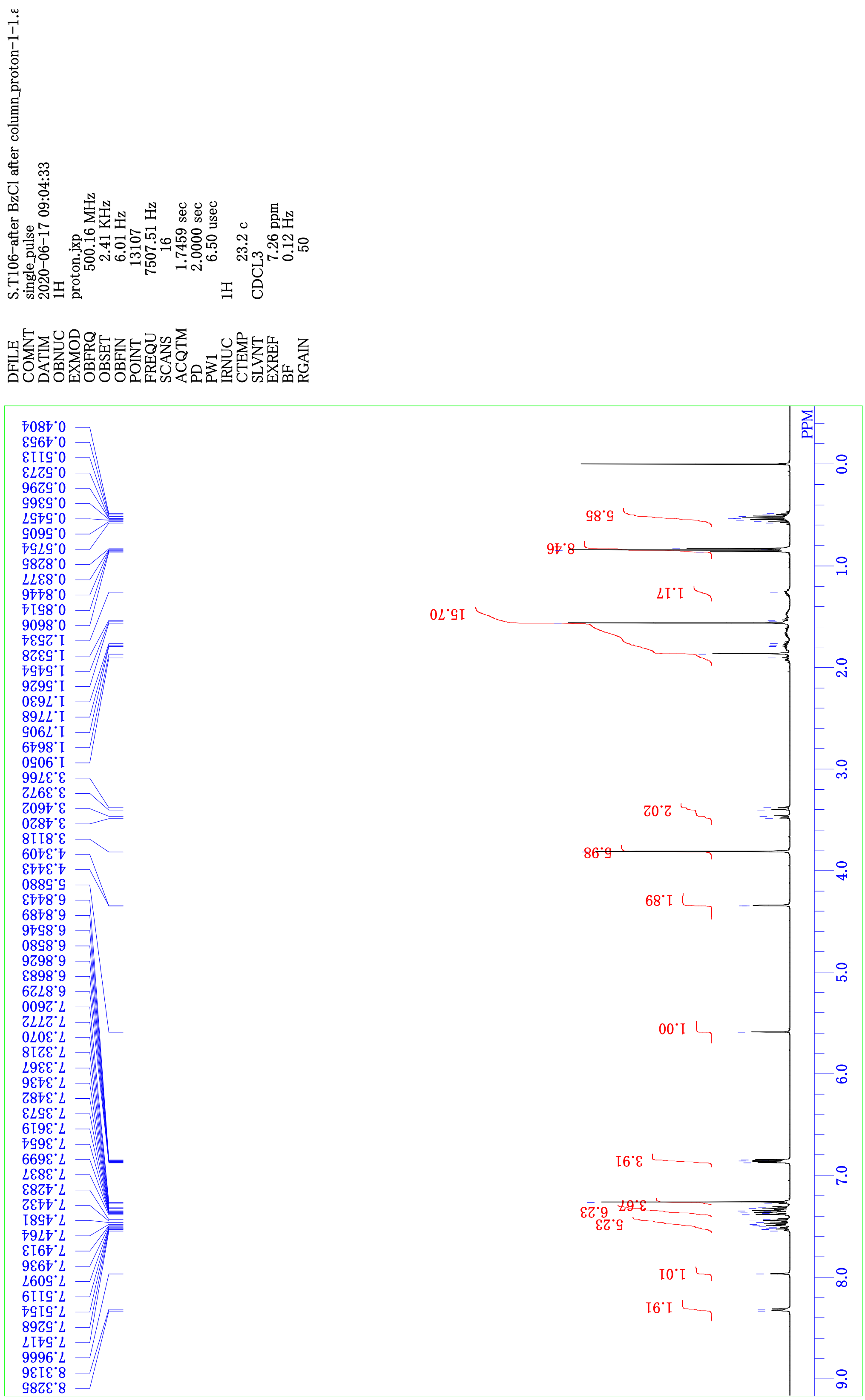

Compound **15** (^{13}C NMR, $CDCl_3$, 75.6 MHz)

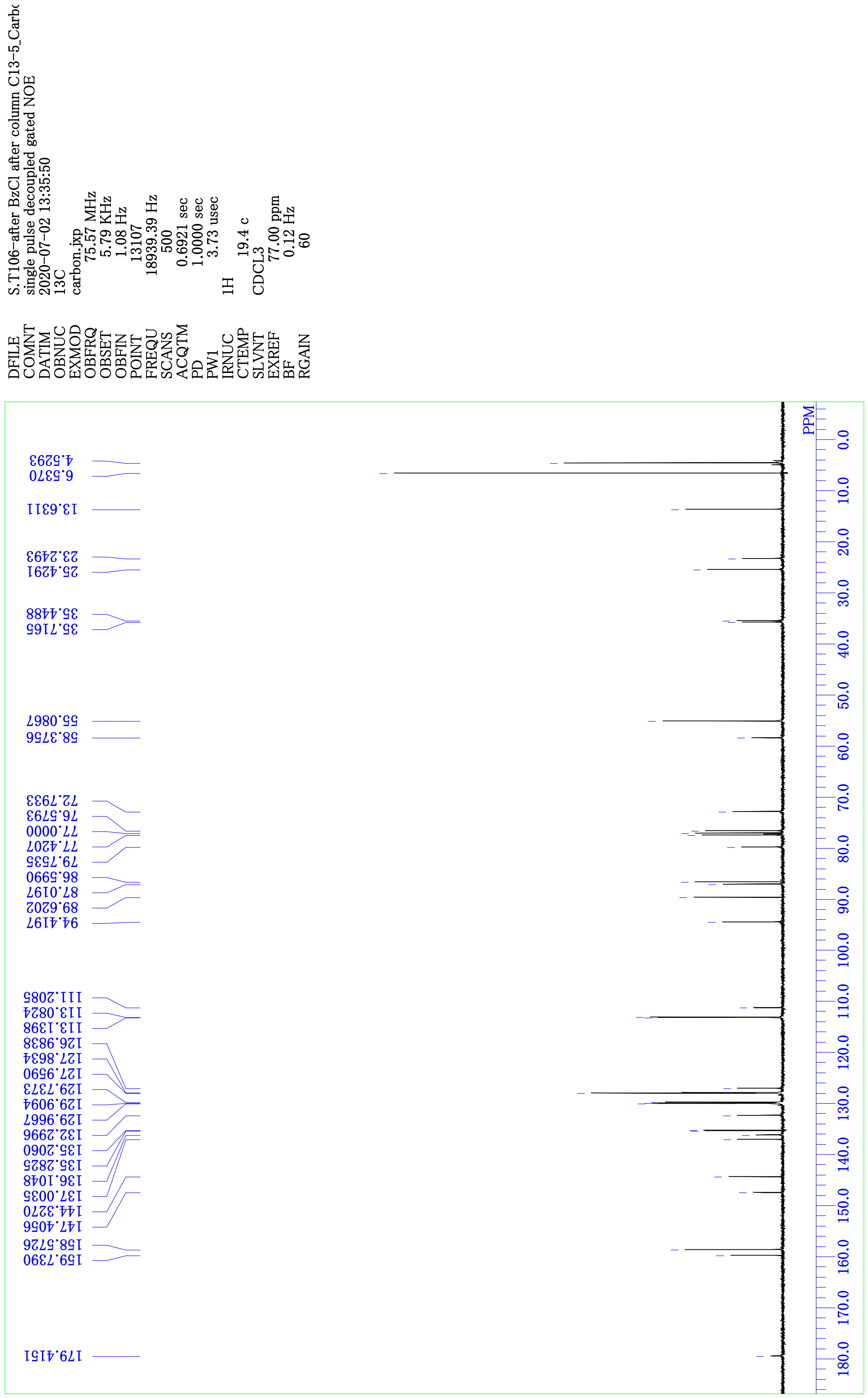

Compound **16** (^{1}H-NMR, $CDCl_3$, 300 MHz)

DFILE	S.T107–after datuhogo after column3_proton–
COMNT	single_pulse
DATIM	2020–07–07 11:49:50
OBNUC	1H
EXMOD	proton.jxp
OBFRQ	300.53 MHz
OBSET	1.15 KHz
OBFIN	8.57 Hz
POINT	13107
FREQU	4512.64 Hz
SCANS	32
ACQTM	2.9046 sec
PD	2.0000 sec
PW1	5.50 usec
IRNUC	1H
CTEMP	21.2 c
SLVNT	CDCL3
EXREF	7.26 ppm
BF	0.12 Hz
RGAIN	38

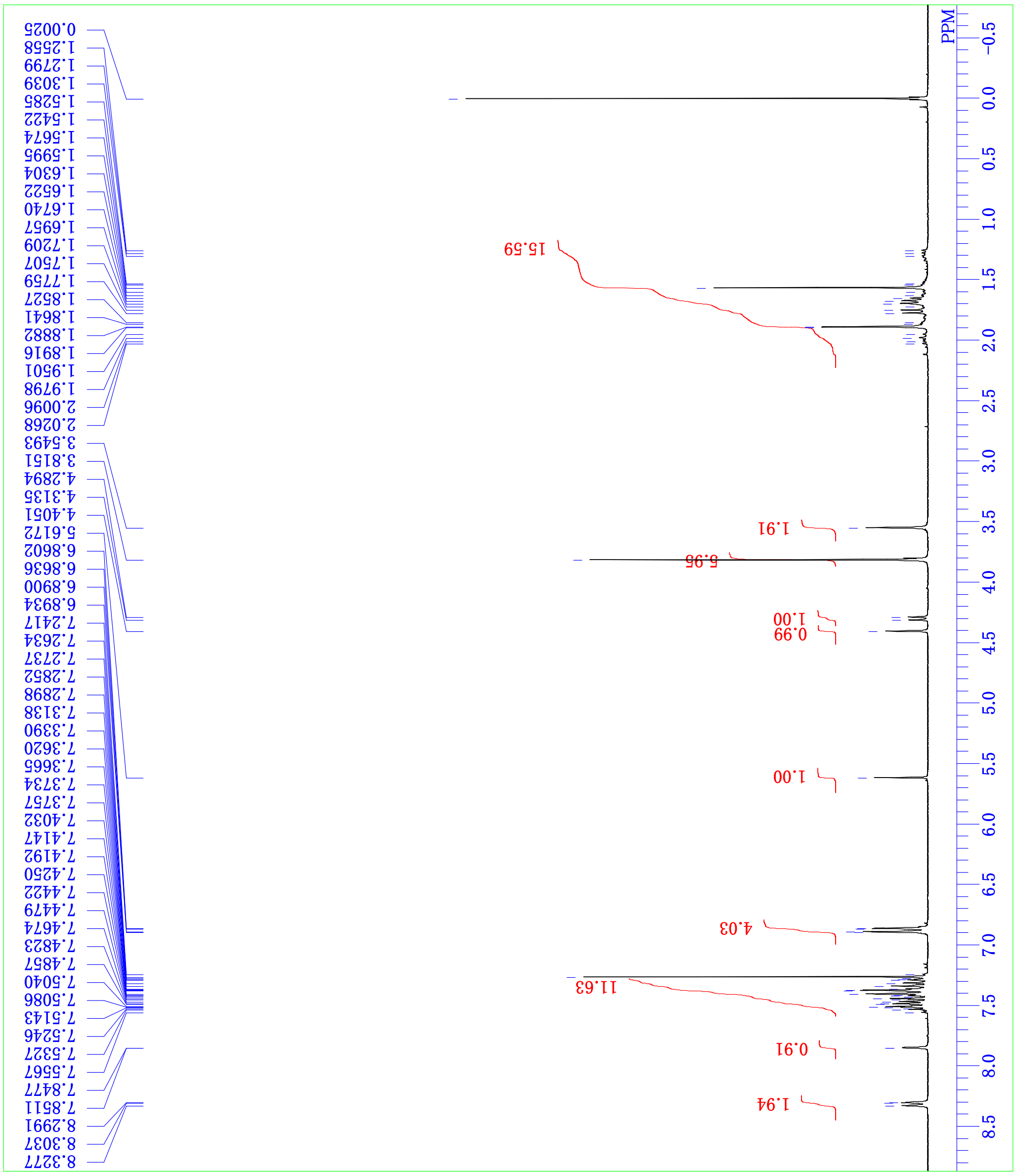

Compound **16** (^{13}C NMR, $CDCl_3$, 75.6 MHz)

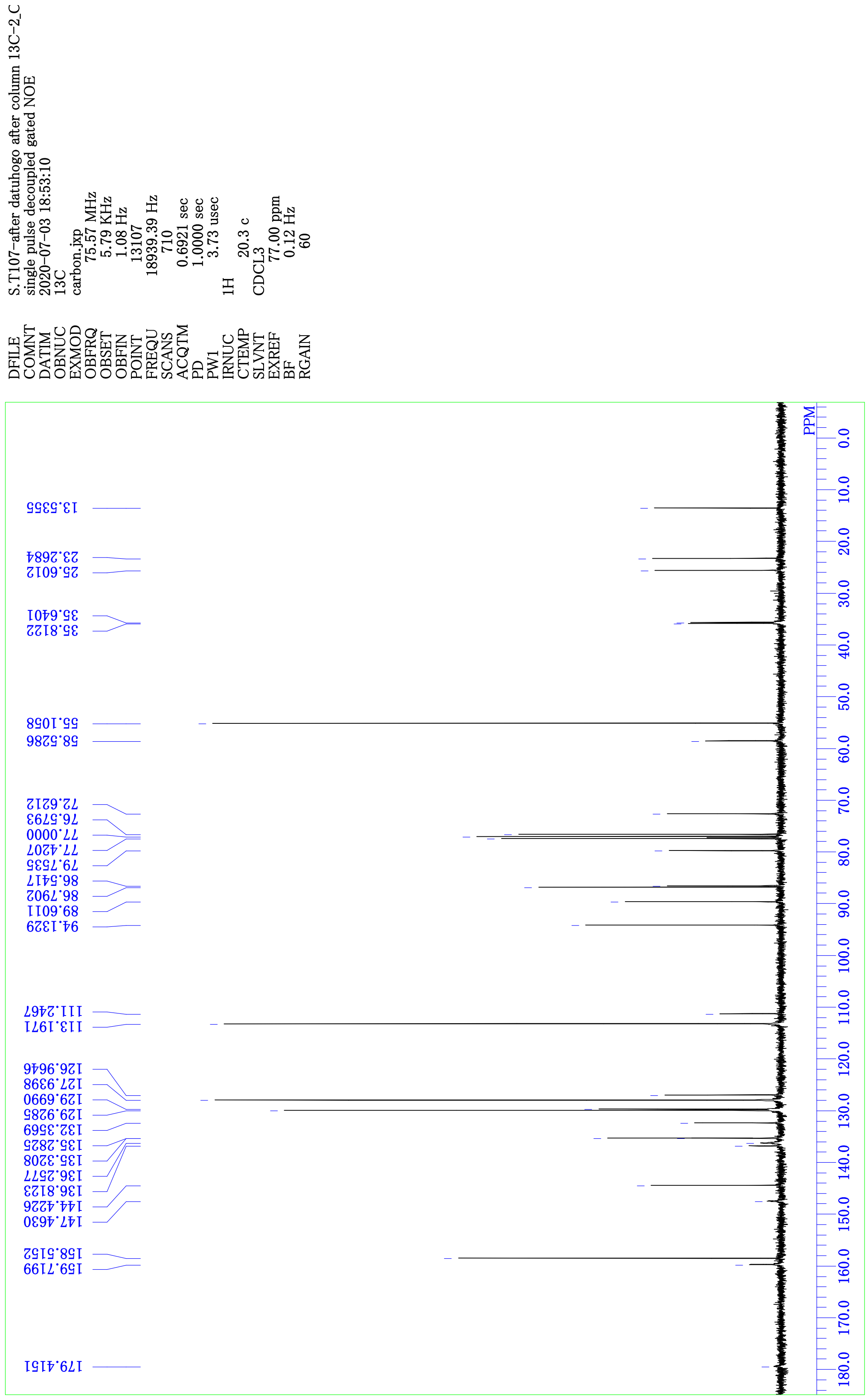

Compound **17** (^{1}H-NMR, $CDCl_3$, 500 MHz)

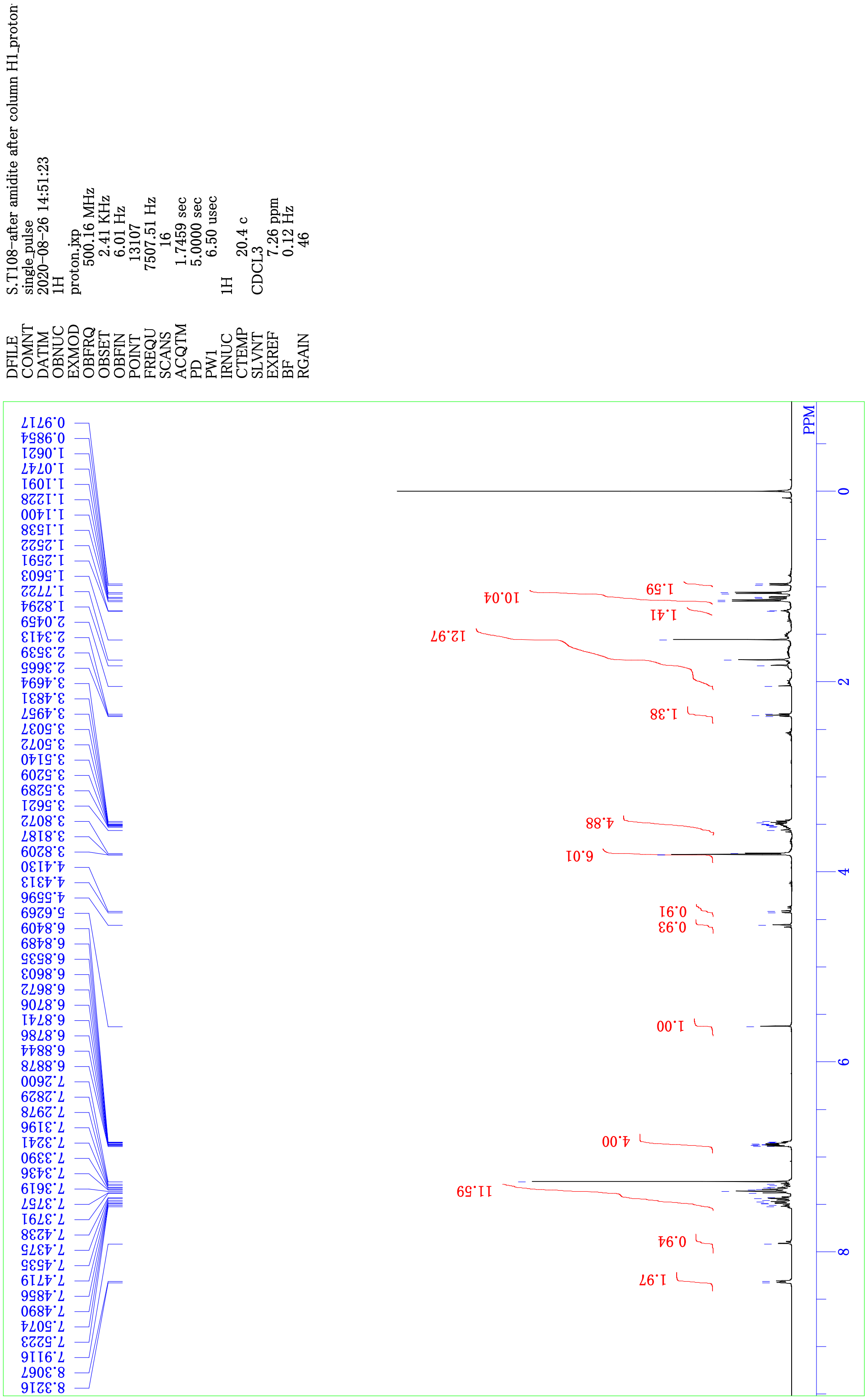

Compound **17** (^{31}P-NMR, $CDCl_3$, 161.8 MHz)

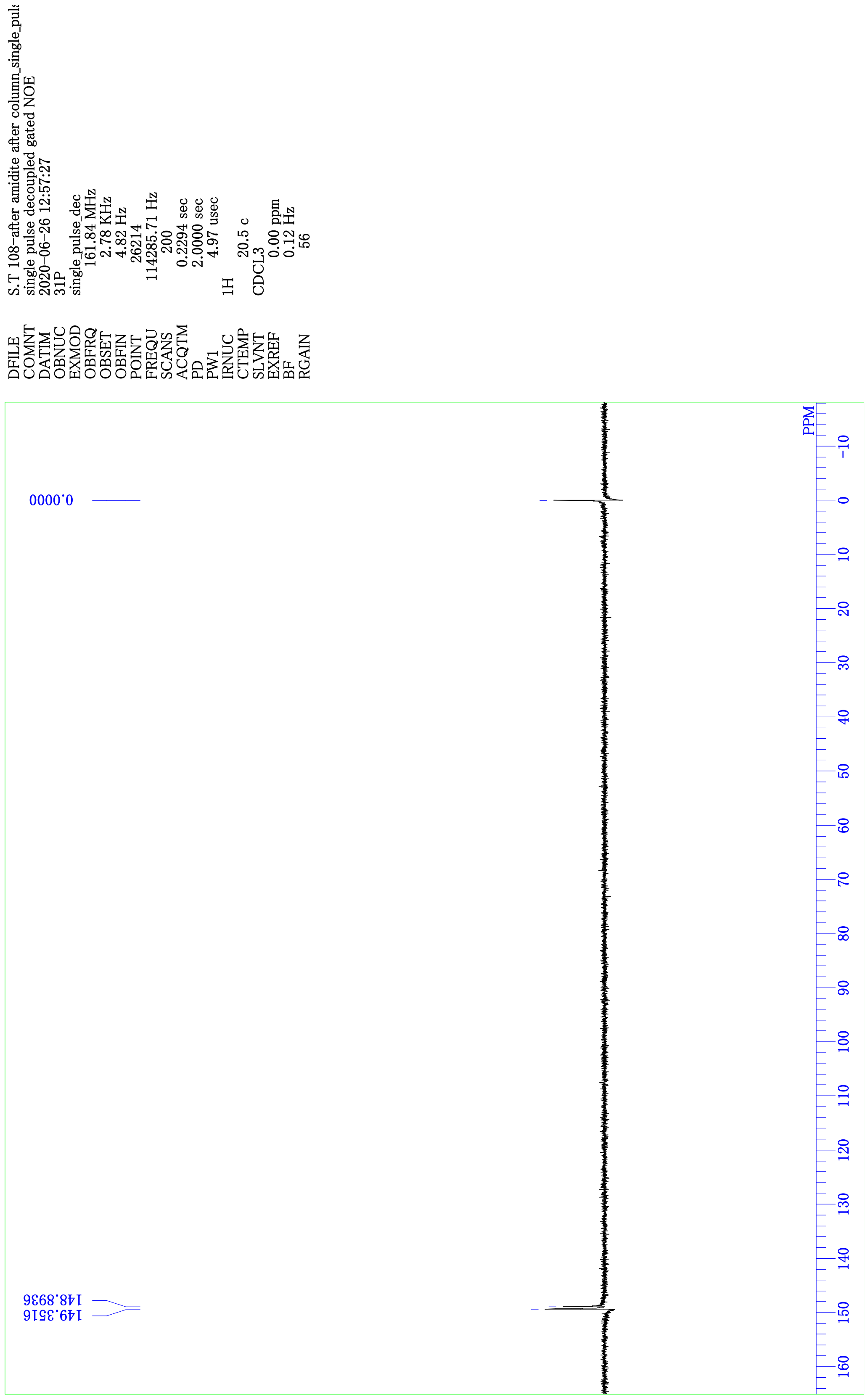

4. Characterization data (HPLC and mass data) of synthesized oligonucleotide

HPLC (**ON1**)

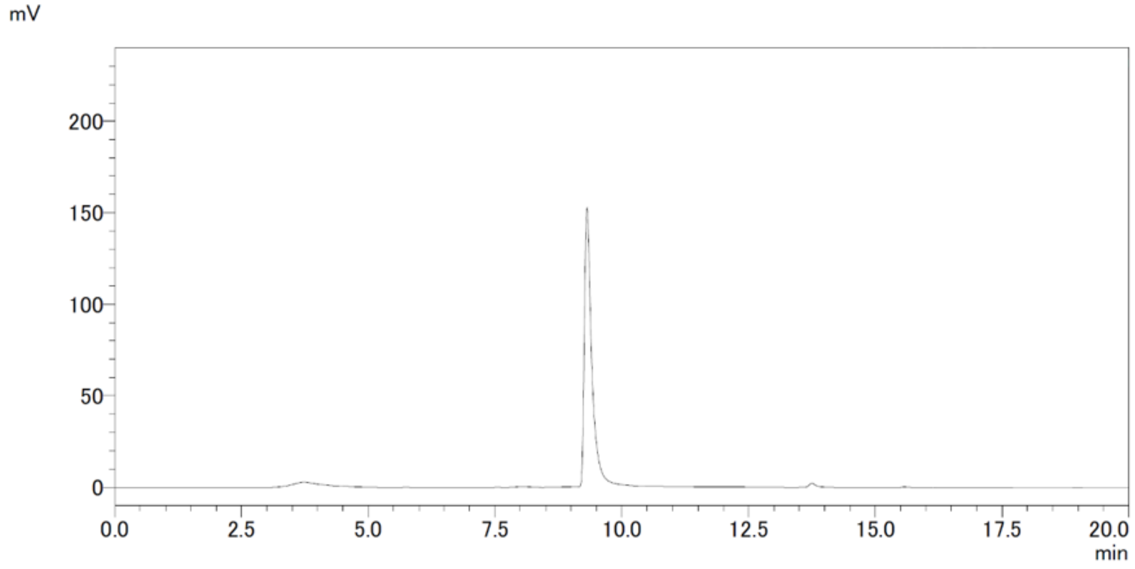

MALDI-TOF MS (**ON1**)

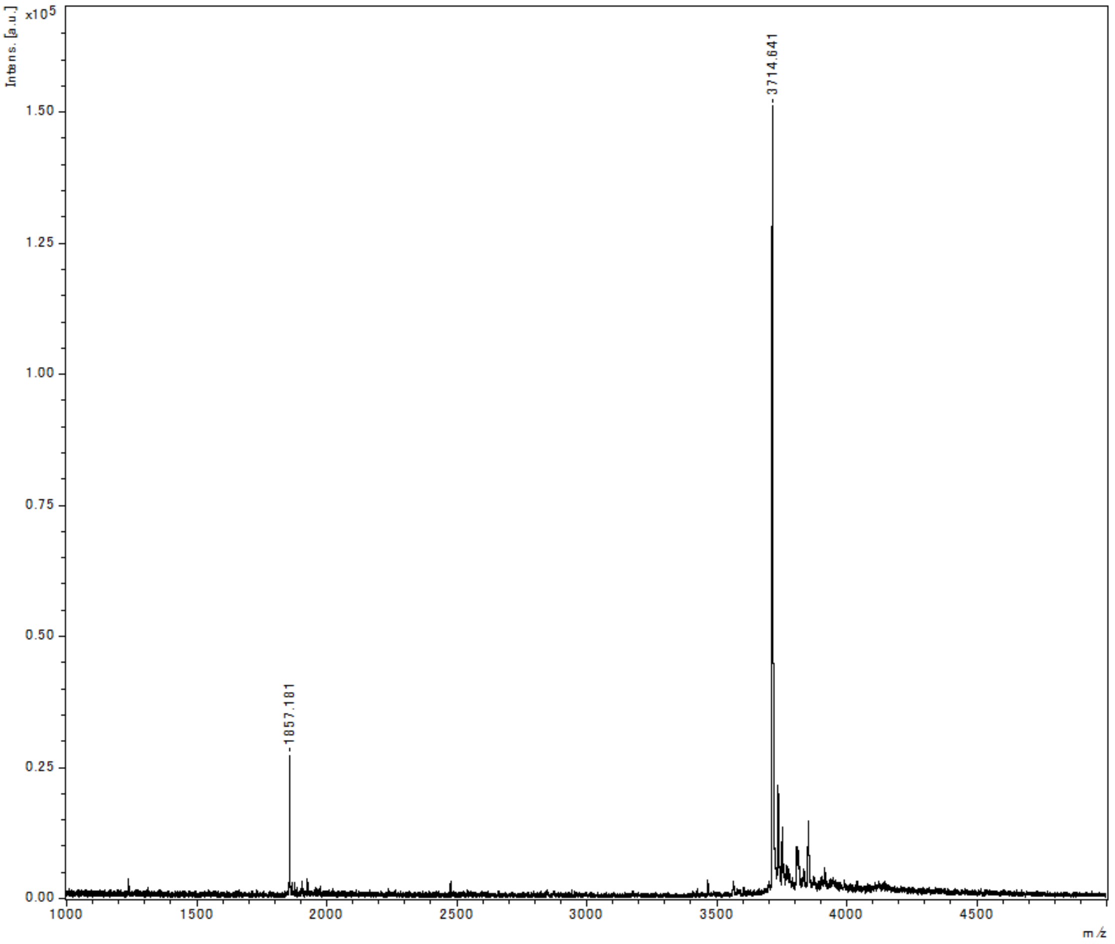

HPLC (**ON2**)

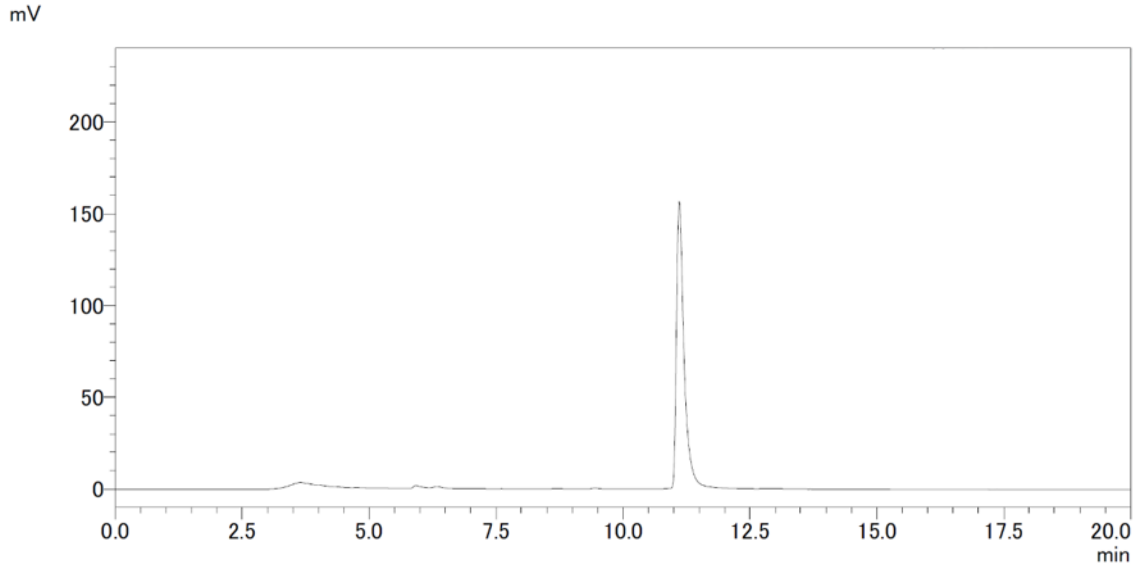

MALDI-TOF MS (**ON2**)

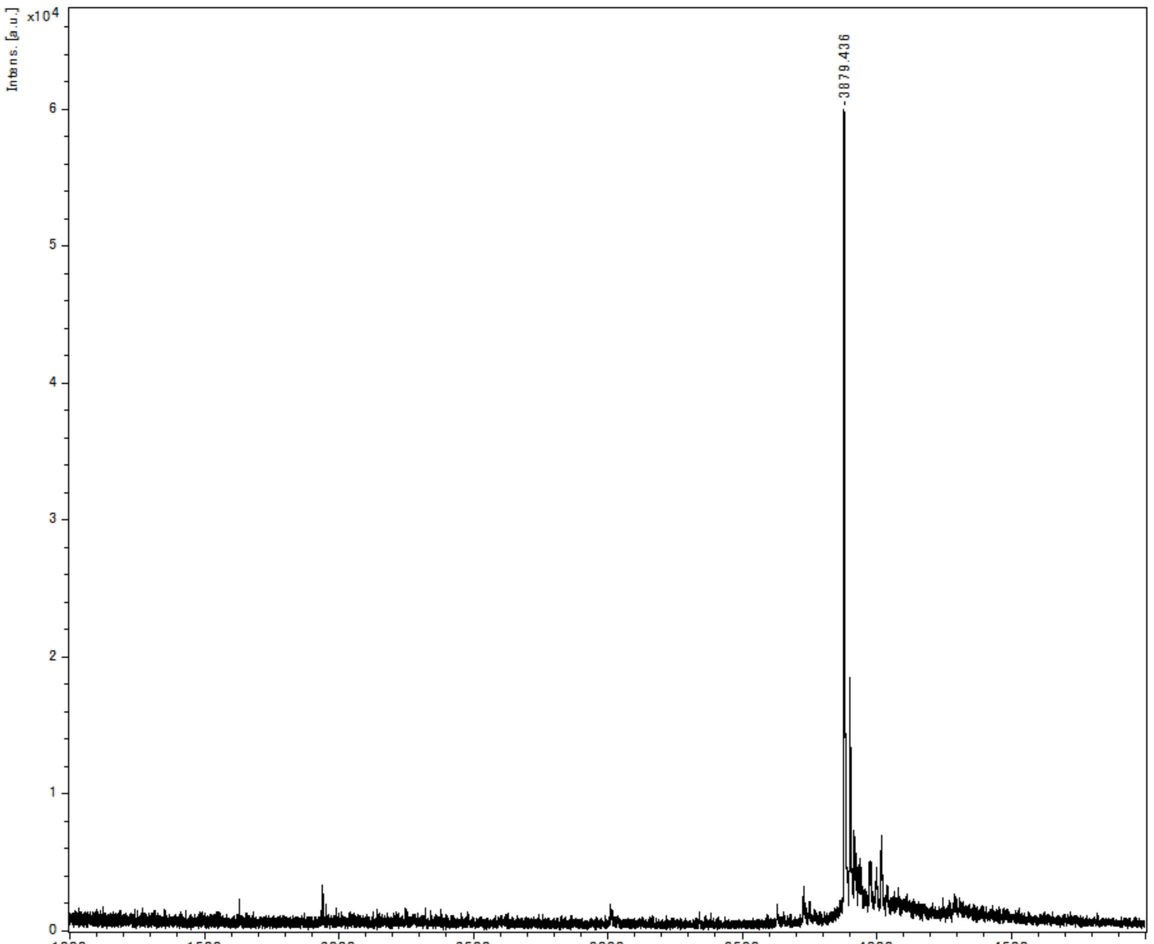

HPLC (**ON3**)

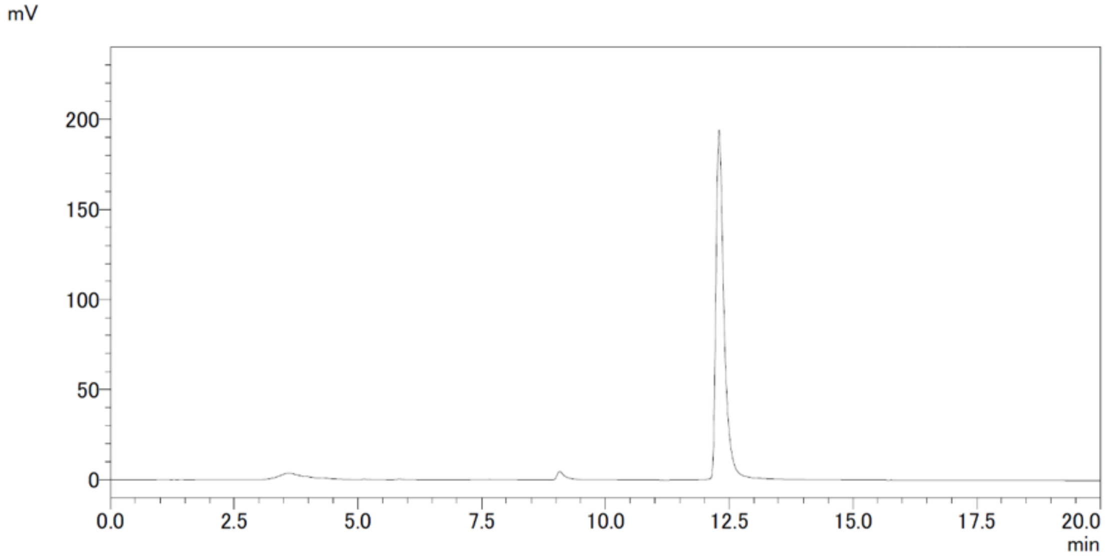

MALDI-TOF MS (**ON3**)

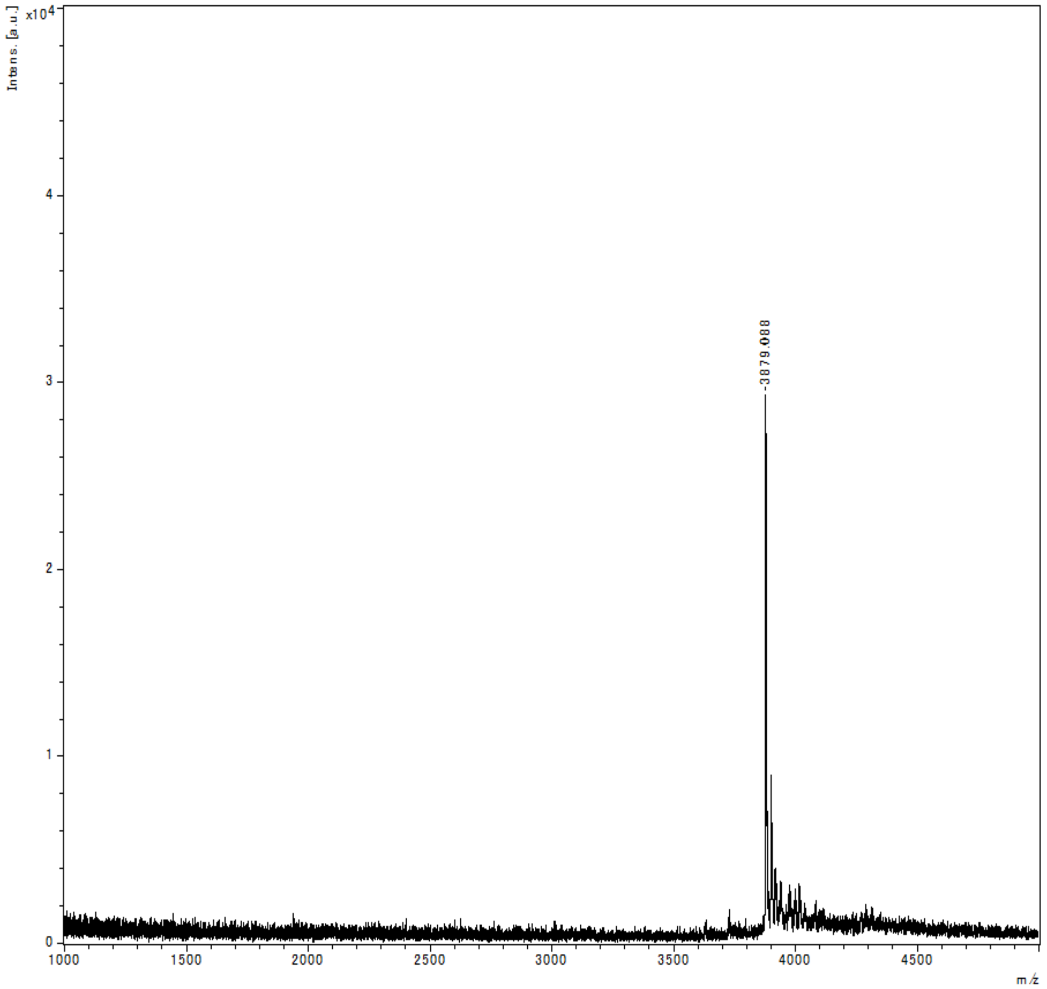

HPLC (**ON4**)

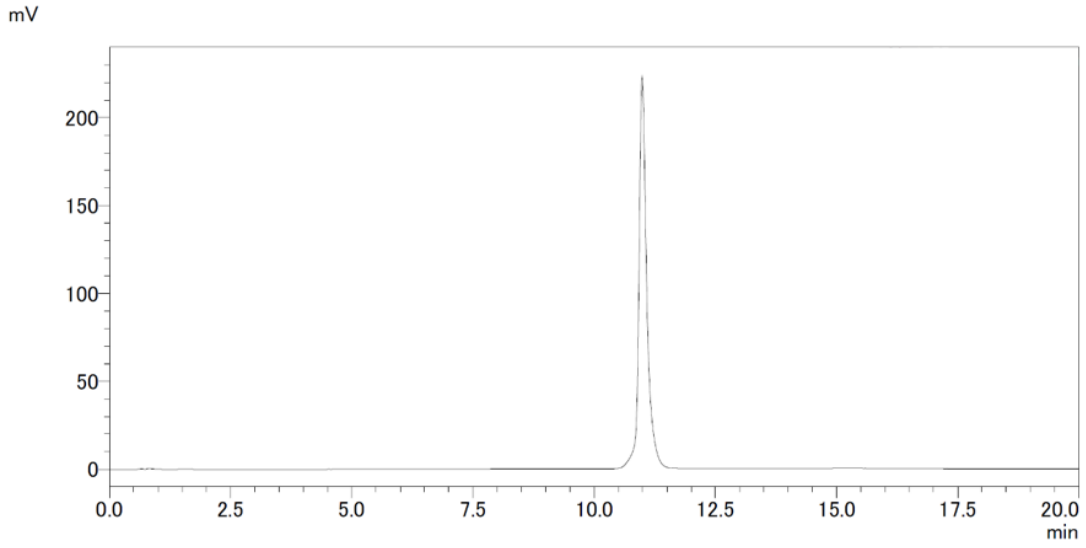

MALDI-TOF MS (**ON4**)

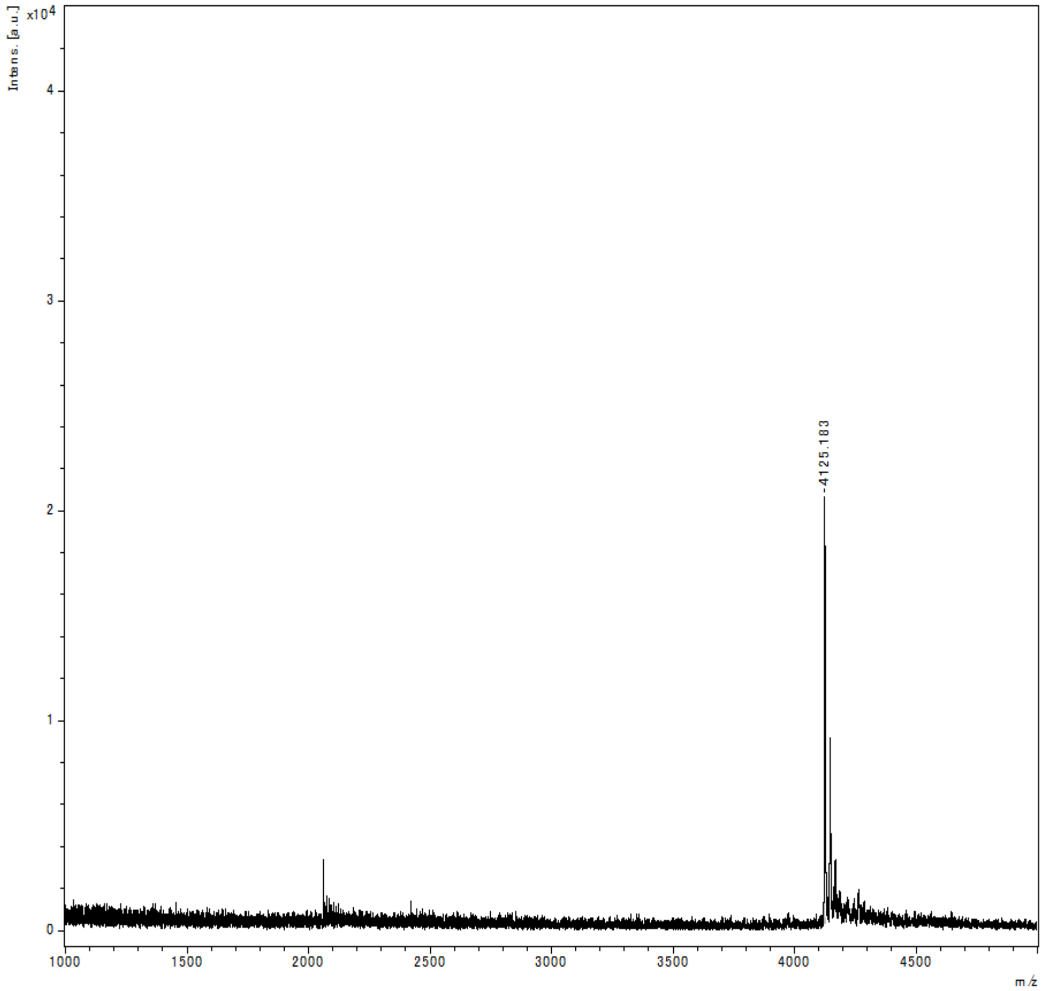

HPLC (**ON5**)

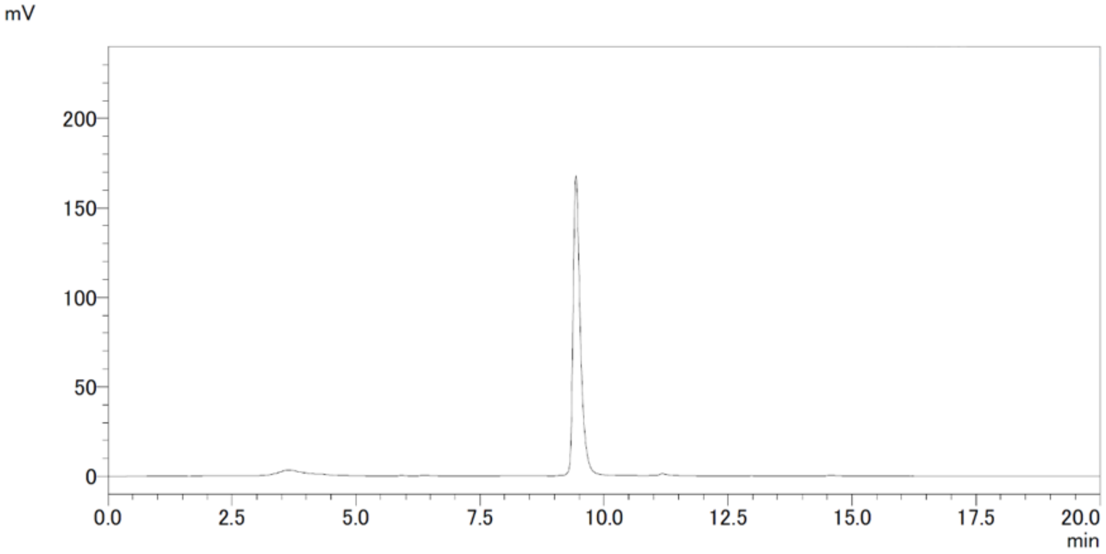

MALDI-TOF MS (**ON5**)

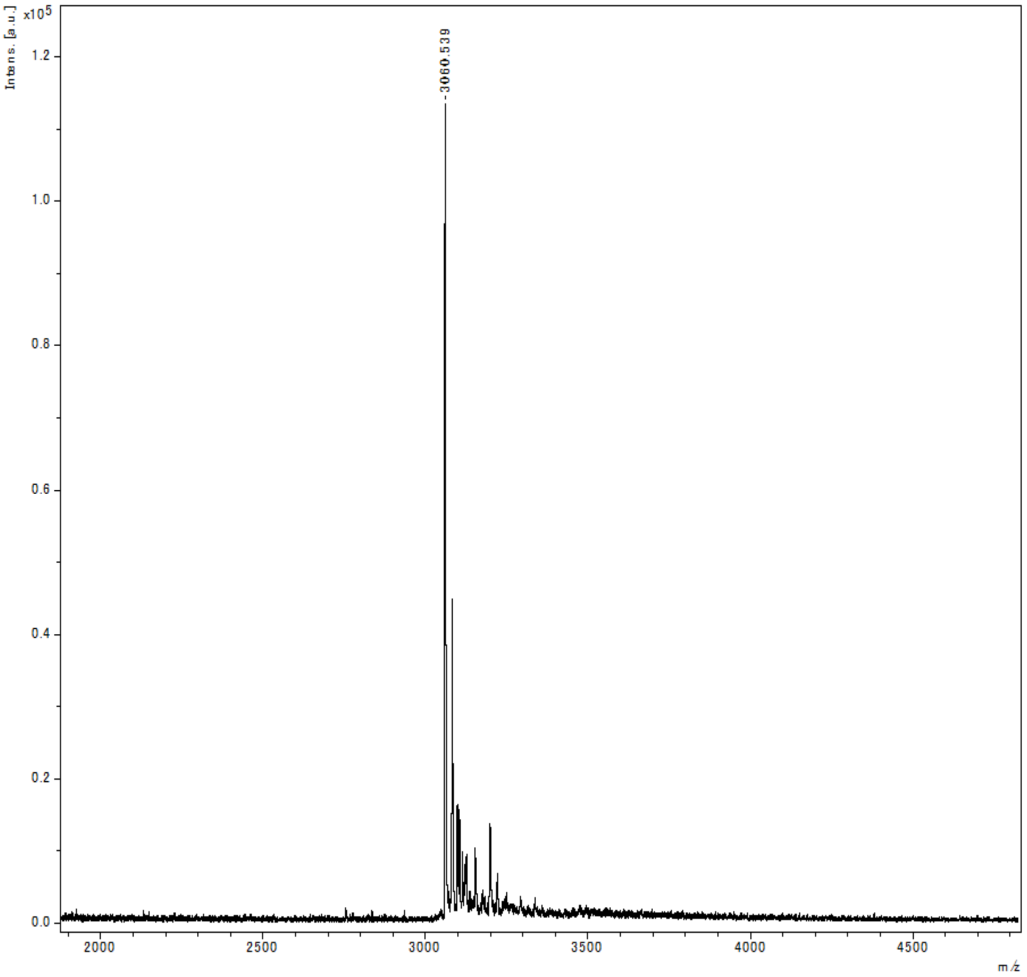

HPLC (**ON14**)

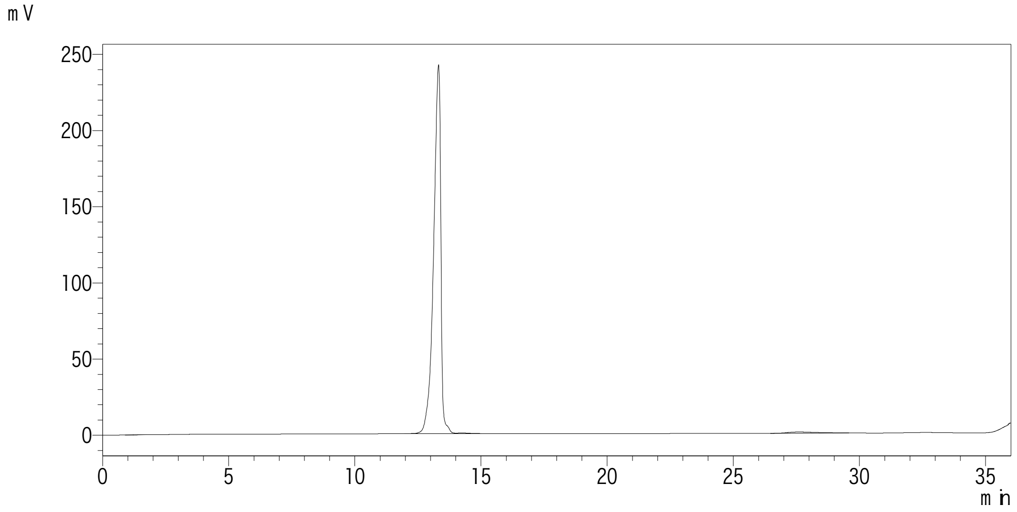

MALDI-TOF MS (**ON14**)

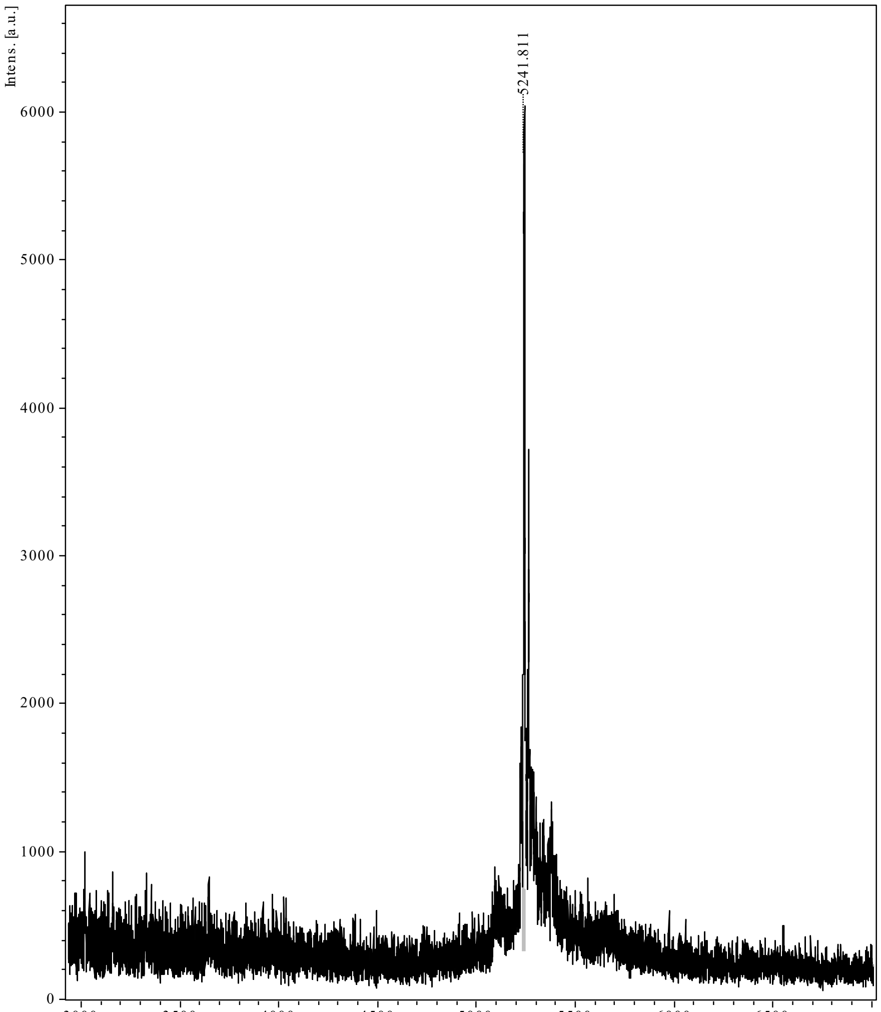

HPLC (**ON15**)

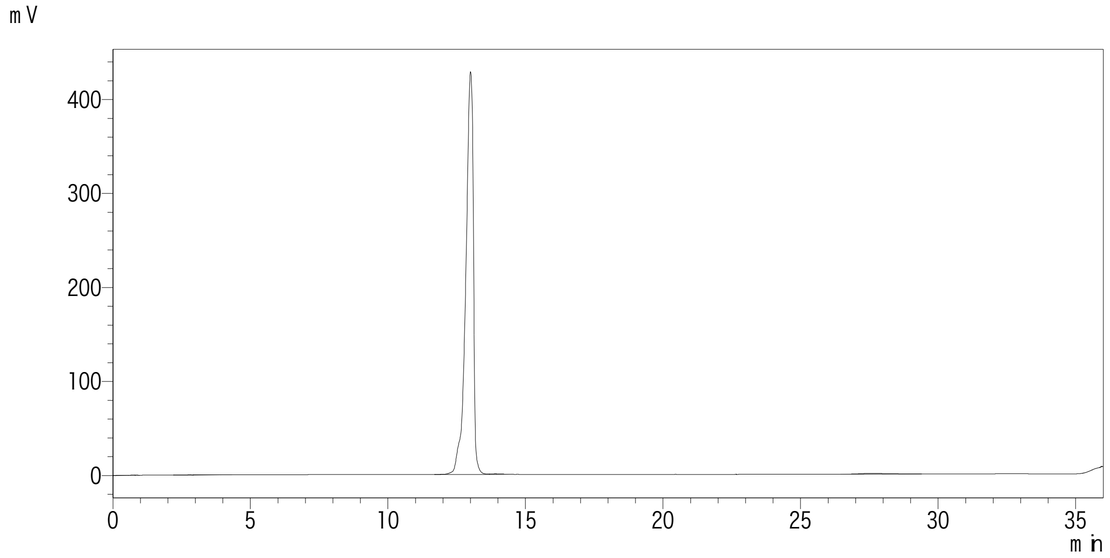

MALDI-TOF MS (**ON15**)

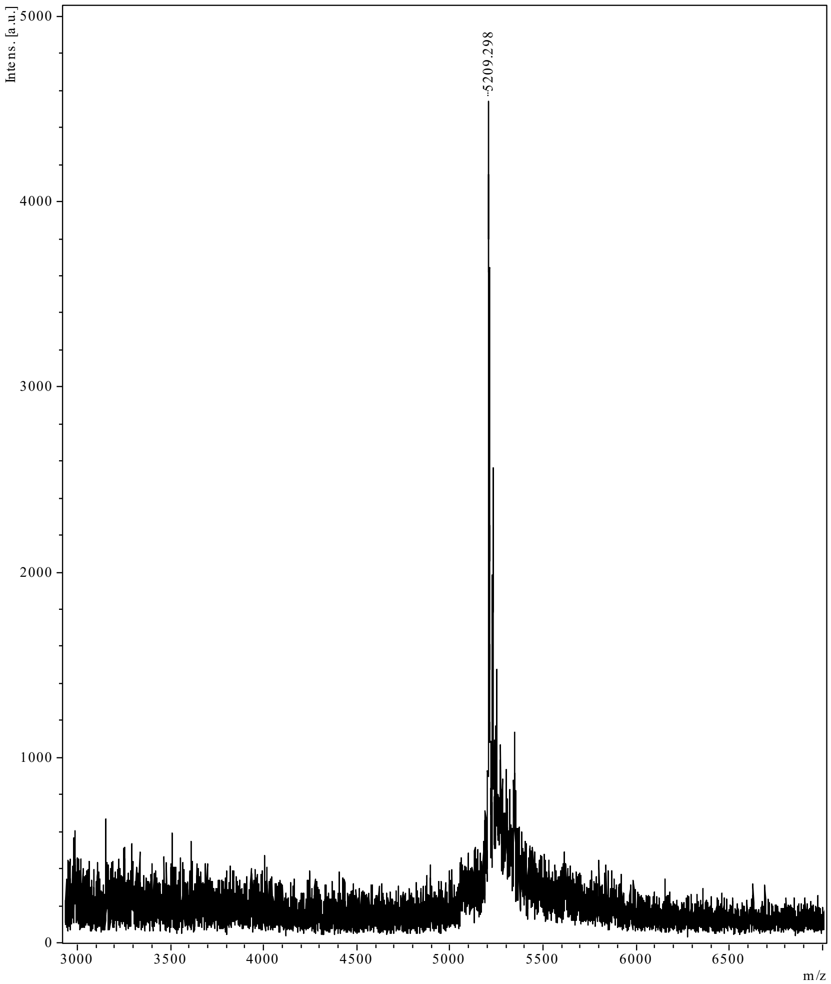

HPLC (**ON16**)

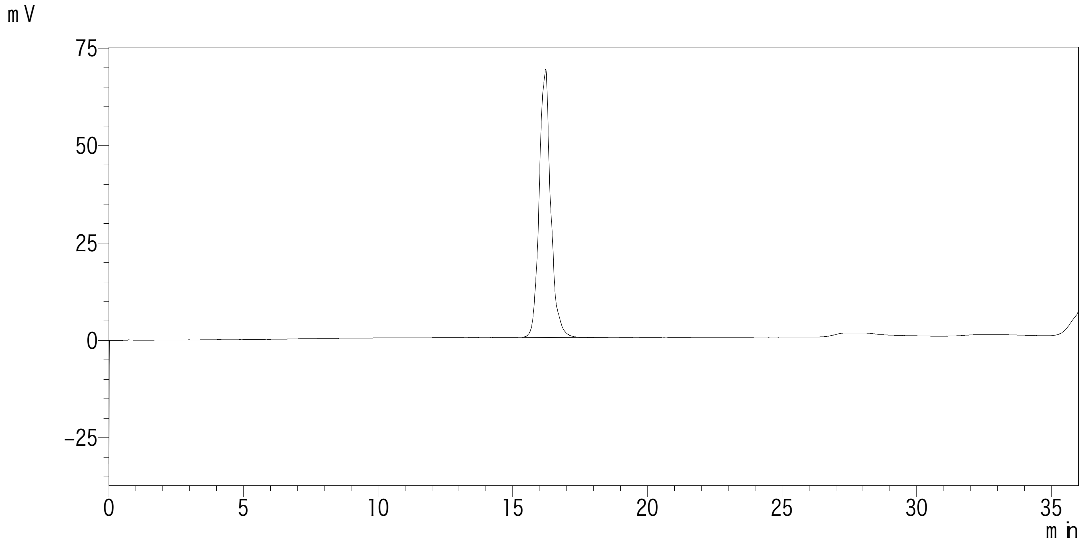

MALDI-TOF MS (**ON16**)

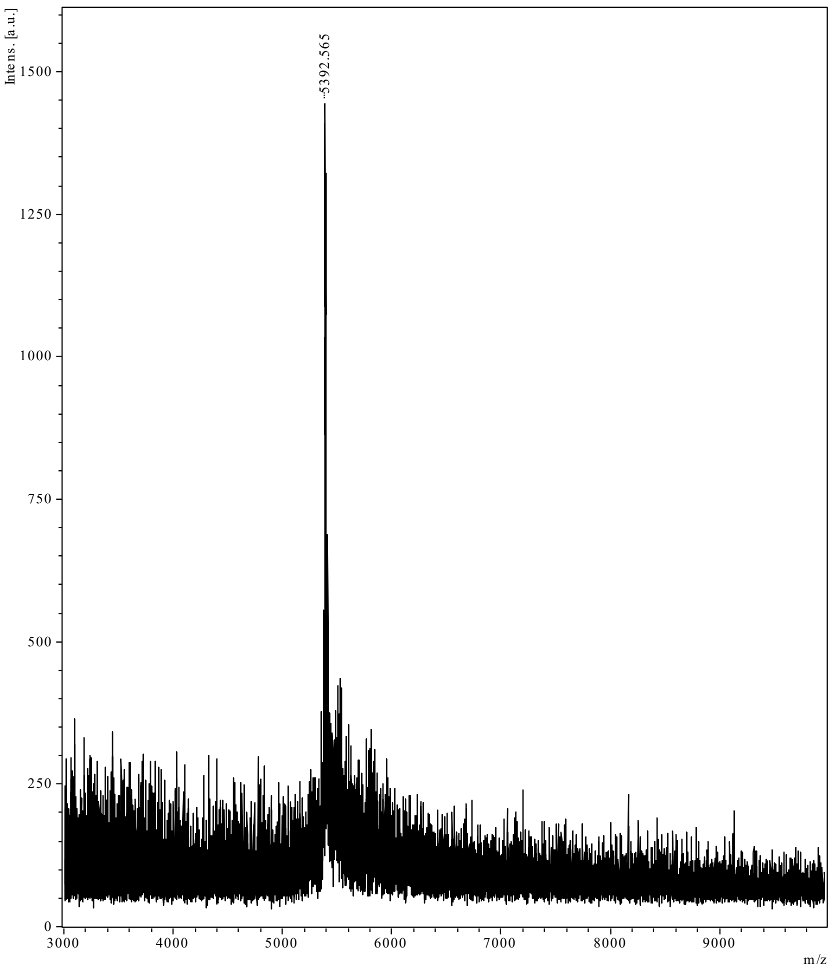

HPLC (**ON17**)

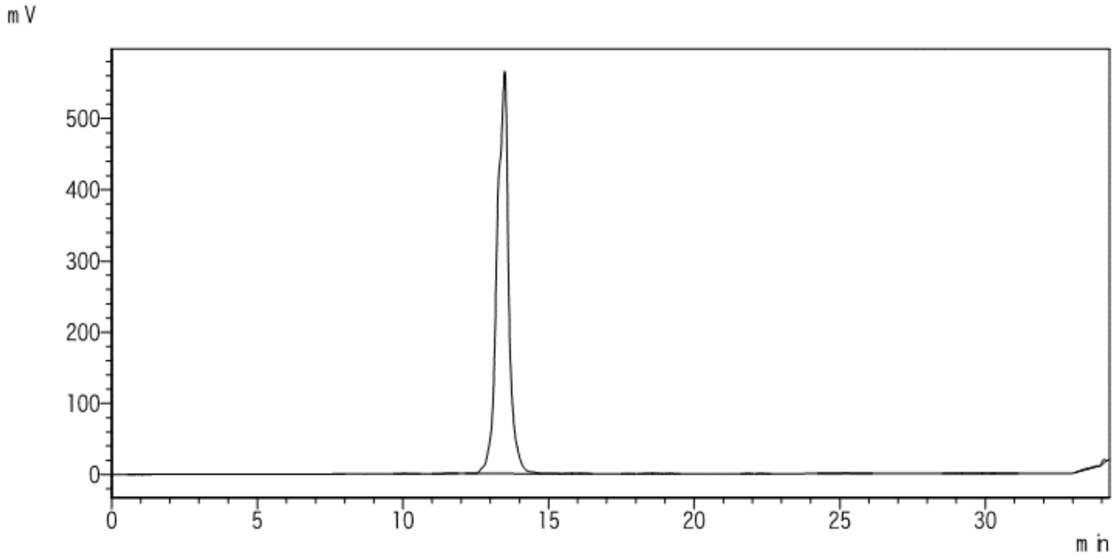

MALDI-TOF MS (**ON17**)

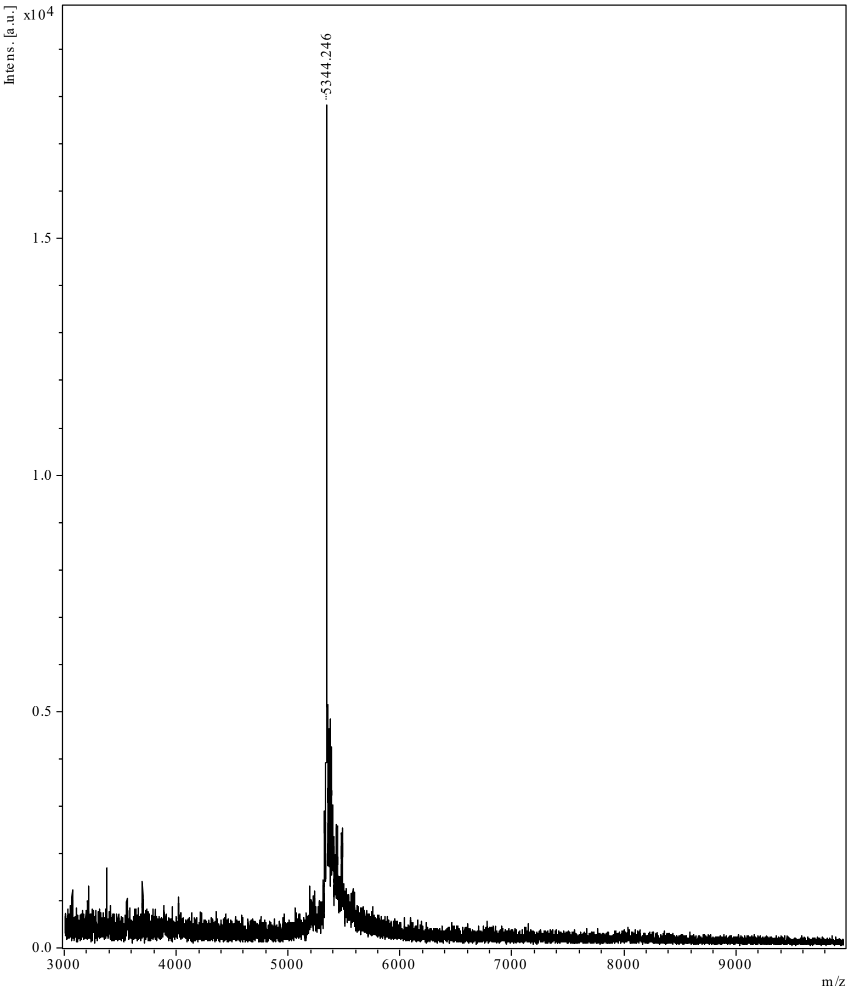

HPLC (**ON18**)

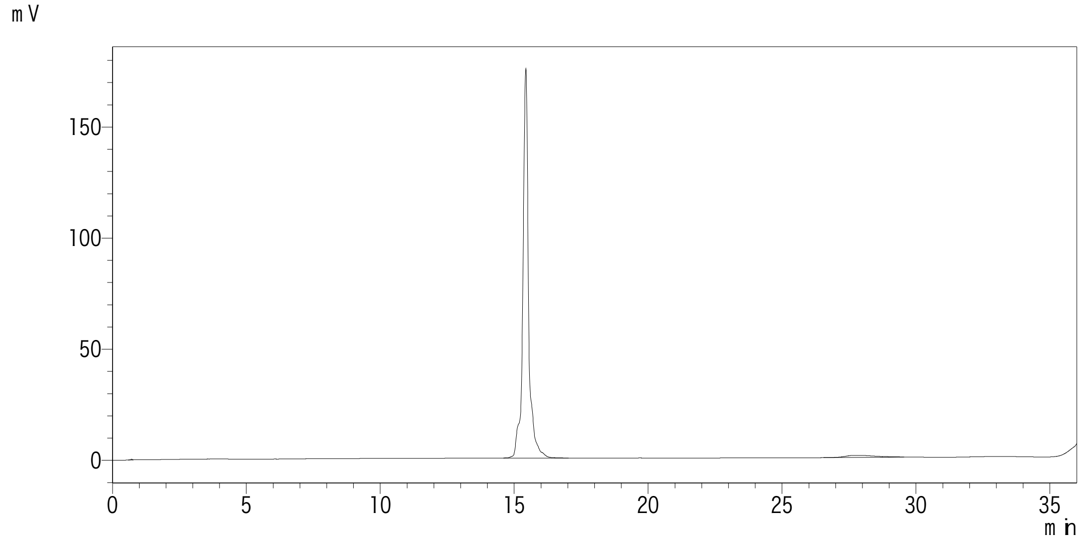

MALDI-TOF MS (**ON18**)

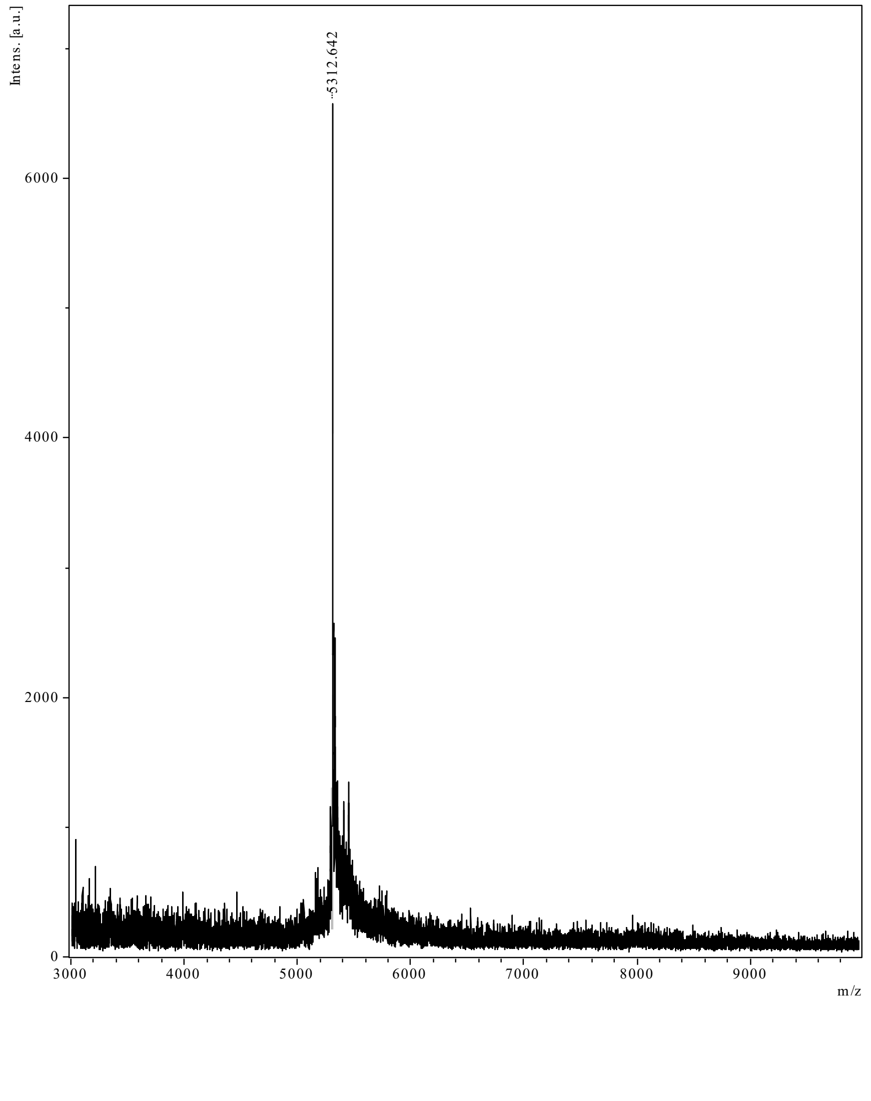

HPLC (**ON19**)

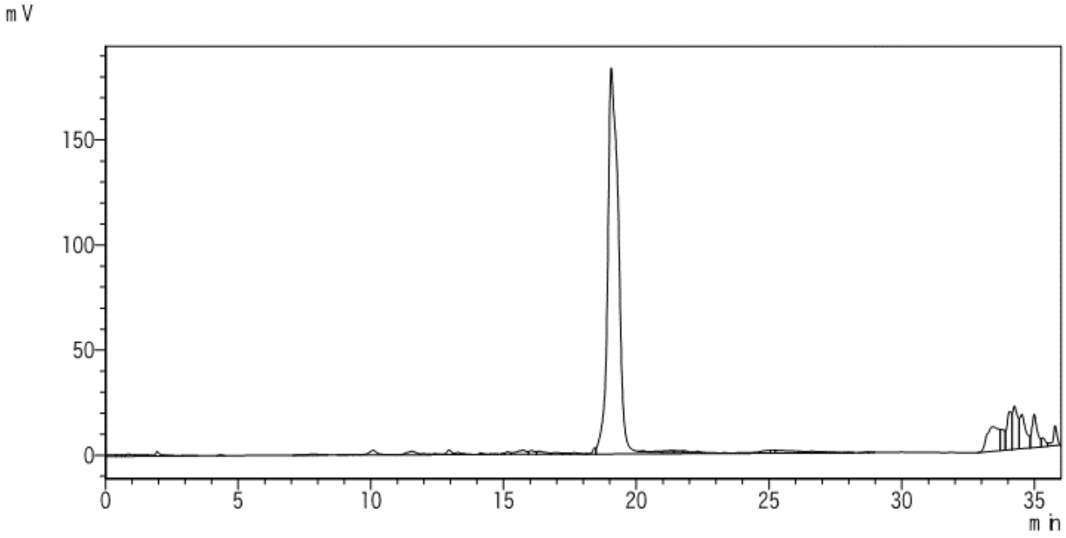

MALDI-TOF MS (**ON19**)

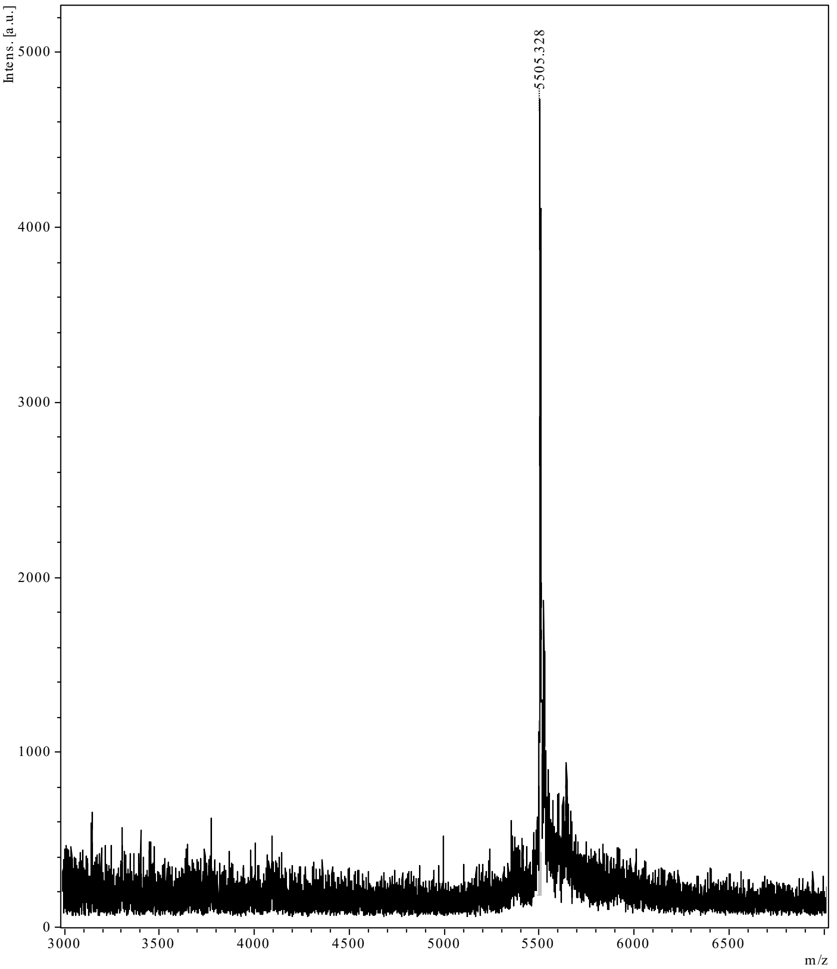

HPLC (**ON20**)

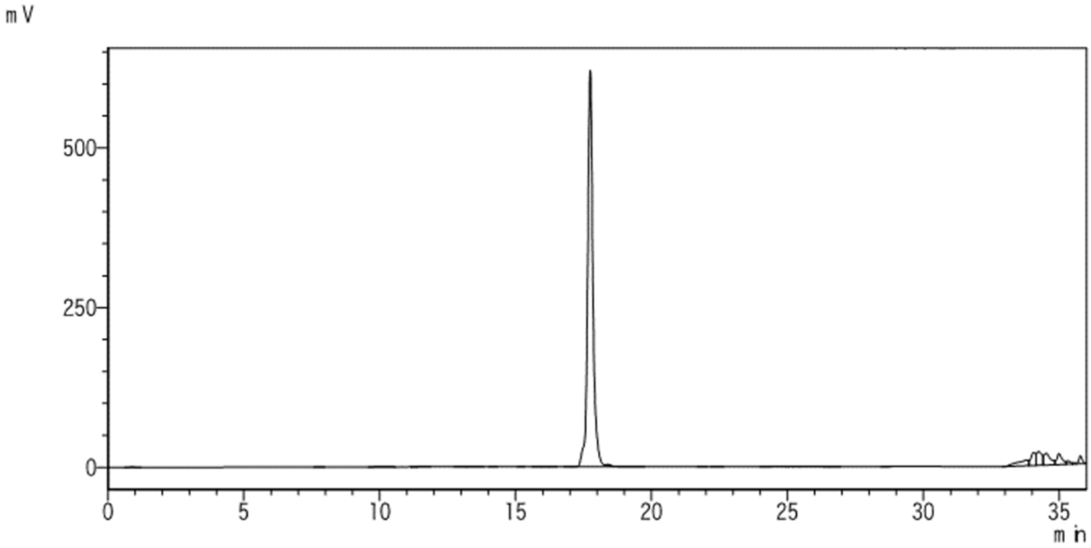

MALDI-TOF MS (**ON20**)

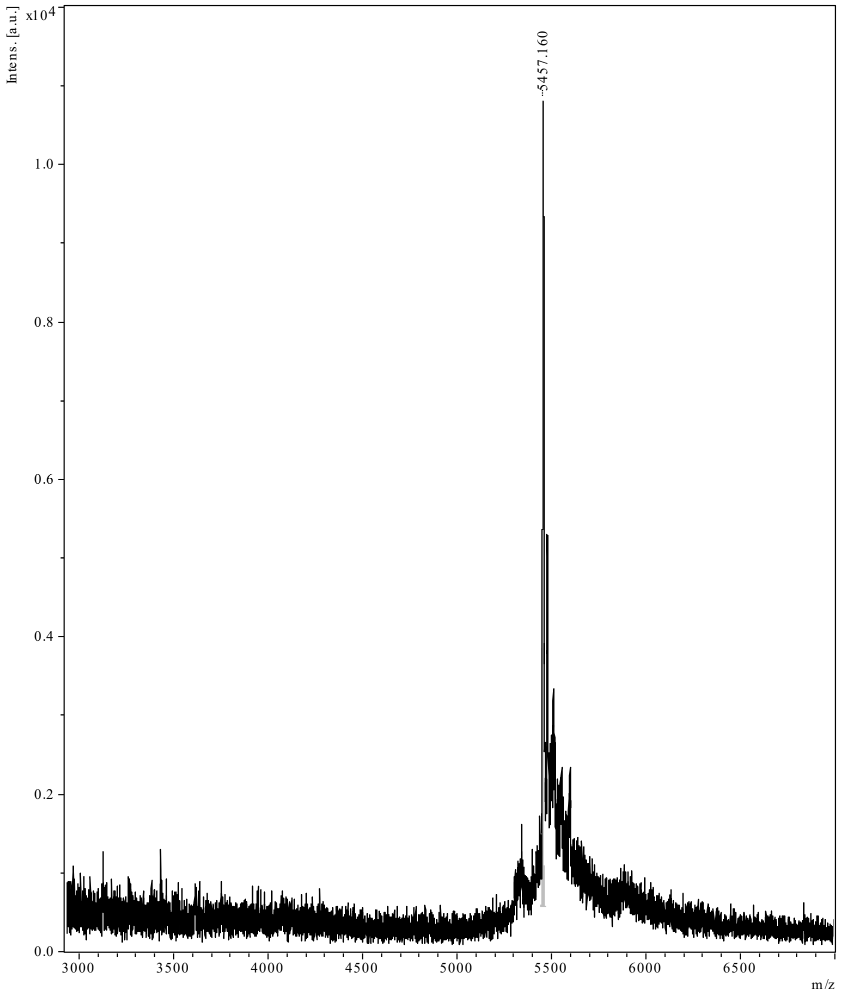

HPLC (**ON21**)

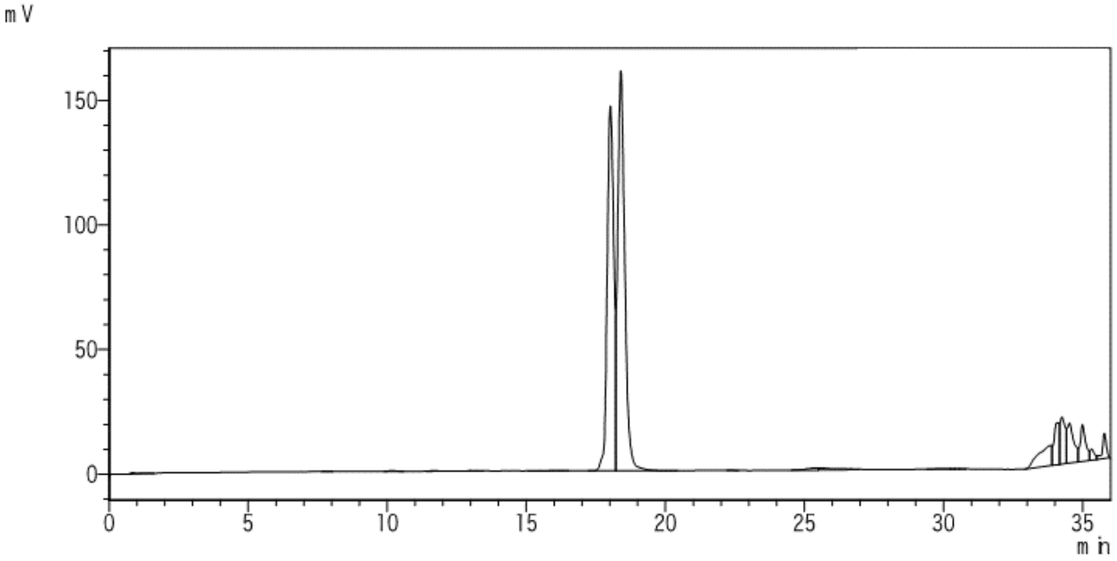

MALDI-TOF MS (**ON21**)

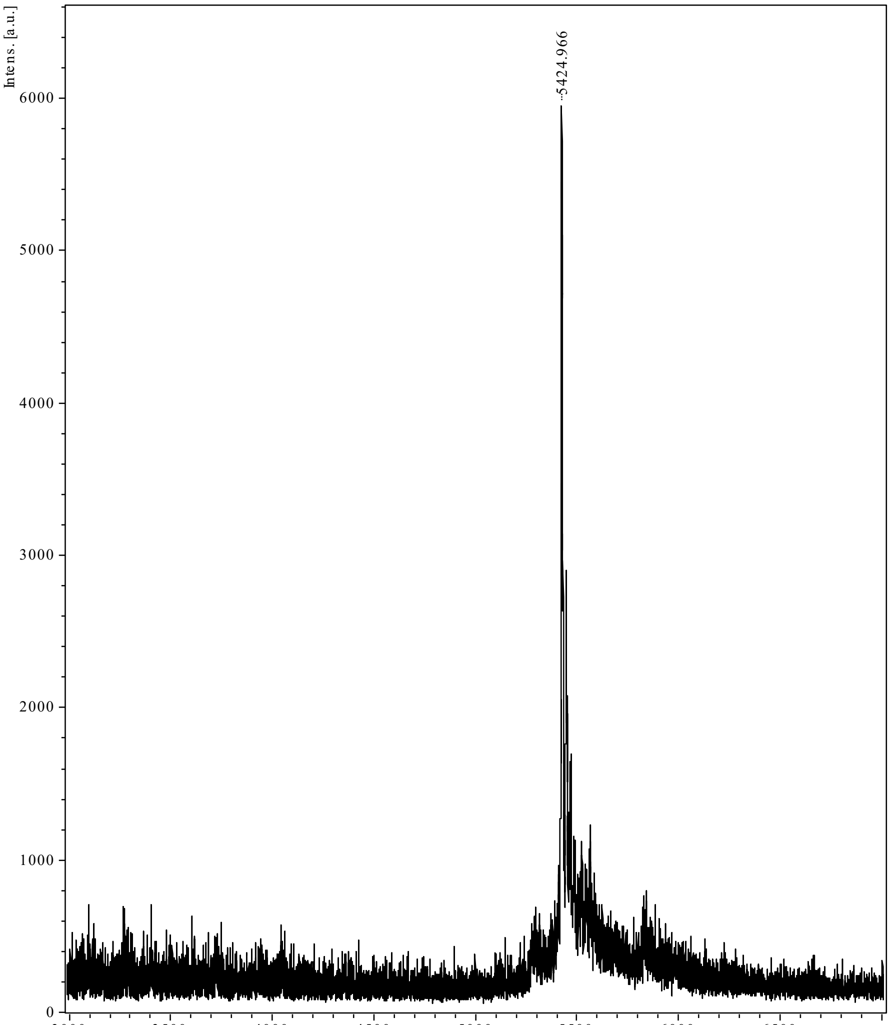

HPLC (**ON24**)

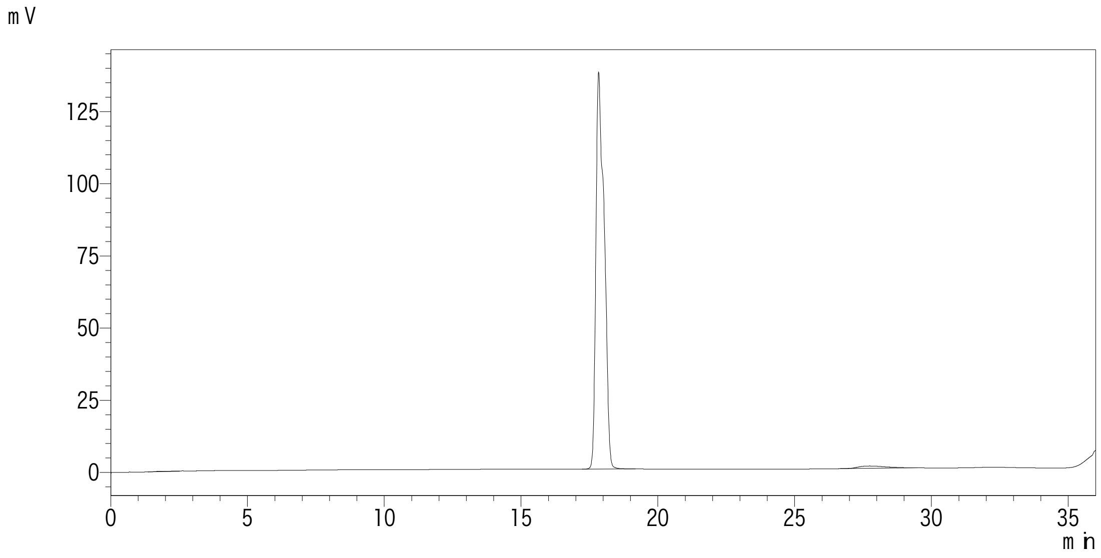

MALDI-TOF MS (**ON24**)

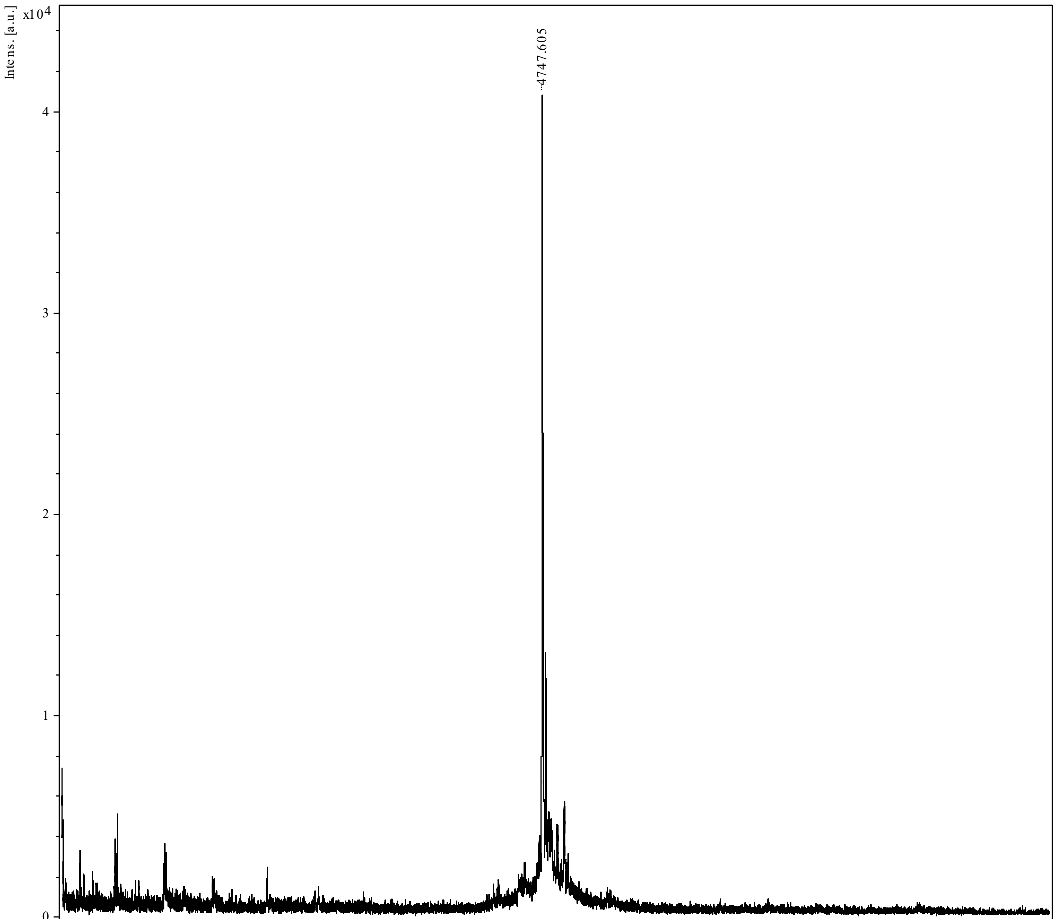

HPLC (**ON25**)

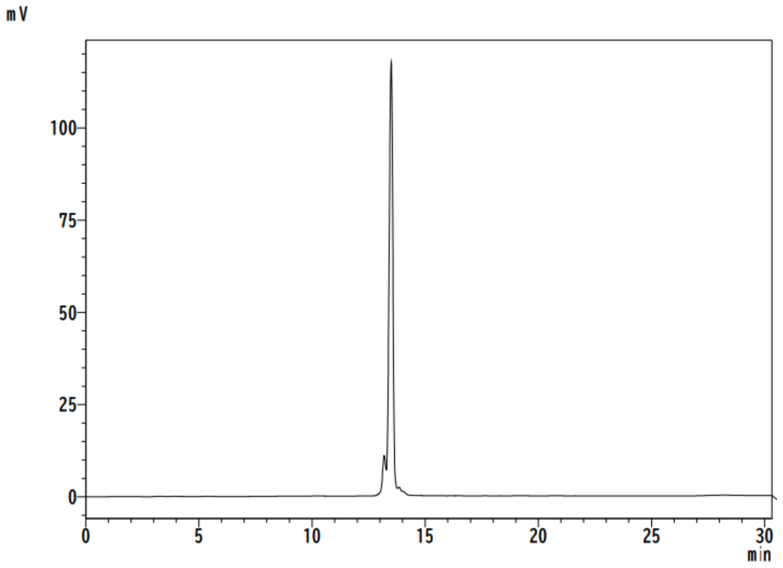

MALDI-TOF MS (**ON25**)

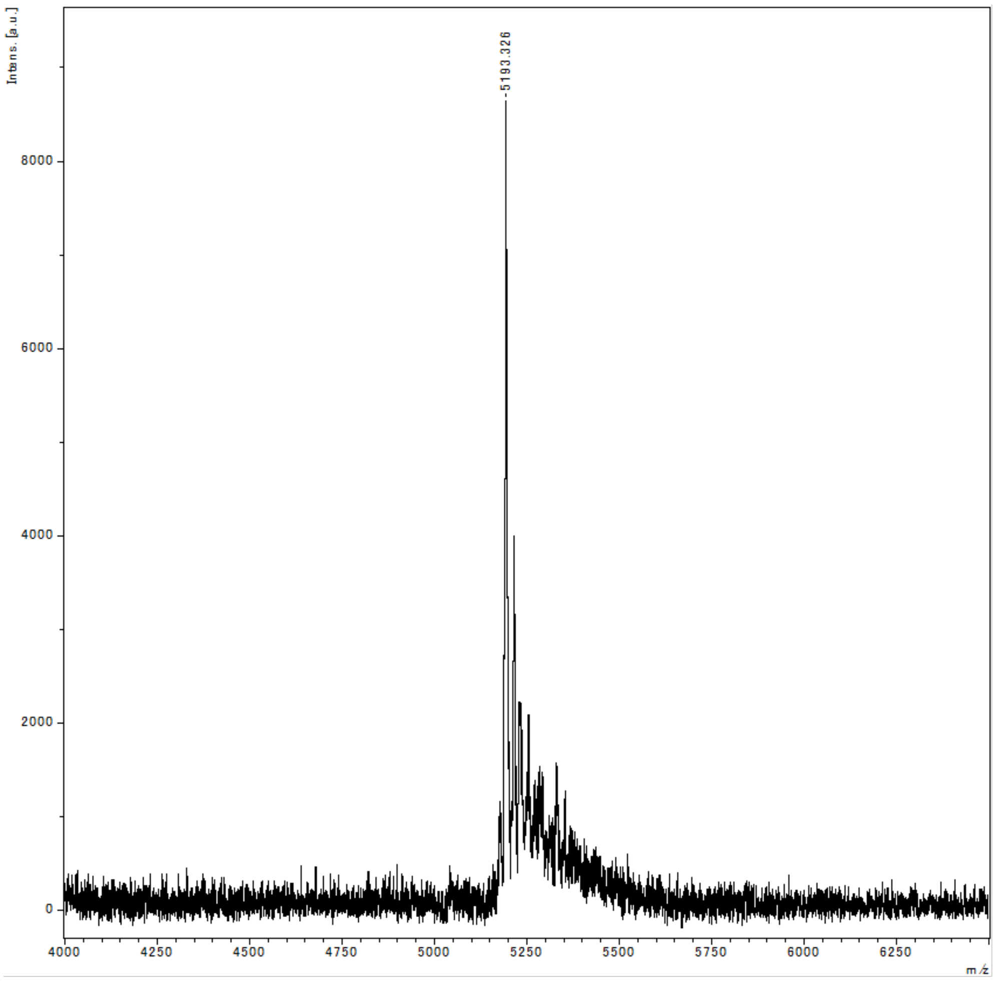

HPLC (**ON26**)

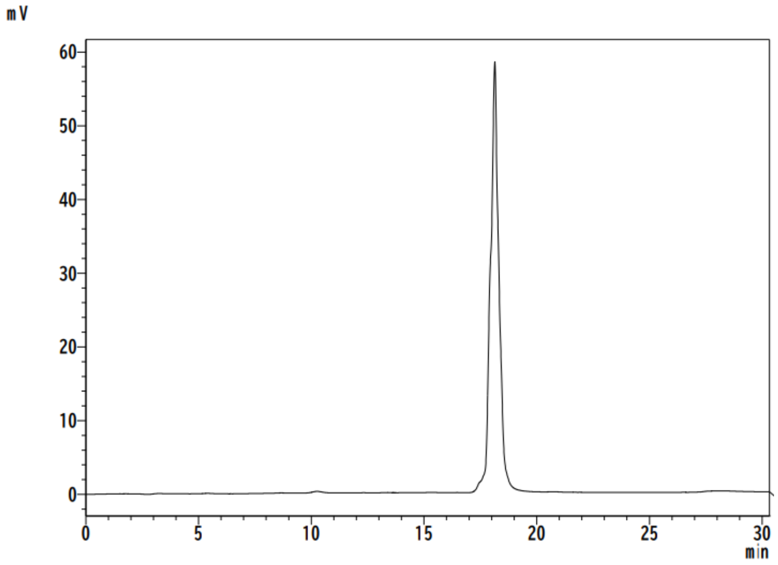

MALDI-TOF MS (**ON26**)

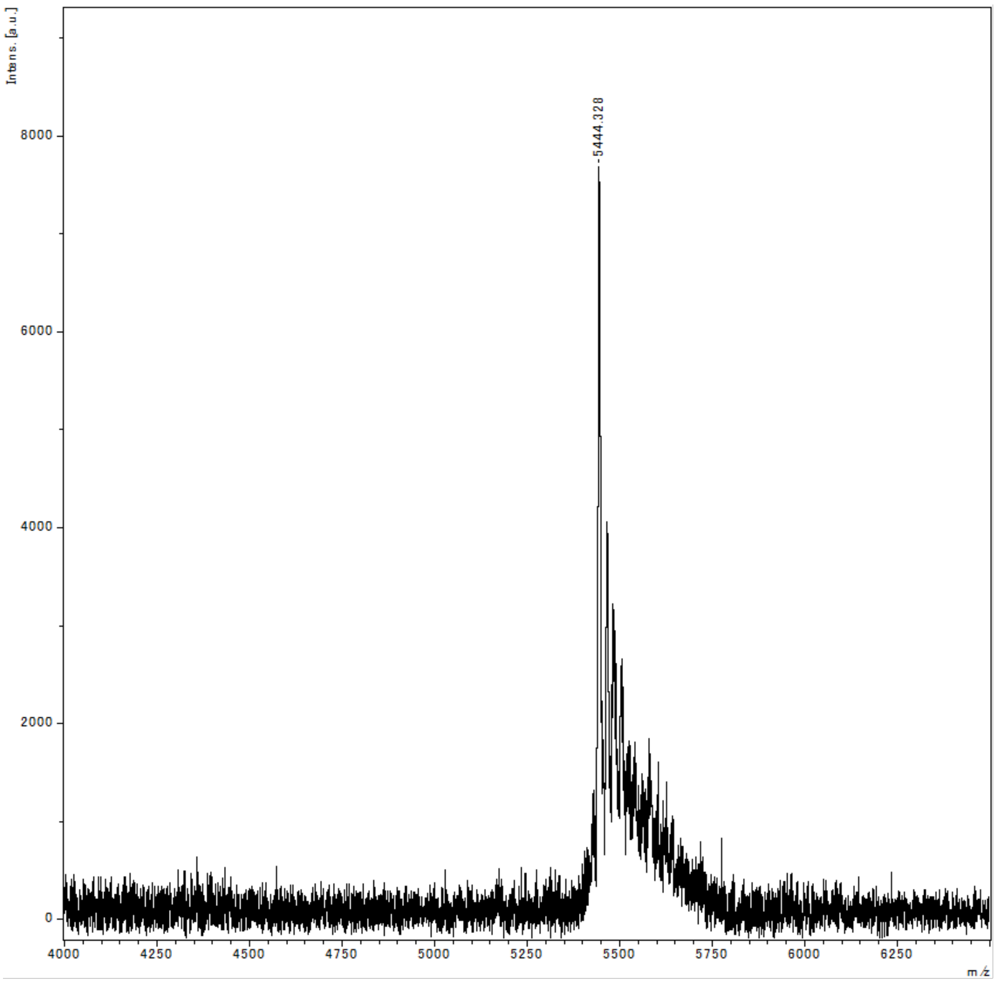

HPLC (**ON27**)

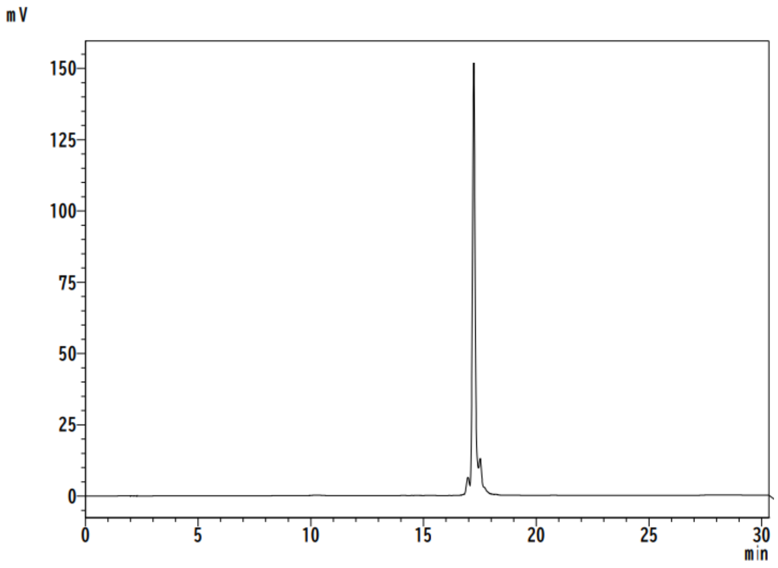

MALDI-TOF MS (**ON27**)

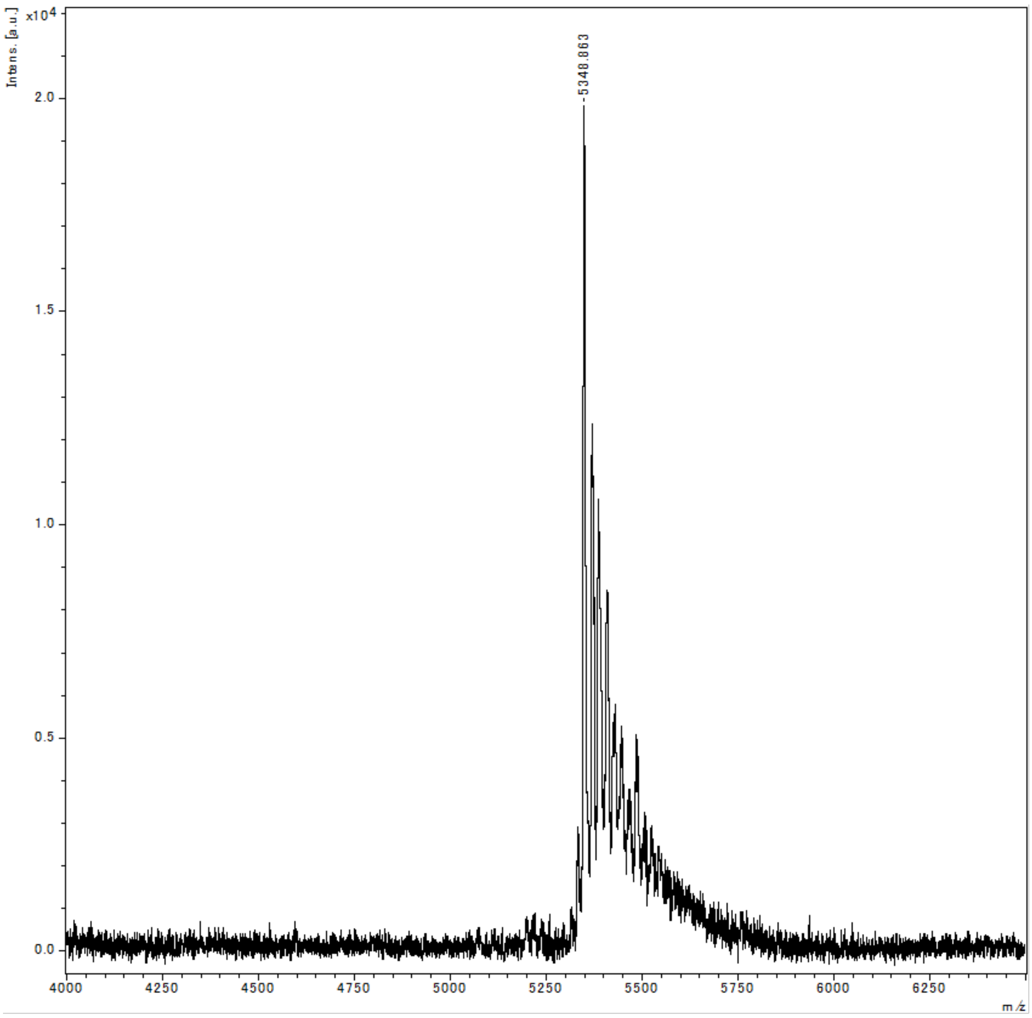

HPLC (**ON28**)

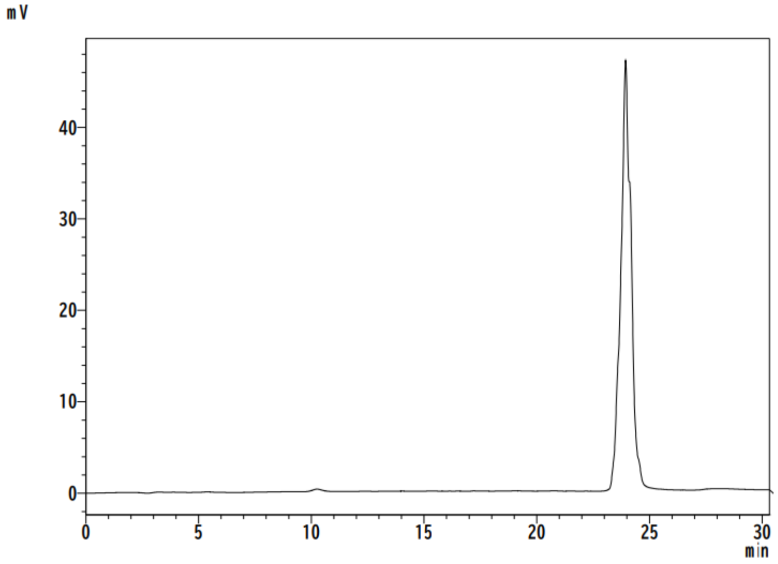

MALDI-TOF MS (**ON28**)

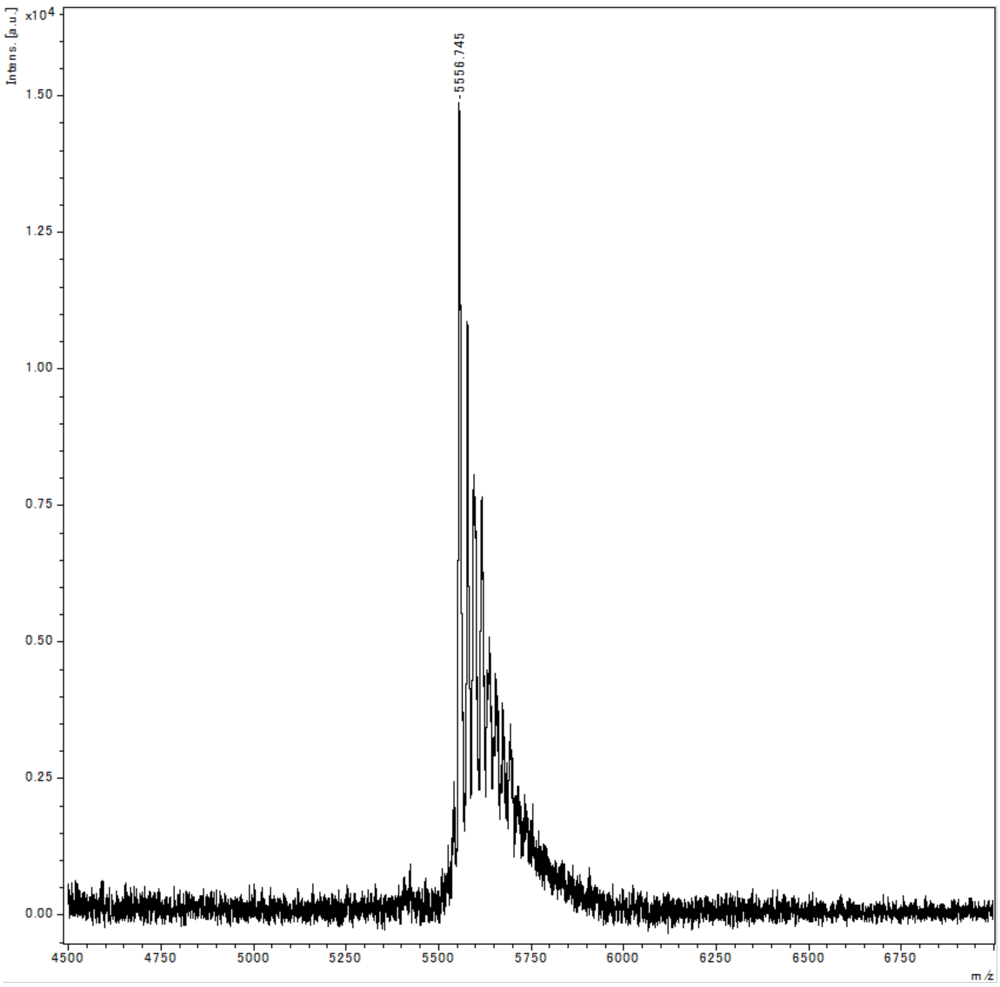

HPLC (**ON29**)

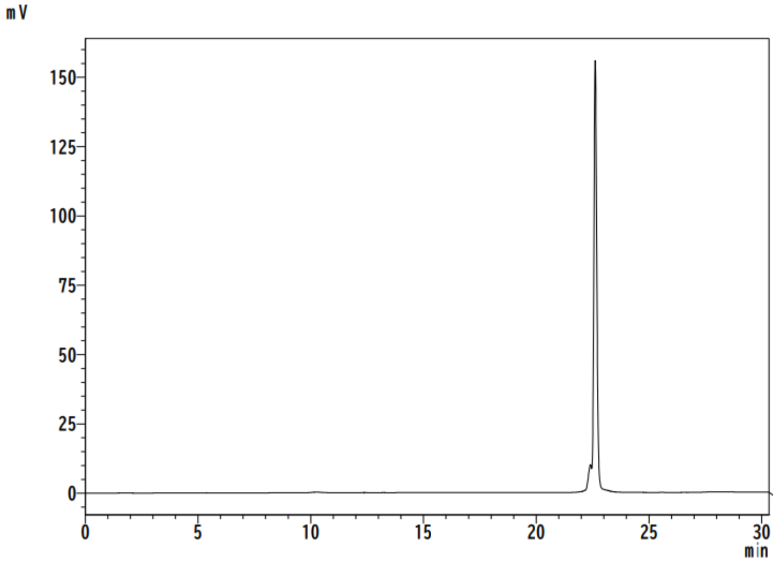

MALDI-TOF MS (**ON29**)

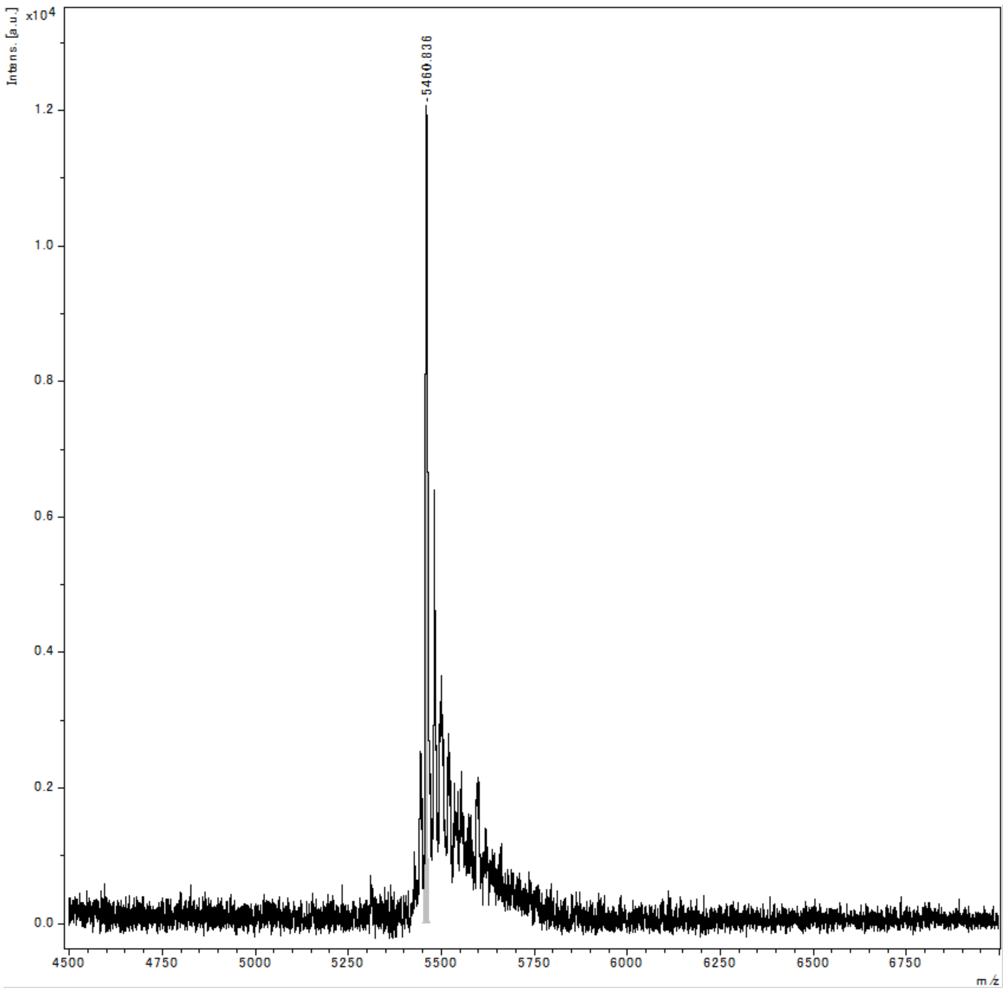

HPLC (**ON34**)

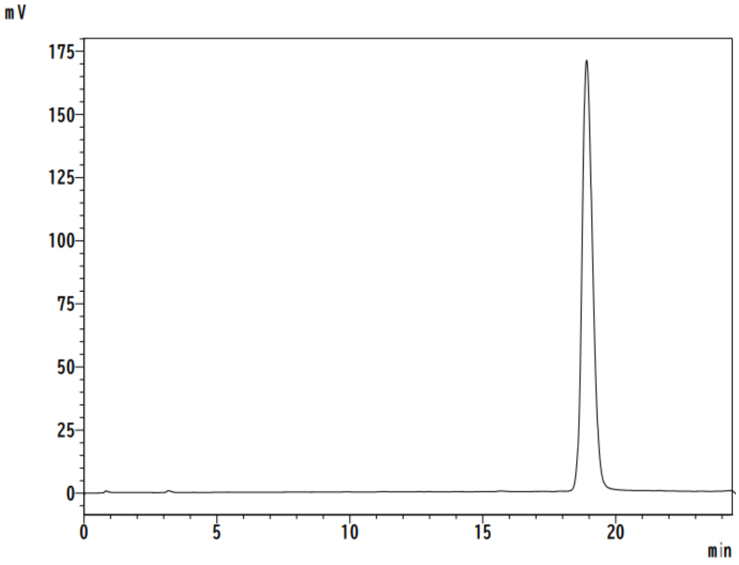

MALDI-TOF MS (**ON34**)

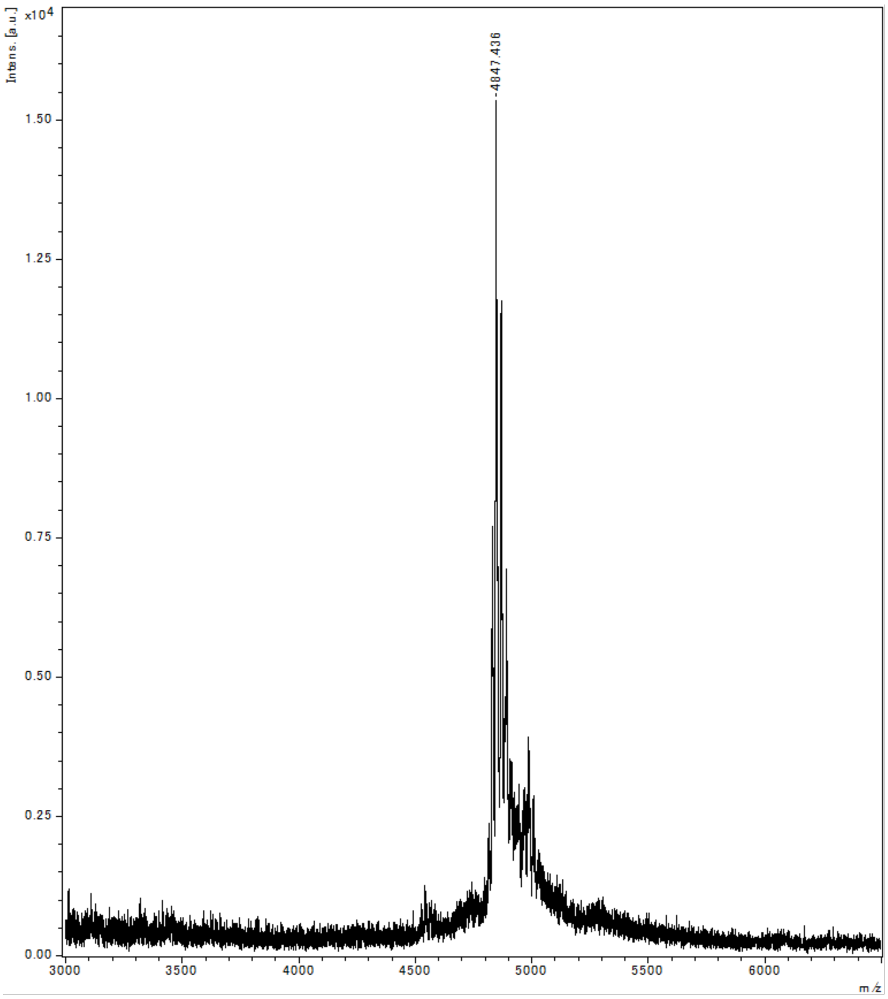

HPLC (**ON35**)

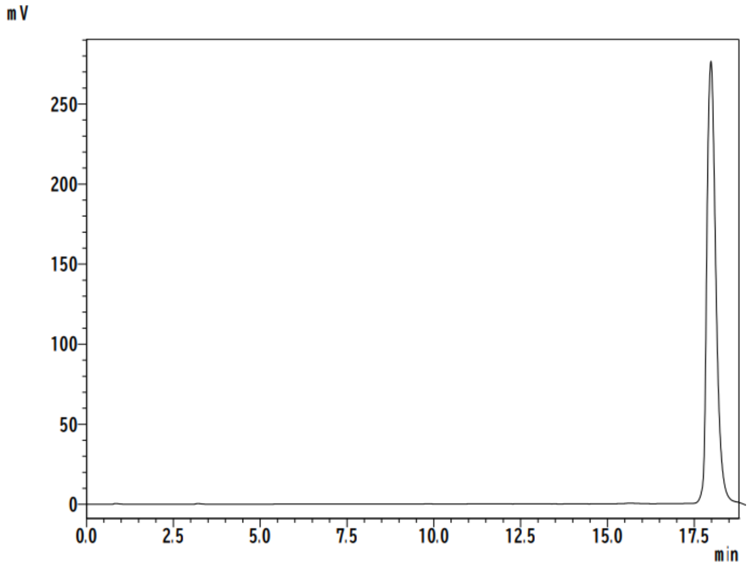

MALDI-TOF MS (**ON35**)

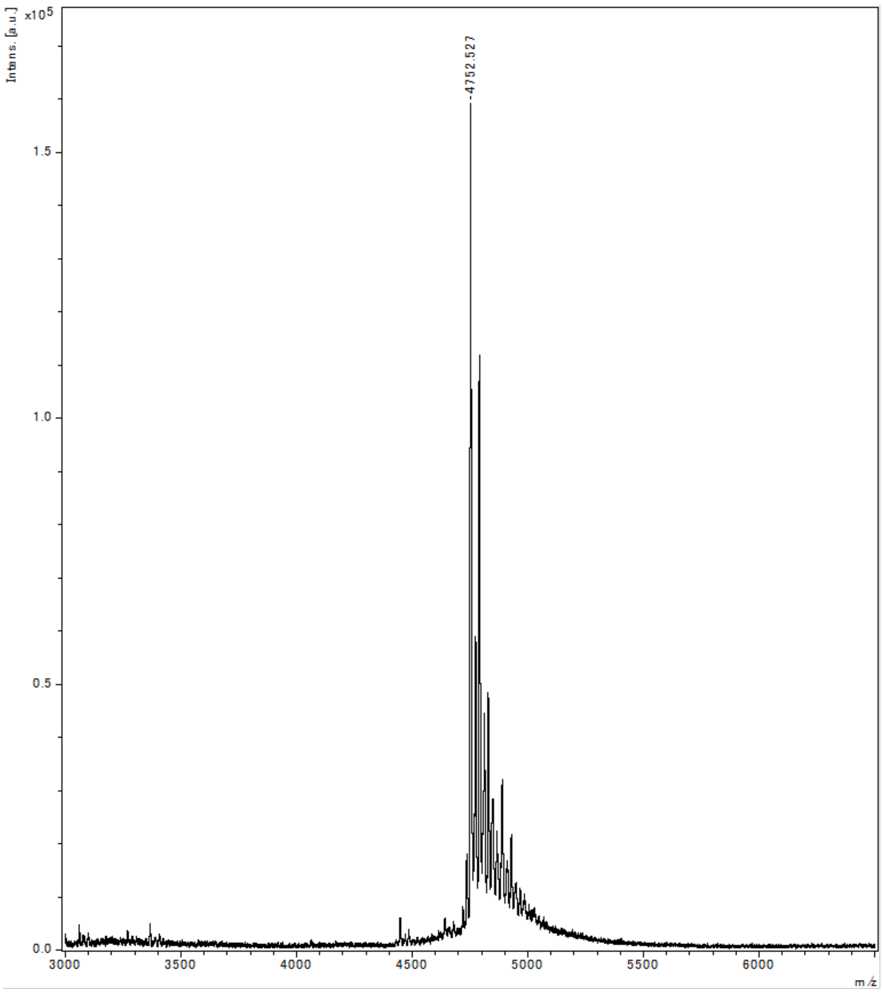

Supplement: Supplementary file 1 [file au5c01005_si_001.pdf]
